# The population impact of herpes simplex virus type 2 (HSV-2) vaccination on the incidence of HSV-2, HIV and genital ulcer disease in South Africa: a mathematical modelling study

**DOI:** 10.1016/j.ebiom.2023.104530

**Published:** 2023-03-16

**Authors:** Jack Stone, Katharine Jane Looker, Romain Silhol, Katherine Mary Elizabeth Turner, Richard Hayes, Jenny Coetzee, Stefan Baral, Sheree Schwartz, Philippe Mayaud, Sami Gottlieb, Marie-Claude Boily, Peter Vickerman

**Affiliations:** a Population Health Sciences, Bristol Medical School, University of Bristol, Bristol, UK; b MRC Centre for Global Infectious Disease Analysis, School of Public Health, Imperial College London, London, UK; c Bristol Veterinary School, University of Bristol, Bristol, UK; d Department of Infectious Disease Epidemiology, Faculty of Epidemiology and Population Health, London School of Hygiene & Tropical Medicine, London, UK; e Perinatal HIV Research Unit, Faculty of Health Sciences, University of the Witwatersrand, Johannesburg, South Africa; f South African Medical Research Council, Cape Town, South Africa; g Department of Epidemiology, Johns Hopkins Bloomberg School of Public Health, Baltimore, MD, USA; h Department of Clinical Research, Faculty of Infectious and Tropical Diseases, London School of Hygiene & Tropical Medicine, London, UK; i Department of Sexual and Reproductive Health and Research, World Health Organization, Geneva, Switzerland; j NIHR Health Protection Research Unit in Behavioural Science and Evaluation at University of Bristol, Bristol, UK

**Keywords:** HSV-2, HIV, Vaccine, Mathematical modelling, South Africa

## Abstract

**Background:**

Evidence suggests HSV-2 infection increases HIV acquisition risk and HIV/HSV-2 coinfection increases transmission risk of both infections. We analysed the potential impact of HSV-2 vaccination in South Africa, a high HIV/HSV-2 prevalence setting.

**Methods:**

We adapted a dynamic HIV transmission model for South Africa to incorporate HSV-2, including synergistic effects with HIV, to evaluate the impact of: (i) cohort vaccination of 9-year-olds with a prophylactic vaccine that reduces HSV-2 susceptibility; (ii) vaccination of symptomatically HSV-2-infected individuals with a therapeutic vaccine that reduces HSV shedding.

**Findings:**

An 80% efficacious prophylactic vaccine offering lifetime protection with 80% uptake could reduce HSV-2 and HIV incidence by 84.1% (95% Credibility Interval: 81.2–86.0) and 65.4% (56.5–71.6) after 40 years, respectively. This reduces to 57.4% (53.6–60.7) and 42.1% (34.1–48.1) if efficacy is 50%, 56.1% (53.4–58.3) and 41.5% (34.2–46.9) if uptake is 40%, and 29.4% (26.0–31.9) and 24.4% (19.0–28.7) if protection lasts 10 years. An 80% efficacious therapeutic vaccine offering lifetime protection with 40% coverage among symptomatic individuals could reduce HSV-2 and HIV incidence by 29.6% (21.8–40.9) and 26.4% (18.5–23.2) after 40 years, respectively. This reduces to 18.8% (13.7–26.4) and 16.9% (11.7–25.3) if efficacy is 50%, 9.7% (7.0–14.0) and 8.6% (5.8–13.4) if coverage is 20%, and 5.4% (3.8–8.0) and 5.5% (3.7–8.6) if protection lasts 2 years.

**Interpretation:**

Prophylactic and therapeutic vaccines offer promising approaches for reducing HSV-2 burden and could have important impact on HIV in South Africa and other high prevalence settings.

**Funding:**

10.13039/100004423WHO, 10.13039/100000060NIAID.

**Research in context**

**Evidence before this study**

To update our existing systematic review of modelling studies of HSV-2 vaccination, we searched PubMed for studies published between March 1st 2017 up to September 2nd 2022, for “(HSV OR herpes simplex) AND (vaccin∗) AND (model∗)”, with no restrictions on language. Overall, there have been 11 mathematical modelling studies evaluating the impact of HSV-2 vaccines. Of these, seven studies modelled prophylactic vaccines only, two studies modelled therapeutic vaccines only, and two studies modelled both vaccine types. The majority of studies considered North America or unspecified settings. Most studies only considered the impact of vaccination on HSV-2 incidence. Only one study, which modelled only prophylactic vaccines, evaluated the potential impact of vaccination on HIV incidence. No studies have evaluated the impact of vaccination on disease sequalae.

**Added value of this study**

Our model showed that large (>80%) reductions in HSV-2 incidence, and up to a two-third reduction in HIV incidence, after 40 years, are possible in South Africa for a prophylactic HSV-2 vaccine with high efficacy/uptake. However, considerable gains would be made even with a vaccine offering imperfect protection, potentially improving the health and wellbeing of millions of people. A therapeutic HSV-2 vaccine would have a generally smaller, but nevertheless still substantial impact, and compared to a prophylactic vaccine, a much greater impact per vaccination. Our study adds greatly to the currently sparse modelling literature on HSV-2 vaccine impact for low-and-middle-income countries and therapeutic vaccines, incorporating HIV-HSV-2 interactions, and HSV-2 disease sequalae (time with genital ulcer disease [GUD]).

**Implications of all the available evidence**

Imperfect HSV-2 vaccines, whether prophylactic or therapeutic, could have important public health impacts on HSV-2 incidence. Both vaccines could also have important indirect impacts on reducing HIV incidence in high HIV prevalence settings – although this has only been evaluated in three settings across two studies. With widespread use, prophylactic vaccines have the potential to avert a greater number of HSV-2 and HIV infections and person-years, but therapeutic vaccines may be more efficient (i.e., avert more per vaccination) than prophylactic vaccines; only a limited number of studies have compared potential impacts of both vaccines.

## Introduction

HSV-2 infection can lead to several disease manifestations, most notably recurrent genital ulcer disease (GUD). GUD is estimated to affect 16% of women and 9% of men annually in the WHO Africa region.^1^ South Africa has one of the highest rates of HSV-2 infection worldwide (50% prevalence among 15- to 44-year-olds^2^) and the largest population of people living with HIV (PLWH) globally.

This double burden in South Africa is especially concerning as there is evidence that HIV and HSV-2 infections interact biologically.^3^ One such ‘cofactor effect’ is the role of HSV-2 infection in increasing susceptibility to HIV,^3^ which could contribute 37.1–52.3% of sexually-acquired HIV infections in the WHO Africa region.^4^^,^^5^ HIV and HSV-2 also seem to increase transmissibility of the other in coinfected individuals.^6^

Interventions against HSV-2 could have considerable health impact through direct effects on HSV-2 transmission and/or GUD and indirect effects on HIV transmission. The World Health Organization (WHO) has identified an effective HSV-2 vaccine, either prophylactic that prevents infection or therapeutic that reduces symptoms and infectivity, as a key public health goal.^7^ However, although some trials of prophylactic vaccines have shown promise,^7^ no products have been proven to have efficacy against HSV-2 infection or disease. Conversely, several therapeutic vaccine candidates have recently completed phase I/II trials with one candidate achieving 65% efficacy in reducing GUD frequency and shedding.^8^ However, none have been taken further.^7^

In 2015, WHO convened an expert meeting to review existing models assessing the population-level impact of HSV vaccination to determine future modelling needs.^9^ The meeting highlighted the following under-researched areas: (a) impact of vaccines in low- and middle-income countries, (b) impact of therapeutic vaccines, (c) effect of HIV-HSV-2 cofactors on vaccine impact on HIV, and (d) vaccine impact on HSV-2-related disease outcomes.^9^ To address these needs, we modelled the population impact of HSV-2 prophylactic or therapeutic vaccination on HSV-2 and HIV infection and HSV-2 GUD in South Africa.

## Methods

### Model description

We adapted an existing dynamic model of HIV transmission in South Africa^10^ to incorporate the natural history of HSV-2 infection and HSV-2 vaccination. Full model equations and schematics are in the Supplementary materials (Sections 1 and 2). The model considers individuals aged 15–49 years, divided into six discrete sub-populations: females and males at low-risk of HIV acquisition, female sex workers (FSWs), male clients of FSWs, and young (<30 years) and older (≥30 years) men who have sex with men (MSM). The model incorporates population growth and transitions between risk groups. The model captures HIV and HSV-2 transmission through vaginal and anal intercourse between all males and females, and anal intercourse among MSM, including transmission due to main, casual, and commercial partnerships. HIV and HSV-2 acquisition risk are determined by sexual behaviour, HIV and HSV-2 infection status among partners and male circumcision status.

**Fig. 1:**
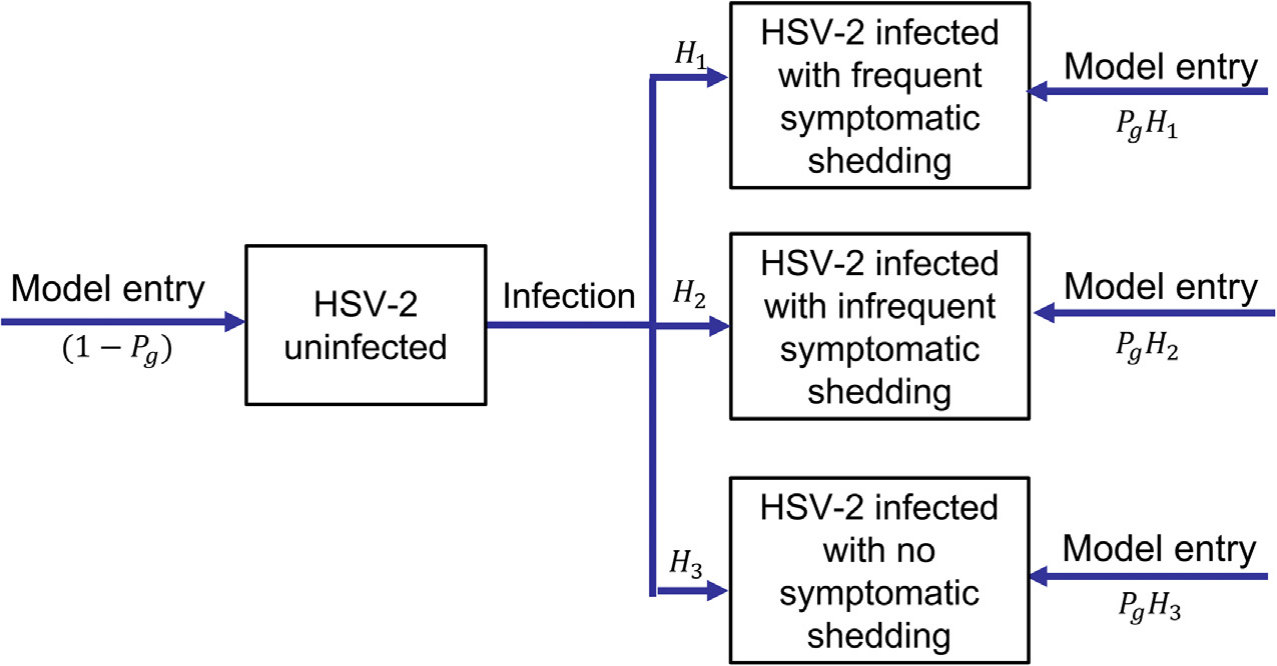
Model schematics illustrating the stratification of the population with respect to HSV-2 infection and GUD. H_1_, H_2_ and H_3_ denote the percentage of new infections that are allocated into the three groups of HSV-2-infected individuals, which differ according to their rates of symptomatic shedding but have the same rates of asymptomatic shedding. P_g_ denotes the percentage of individuals entering the model who are infected with HSV-2 which differs by gender (g).

The model simulates HIV transmission, disease progression and the effect of ART, which reduces HIV-related disease progression and mortality^10^ and infectivity. The model also simulates the natural history of HSV-2 infection, with HSV-2 infected individuals assumed to develop and remain in one of three levels of symptomatic disease (Fig. 1; Supplementary Materials Section 3): (group 1) high percentage of days with GUD and associated shedding, (group 2) low percentage of days with GUD and associated shedding, or (group 3) no reported GUD and only asymptomatic shedding. HSV-2 transmission is assumed to occur during shedding periods only, with HSV-2 transmission risk being higher during symptomatic shedding.^11^ Lastly, some individuals are infected with HIV and/or HSV-2 on model entry at age 15.

The model incorporates the HIV-HSV-2 cofactor effects (Table 1) best supported by data: (i) HSV-2 infection increases HIV acquisition risk, (ii) HSV-2 infection increases HIV transmission risk, and (iii) HIV infection increases HSV-2 transmission risk. For (i) and (ii), we modelled an increased risk of HIV acquisition/transmission if HSV-2 infected which is further elevated during GUD periods. For (iii), we incorporated the effect of HIV co-infection on increasing the days with HSV-2 shedding and the effect of ART on reducing this (Table 2).

**Table 1:** Prior values for parameters related to HSV-2 transmission and cofactor effects.

Parameter	Symbol	Value	Sampling distribution	Notes/references
Percentage of males who are HSV-2-infected when entering the model at 15 years old	$\delta_{HSV,m}$	0.7% (95% CI: 0.08–2.4)	Normal	Suggested by empirical estimates of HSV-2 prevalence among high-school students^12^
Percentage of females who are HSV-2-infected when entering the model at 15 years old	$\delta_{HSV,f}$	4.1% (95% CI: 2.6–6.0)	Normal	Suggested by empirical estimates of HSV-2 prevalence among high-school students^12^
Per-act HSV-2 acquisition probability for receptive vaginal intercourse (VI) when the partner is shedding asymptomatically	$\beta_{xv}^{HSV}$	0.0005–0.022	Uniform	13, 14, 15, 16, 17
Relative risk of acquiring HSV-2 from receptive VI vs insertive VI	$\frac{\beta_{yv}^{HSV}}{\beta_{xv}^{HSV}}$	1–3	Uniform	^16^^,^^18^
Relative risk of acquiring HSV-2 from insertive anal intercourse (AI) vs insertive VI	$\frac{\beta_{ya}^{HSV}}{\beta_{yv}^{HSV}}$	1–2	Uniform	Same prior as HIV assumed
Relative risk of acquiring HSV-2 from receptive AI vs receptive VI	$\frac{\beta_{xa}^{HSV}}{\beta_{xv}^{HSV}}$	2–18	Uniform	Same prior as HIV assumed
Per-act efficacy of condom use in reducing HSV-2 acquisition	$\varepsilon_{HSV}$	30.0% (95% CI: 6.0–60.0)	Lognormal	^18^
Per-act efficacy of male circumcision in reducing HSV-2 acquisition	$\vartheta_{HSV}$	20.7% (95% CI: 3.5–34.8)	Lognormal	Pooled results from 3 RCTs^19^
Relative per-act risk of HSV-2 transmission during GUD episodes (vs asymptomatic shedding)	$R{R_{\beta }}^{HSV|GUD}$	1.44 (1–3)	Triangular	^17^^,^^20^^,^^21^According to Schiffer et al., 76% of transmissions occurred during asymptomatic periods. Combined with our estimates of the overall percentage of days with asymptomatic shedding, and GUD (among HIV-uninfected populations) yields an RR of 1.44 (on average 76% of transmissions occur over 41 days of asymptomatic shedding, whereas the remaining 24% of transmissions occur over 9 days of symptomatic shedding, thus one day of symptomatic shedding results in a 1.44-fold higher probability of transmission)
**Per-act cofactor effects (cofactors increasing percentage of days with shedding are described in**Table 2**)**				
Relative risk of per-act HIV transmission due to HSV-2 coinfection (vs HSV-2-uninfected)	$R{R_{\beta }}_{s}^{tHIV|HSV}$	1.33 (range 1.00–1.93)	Triangular	^5^
Relative risk of per-act HIV transmission during GUD episodes (vs non-GUD HSV-2 infection)	$R{R_{\beta }}_{s}^{tHIV|GUD}$	2.58 (range 1.03–5.69)	Lognormal	^22^As the study sample was all PLWH, we assumed that it was more likely to also be HSV-2-infected, and assumed that this relative risk among those with GUD episodes should be compared to non-GUD HSV-2 infection
Relative risk of per-act HIV acquisition due to HSV-2 infection during non-GUD HSV-2 (vs HSV-2-uninfected)	$R{R_{\beta }}_{s}^{aHIV|HSV}$	1–5	Uniform	^3^Calibrated using HSV-2 and HIV co-infection prevalences
Relative risk of per-act HIV acquisition during GUD episodes (vs non-GUD HSV-2 infection)	$R{R_{\beta }}_{s}^{aHIV|GUD}$	1–3	Uniform	Conservative range based on the ratio between the point estimate of the relative risk of HIV acquisition during GUD vs no STI = 5.29 (95%CI: 1.43–19.58)^23^ divided by the relative risk of HIV acquisition if HSV-2-infected^3^ = 2.75

**Table 2:** Prior values for parameters related to asymptomatic and symptomatic shedding among HSV-2-infected individuals.

Parameter	Estimate (prior range (min–max))			Notes/references^a^
	HSV-2-infected with frequent symptomatic shedding (s = 1)	HSV-2-infected with infrequent symptomatic shedding (s = 2)	HSV-2-infected with no symptomatic shedding (s = 3)	
Percentage of new infected individuals s in each shedding group (H_s_)	12.6% (9.6–16.3)	24.6% (12.3–41.3)	62.8% (42.4–78.1)	H_1_ is based on US data (NHANES 2007–2010) for the percentage of those HSV-2 seropositive who are diagnosed: 12.6% (95% CI: 9.6–16.3),^24^ which we assume are symptomatic. This refers to the percentage to which clinic data (i.e., frequent symptomatic shedding) can be applied.^1^*H*_2_ was estimated from the estimated percentage of individuals with unrecognised infection (*H*_2_ + *H*_3_) who have recurrences from a meta-analysis^1^ of 3 studies, and H_3_ is the remainder.
Percentage of days with asymptomatic shedding among HIV-uninfected individuals	11.2% (2–33)	11.2% (2–33)	11.2% (2–33)	Weighted mean (by sample size) of the % of days with asymptomatic shedding across available estimates is 11.2%. Range over studies is 2–33%.^1^ In empirical studies, the % of days with asymptomatic shedding is similar among those who have vs those who do not have a history of symptomatic episodes.^25^^,^^26^ We therefore assumed the same rate of asymptomatic shedding across the three groups.
Average number of symptomatic episodes per year	5.0 (4.3–5.9)	5.0 (4.3–5.9)	0.0	The pooled^1^ (N = 18 studies) annual number of symptomatic episodes among clinic populations is 5.0 (95% CI: 4.3–5.9). The pooled^1^ (N = 5 studies) annual number of symptomatic episodes among individuals with unrecognised infection is 3.4 (95% CI: 2.7–4.4). However, only a percentage of individuals with unrecognised infection (reflecting *H*_2_ and *H*_3_ groups) will have symptoms (group *H*_2_).^11^^,^^25^ Fitting to this percentage and the average duration of a symptomatic episode, we found a similar average number of episodes between the groups *H*_1_ and *H*_2_.
Average duration of a symptomatic episode (days)	8.5 (7.5–9.5)	3.0 (1.6–5.7)	0.0	Pooled^1^ (N = 13 studies) average duration of symptomatic recurrences among clinic populations is 8.5 days (95% CI: 7.9–9.5). Among a US study of individuals with unrecognised infection, the average duration of recurrent symptomatic episodes was 3.0 days (95% CI: 1.6–5.7).^1^^,^^26^
Relative risk in days of asymptomatic shedding if untreated HIV-infected vs HIV-uninfected	2.16 (1.09–4.28)			No estimate was directly available, so we used a pooled estimate of the relative risk of all shedding if untreated HIV-infected vs HIV-uninfected from^27^: (2.16 (95% CI: 1.09–4.28), N = 3 studies).
Relative risk in days of symptomatic shedding if untreated HIV-infected vs HIV-uninfected	2.78 (1.51–5.14)			No estimate was directly available, so we used a pooled estimate of the relative risk of symptomatic shedding if untreated HIV-infected vs HIV-uninfected from^27^: (2.78 (95% CI: 1.51–5.14), N = 3 studies).
Relative risk in days of shedding (asymptomatic or symptomatic) if treated vs untreated PLWH	0.56 (0.41–0.77)			Comparing treated PLWH to untreated PHLIV, OR for any shedding is 0.56 (95% CI: 0.41–0.77). This reduction was applied to the relative risk described above in order to ensure that treated PLWH always shed HSV-2 more frequently than HIV-uninfected individuals.

a References for studies included in pooled estimates are given in Supplementary Table S1.

#### HSV-2 vaccination

In prophylactic vaccine scenarios, we model annual vaccination of 9-year-olds (to align with HPV vaccination in South Africa) from 2020 (“cohort vaccination”). This was implemented as a percentage (“uptake”) of individuals entering the model at age 15 years into the vaccinated and protected compartment from 2026 onwards (Supplementary Fig. S2a). Prophylactic vaccination is assumed to reduce an individual's susceptibility to infection, and potentially provide additional therapeutic benefits (i.e., reduction in shedding; in sensitivity analysis) following breakthrough infections, with waning protection (when modelled), resulting in individuals transitioning to an “ever vaccinated but without protection” compartment at a constant rate.

In therapeutic vaccine scenarios, symptomatic HSV-2-infected individuals (groups 1 and 2 above) are vaccinated at a constant rate (Supplementary Fig. S2b), with the likelihood of vaccination being proportional to their time with GUD, i.e., people who experience more disease (group 1) are more likely to present to care and so be offered vaccination than those with less disease (group 2). Consequently, those coinfected with HIV are more likely to be vaccinated than those with HSV-2 mono-infection. Therapeutic vaccination is assumed to reduce days with viral shedding (asymptomatic and symptomatic) and potentially infectivity when shedding, with waning of protection (when modelled) resulting in individuals transitioning to an “ever vaccinated but without protection” compartment at a fixed rate. Boosters can prevent loss of protection in a proportion of vaccinated individuals.

### Model parameterisation and calibration

The demographic, behavioural and HIV natural history aspect of the model was parameterised as before^10^ (Supplementary Materials Section 4). Model parameterisation was informed by national and subnational general population surveys, multi-city FSW and MSM surveys and surveys of clients of FSWs. HSV-2 parameters were parameterised according to Tables 1 and 2. Condom use was assumed to increase over time (differentially for each group) and to differ based on partnership type and type of sex. Male circumcision was also assumed to increase over time. Time trends in adult ART coverage for South Africa came from UNAIDS. Based on self-reported ART use and viral suppression data from multiple key population surveys, we assumed lower ART coverage among these groups than overall among adult males and females. The model, coded in Matlab, was initialised in 1985, with stable population and HSV-2 dynamics (at pre-1985 levels of condom use) and HIV seeded at low prevalence (≤0.5%). Model parameters were varied to optimise the agreement of the model, or ‘calibrated’, to detailed epidemiological data from South Africa using approximate Bayesian computation Sequential Monte Carlo methods (Supplementary Materials Section 5). The model was cross-validated using available HIV and HSV-2 incidence and prevalence data not used in model calibration. We also compared the modelled increase in HIV incidence by HSV-2 status to pooled adjusted estimates of the incidence rate ratio (IRR) from a recent systematic review^3^ (aIRR: 2.5; 95% CI 1.8–3.4 among females and 3.1, 95% CI 2.2–4.3 among males).

## Model analyses

### Proportion of HIV infections attributable to HSV-2

We estimated the proportion of incident HIV infections attributable to HSV-2 over 2020–2029 (10-year transmission population attributable fraction, ‘tPAF’^28^) by comparing the cumulative number of infections over 2020–2029 in the baseline model with a counterfactual scenario in which being HSV-2 infected does not elevate HIV transmission or acquisition over 2020–2029, i.e., the cofactors that result in HSV-2 increasing transmission or acquisition risk are set to 1 over this time period.

### Vaccination impact analyses

We assessed the impact of different therapeutic and prophylactic vaccines and vaccination scenarios on HSV-2 and HIV incidence and time with GUD. For each, we measured the impact as the relative reduction in the rate of new infections or GUD days after 20 or 40 years from 2020 vs the status quo. In status quo and vaccination scenarios, we modelled stable coverages of existing interventions (ART, male circumcision and condom use) from 2020. Results for GUD were presented as the reduction in GUD days in order to fully capture the impact of vaccination on time spent with disease. We also calculated the number needed to vaccinate (NNV) to avert one HSV-2 infection, one person-year of GUD, and one HIV infection over 2020–2060, and projected cumulative HSV-2/HIV infections and HSV-2/HIV prevalence.

#### Prophylactic vaccination scenarios

We first modelled scenarios whereby vaccines provide lifelong protection against HSV-2 acquisition assuming a degree type efficacy (defined as the percentage reduction in risk of infection among those vaccinated – i.e., all vaccinated individuals are protected to some extent, but only a percentage of challenges in vaccinated individuals are protected against) of 50%, 80% or 100%. For each efficacy, we modelled yearly uptakes of 40%, 60% (comparable to HPV vaccine uptake^29^) or 80% in the age 9 years cohort.

Sensitivity analyses then considered how the impact of vaccination would change if we assumed: take type efficacies (defined as the percentage of vaccinated individuals that are protected against all challenges, with the remainder having no protection – i.e., protection is ‘all or nothing’) of 50%, 80% or 100% (rather than degree type efficacy); shorter durations of protection (10 or 20 years); therapeutic benefits among individuals with breakthrough infections^7^ (50% reduction in shedding days); and if the vaccination schedule also included a one-off catch-up of those aged 10–14 years with 40% uptake in 2020.

#### Therapeutic vaccination scenarios

We first modelled scenarios whereby vaccines provided lifelong protection and reduced the percentage of days with shedding with degree type efficacies of 50%, 80% and 100%, and vaccination rates which result in 40%, 60% or 80% coverages (i.e., proportion ever vaccinated) among those with symptomatic infection in 2060 (i.e., after 40 years).

Sensitivity analyses then considered how the impact would change if we assumed: take type efficacies of 50%, 80% or 100% (rather than degree type efficacy); shorter durations of protection (2, 5, or 10 years) with 0 or 50% of vaccinated individuals receiving boosters (every 2, 5 or 10 years to maintain protection); the vaccine was only effective at reducing time with symptomatic, not asymptomatic, shedding; the vaccine also halved HSV-2 infectivity; and if vaccination was expanded to 40% of all HIV-HSV-2-co-infected individuals.

### Ethical approval

Ethical approval was not required because patients and the public were not involved in this study.

### Role of funders

S.G. from WHO commissioned the study, contributed to the study design, helped with the interpretation of the results, and edited and commented on the draft manuscripts. NIAID had no role in the study. The corresponding author had full access to all the data and had final responsibility for the decision to submit.

## Results

### Status quo projections and contribution of HSV-2 to HIV transmission

**Fig. 2:**
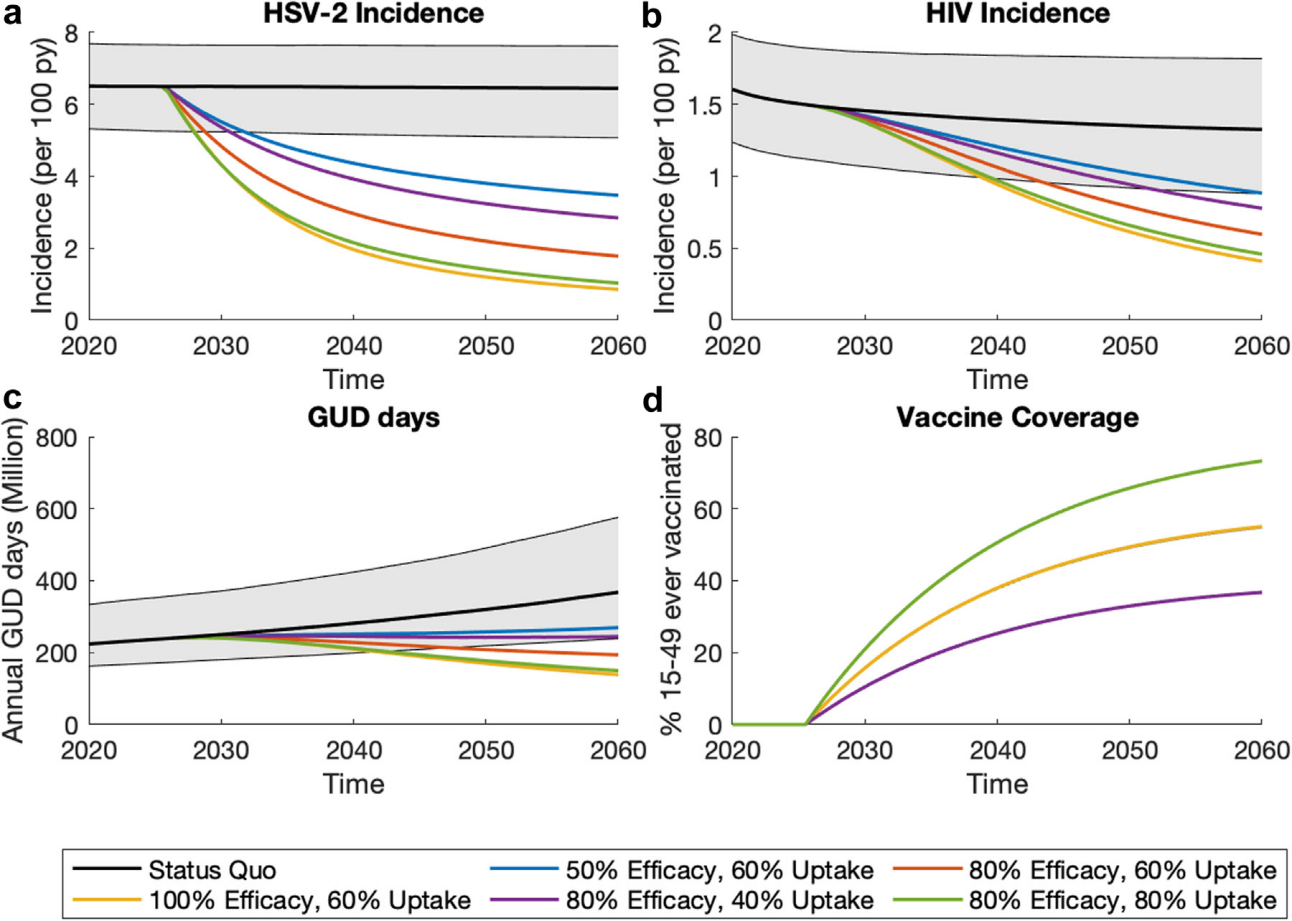
Model projections of the impact of a prophylactic vaccine on (a) HSV-2 incidence among 15- to 49-year-olds; (b) HIV incidence among 15- to 49-year-olds; (c) Annual number of days with GUD; (d) % 15- to 49-year-olds that have ever been vaccinated. Coloured lines show median projections for vaccinating a proportion (the uptake) of 9-year-olds each year with a prophylactic vaccine which has lifelong protection and provides protection against HSV-2 acquisition. Black lines and grey shading area show the median and 95% CrI for the status quo scenario.

Supplementary Figs. S3–S6 show the model agreed well with calibration data and cross-validation data on HIV and HSV-2 prevalence and incidence. Status quo projections (Fig. 2; Supplementary Fig. S3) suggest a stable HSV-2 epidemic, with HSV-2 prevalence (among 15- to 49-year-olds) of 36.2% (95% Credibility Interval, CrI: 30.9–39.2) in men and 62.8% (95% CrI: 56.6–68.6) in women in 2020. Conversely, the model projects a slow decreasing HIV epidemic; with prevalence decreasing from 19.1% (95% CrI: 17.8–20.9) in 2020 to 15.2% (95% CrI:11.7–19.3) in 2060 due to scale-up of ART. Prevalence of HIV-HSV-2 co-infection is similarly slowly decreasing (Supplementary Fig. S7). In 2020, the IRR of HIV by HSV-2 status was 3.7 (95% CrI: 2.8–4.7) among females and 4.6 (95% CrI: 3.6–5.8) among males, comparable to empirical estimates.^3^ HSV-2 is estimated to contribute (tPAF) 70.2% (95% CrI: 62.9–76.1) of new HIV infections over 2020–2029, mostly due to the cofactor effect of being HSV-2 infected increasing HIV acquisition risk (rather than transmission risk) which contributes 64.1% (95% CrI: 57.0–69.6) of new HIV infections.

### Prophylactic vaccine impact

#### Main scenarios

**Fig. 3:**
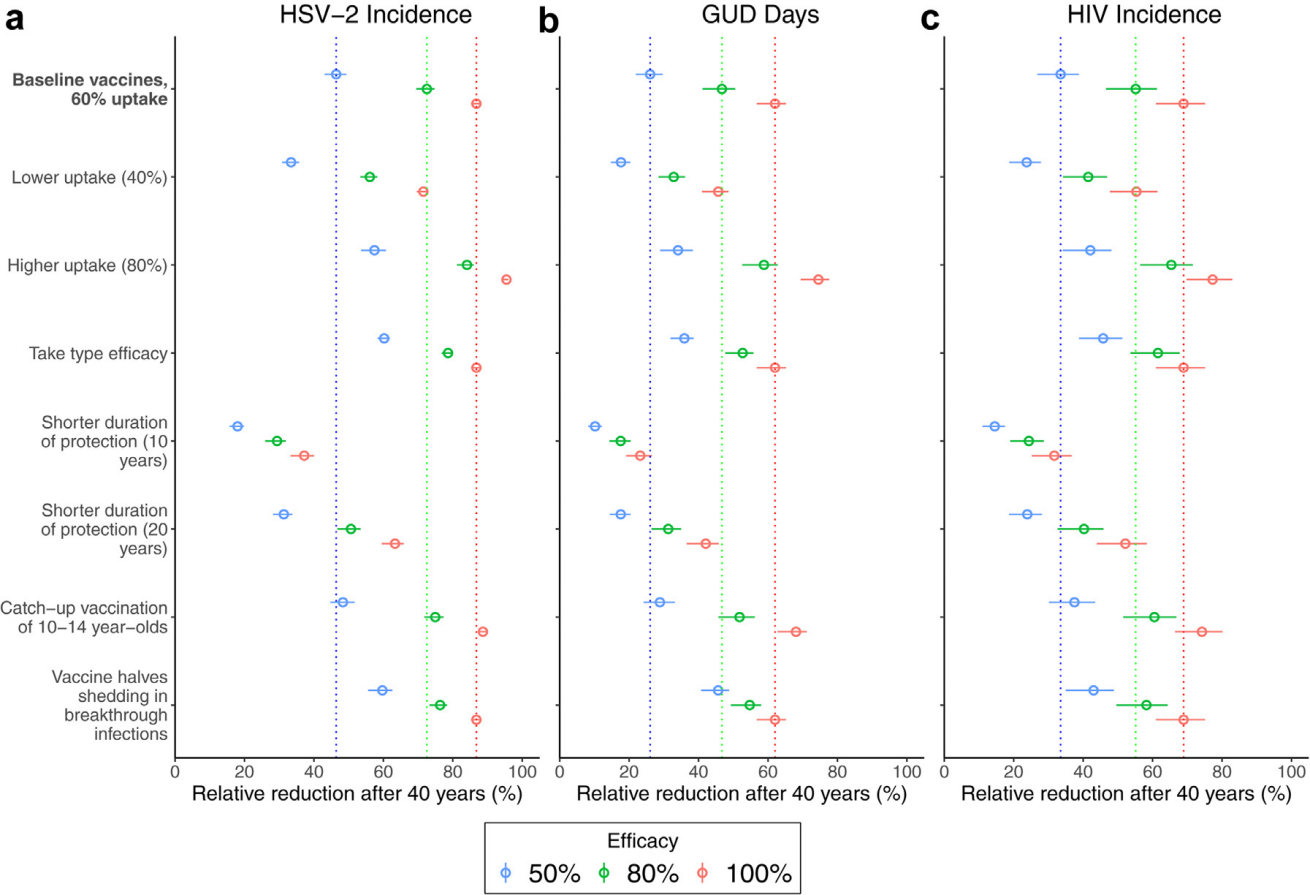
Sensitivity analyses for the 40-year impact of prophylactic vaccination on (a) HSV-2 incidence; (b) annual GUD days; (c) HIV incidence. Figures show how the impact of a prophylactic vaccine would differ in each sensitivity analysis compared to the baseline vaccination scenario (vertical dashed lines for vaccines of different efficacies): vaccinating 60% of 9-year-olds each year with a prophylactic vaccine which has lifelong protection and provides degree type protection against HSV-2 acquisition with 50% (blue), 80% (green) or 100% (red) efficacy. Impact measured as relative reduction by 2060 compared to status quo in 2060. Circles show median projections with error bars showing 95% credibility intervals.

**Fig. 4:**
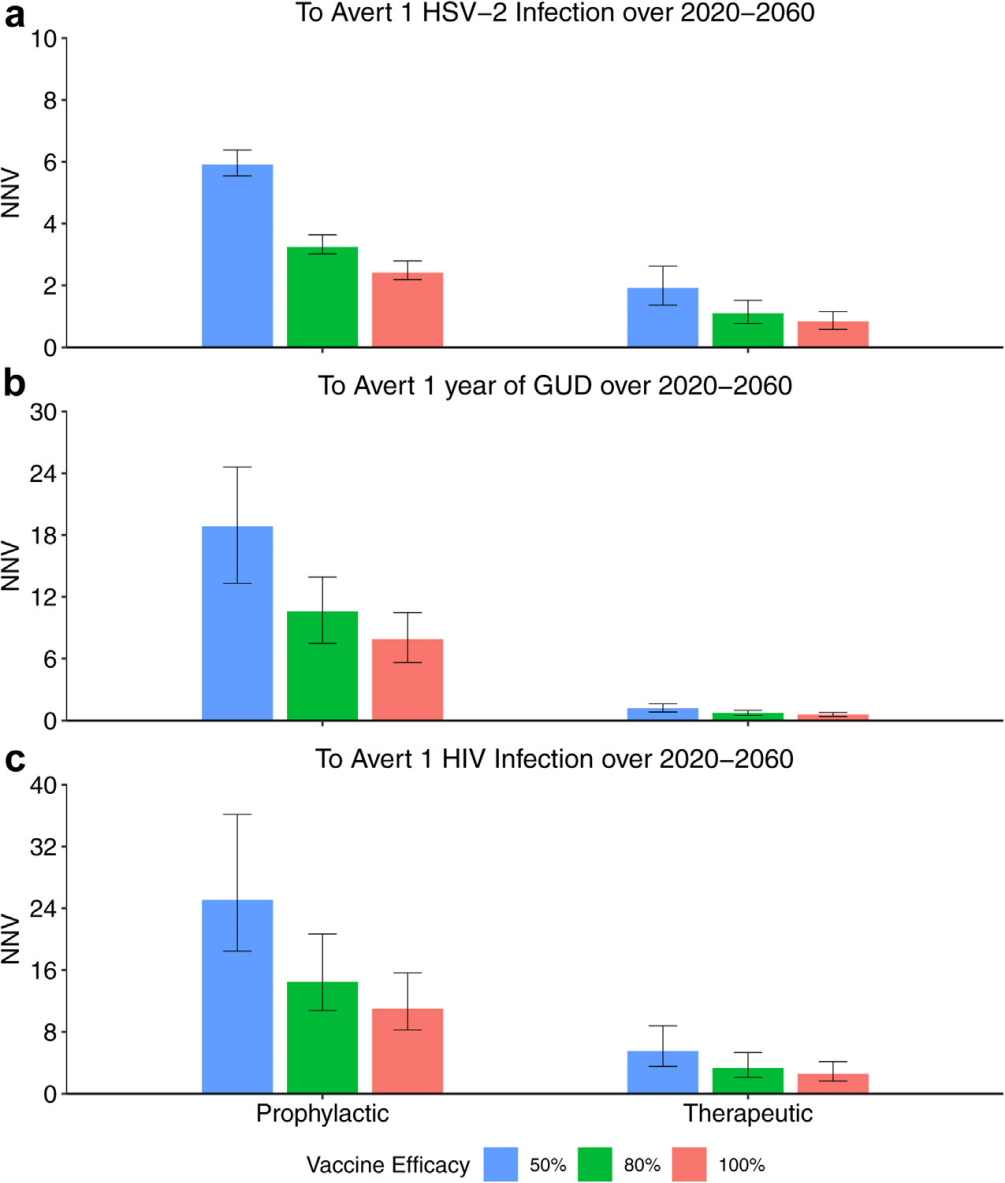
Model projections of the number needed to vaccinate to avert over 2020–2060 (a) one HSV-2 infection; (b) one year of GUD; (c) one HIV infection. Bars show median projections, with error bars showing 95% credibility intervals. Prophylactic vaccine scenarios are vaccinating 60% of 9-year-olds each year with a prophylactic vaccine which has lifelong protection and provides 50/80/100% protection against HSV-2 acquisition. Therapeutic vaccine scenarios are vaccinating HSV-2-infected symptomatic individuals with a therapeutic vaccine to a coverage of 40% after 40 years which has lifelong protection and reduces asymptomatic and symptomatic shedding by 50/80/100%.

Prophylactic vaccines can have substantial impact on HSV-2 incidence (Figs. 2 and 3). With 50% efficacy and 60% uptake, vaccination could reduce HSV-2 incidence by 32.8% (95% CrI: 30.6–34.5), compared to Status quo projections, after 20 years or 46.4% (95% CrI: 43.0–49.4) after 40 years, with these impacts increasing to 66.8% (95% CrI: 63.8–69.0) and 84.1% (95% CrI: 81.2–86.0) with an 80% efficacious vaccine and 80% uptake. Projected reductions in HIV incidence and time with GUD are comparable but smaller than the impact on HSV-2 incidence. For example, with an 80% efficacious vaccine and 80% uptake, after 40 years both time with GUD and HIV incidence are reduced by more than half (58.8% reduction; 95% CrI: 52.6–62.8, and 65.4% reduction, 95% CrI: 56.5–71.6, respectively). At lower efficacies and uptakes, up to 24.6% and 19.6% greater reductions in HIV and HSV-2 incidence would be achieved among women than men (Supplementary Table S8). Projections of cumulative HSV-2/HIV infections and time with GUD averted are in Supplementary Materials Section 6. Fig. 4 shows that with 60% uptake, the NNV to avert one HSV-2 infection, one person-year of GUD, and one HIV infection over 2020–2060 is between 2.4–5.9 (range of medians across different efficacy scenarios), 7.9–18.8, and 11.0–25.1, respectively.

#### Sensitivity analyses

Fig. 3 and Supplementary Fig. S9 show that the impact of prophylactic vaccines are highly sensitive to the duration of protection. Compared to a vaccine with lifelong protection, the 40-year impact on HSV-2 incidence, GUD days and HIV incidence is about halved (54–63% lower, depending on outcome and efficacy) if protection lasts 10 years and about a quarter (24–33%) lower if protection lasts 20 years. At lower efficacies, greater impact is achieved if the vaccine has take type efficacy (30–37% greater impact if 50% efficacy but only 8–13% greater if 80% efficacy), rather than degree type, or if vaccination also halves shedding in breakthrough infections (28–75% greater impact if 50% efficacy but only 5–17% greater if 80% efficacy). Including one-off catch-up vaccination for 10- to 14-year-olds only increases the impact on HSV-2, GUD and HIV incidence by 2–12% after 40 years but would avert 18% more HSV-2 infections and 30% more HIV infections and GUD days over 2020–2060. This also increases the short-term impact by 2040 (Supplementary Fig. S9).

### Therapeutic vaccine impact

#### Main scenarios

**Fig. 5:**
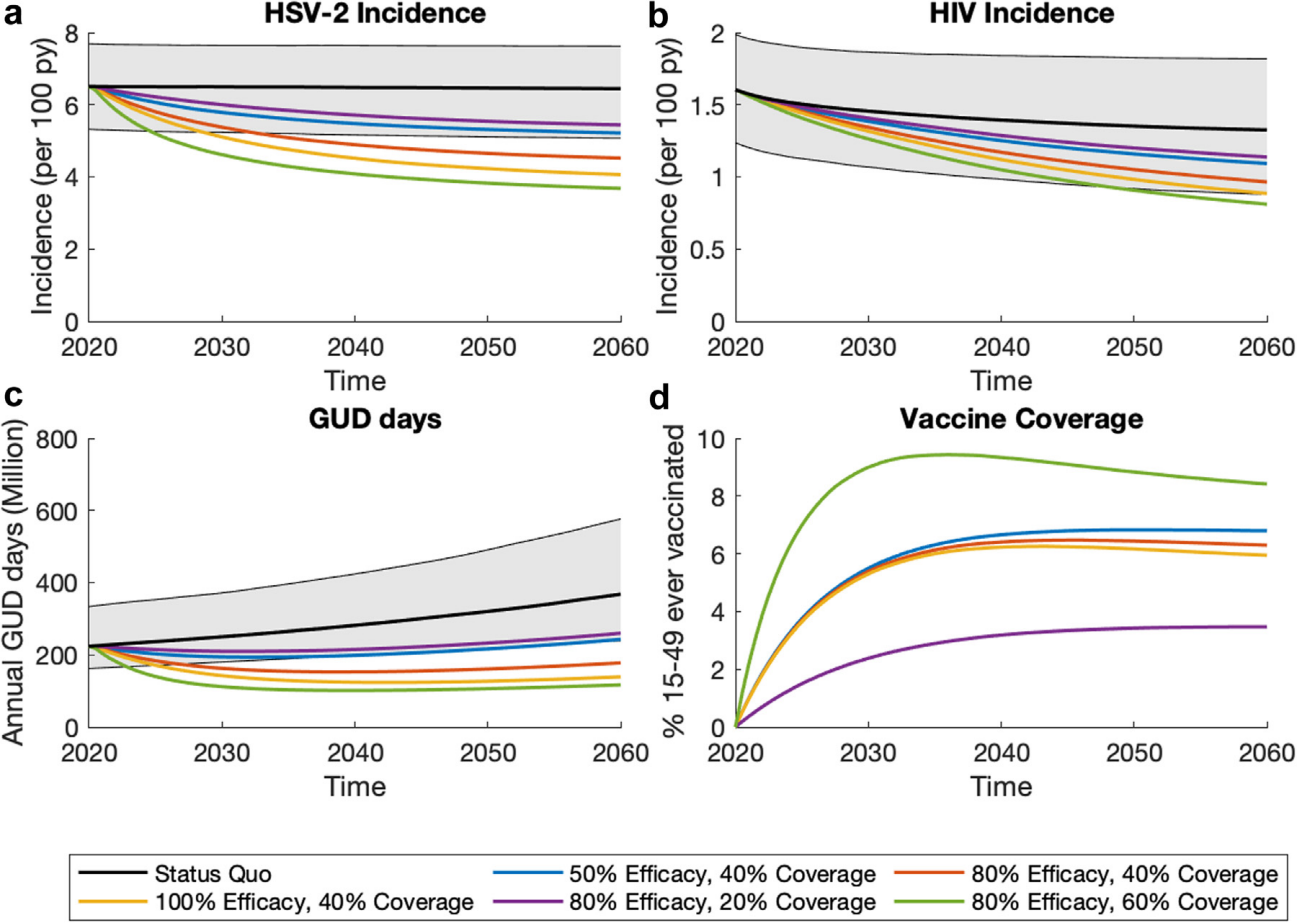
Model projections of impact of a therapeutic vaccine which has lifelong protection on (a) HSV-2 incidence among 15- to 49-year-olds; (b) HIV incidence among 15- to 49-year-olds; (c) annual number of days with GUD; (d) the proportion of 5- to 49-year-olds that have ever been vaccinated. Projections show median projections for vaccinating symptomatic individuals each year with a therapeutic vaccine which has lifelong protection and reduces days with asymptomatic or symptomatic shedding by the stated percentages given as the efficacy. Black lines and grey shading area show the median and 95% CrI for the status quo scenario.

**Fig. 6:**
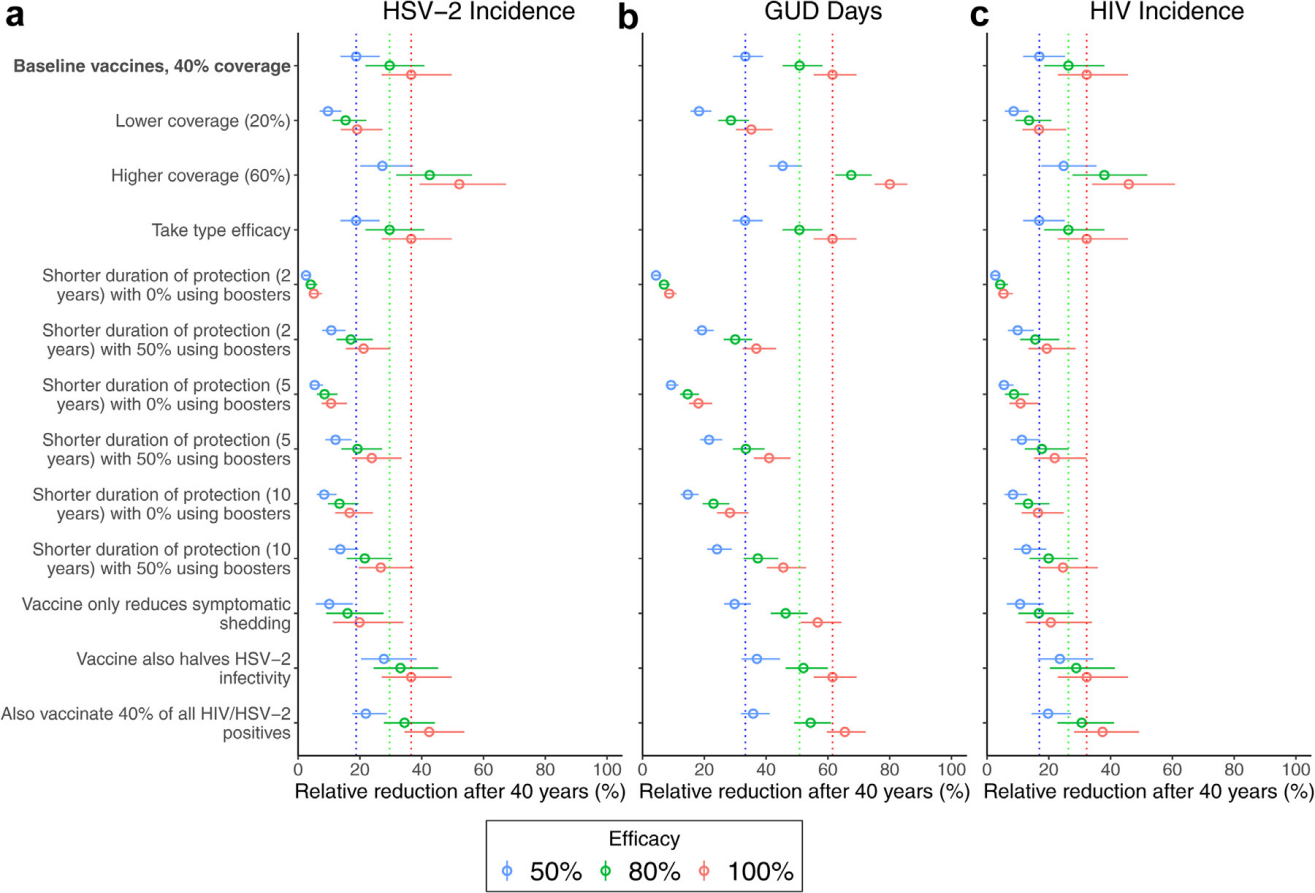
Sensitivity analyses for the 40-year impact of therapeutic vaccination on (a) HSV-2 incidence; (b) annual GUD days; (c) HIV incidence. Figures show how the impact of a therapeutic vaccine would differ in each sensitivity analysis compared to the baseline vaccination scenarios (vertical dashed lines for different vaccine efficacies): vaccinating HSV-2-infected symptomatic individuals with a therapeutic vaccine to a coverage of 40% after 40 years which has lifelong protection and reduces asymptomatic and symptomatic shedding with 50% (blue), 80% (green) or 100% (red) degree type efficacy. Impact measured as relative reduction by 2060 compared to status quo in 2060. Circles show median projections with error bars showing 95% credibility intervals.

Therapeutic vaccines are projected to have a smaller but still substantial impact on HSV-2 and HIV incidence, and could considerably reduce time with GUD (Figs. 5 and 6). For example, achieving 40% coverage among symptomatic individuals with a 50% (degree type) efficacy vaccine that provides lifelong protection could reduce HSV-2 incidence, GUD days and HIV incidence by 18.8% (95% CrI: 13.7–26.4), 33.3% (95% CrI: 29.3–39.0) and 16.9% (95% CrI: 11.7–25.3), respectively, after 40 years. Much of this impact is achieved after 20 years, although impact on HIV incidence takes longer to accrue, with only a 9.7% (95% CrI: 6.7–14.7) reduction after 20 years.

The impact of vaccination increases with efficacy and coverage (Figs. 5 and 6). With 80% efficacy and 60% coverage, HSV-2 incidence, GUD days and HIV incidence could reduce by 42.6% (95% CrI: 31.8–56.3), 67.6% (95% CrI: 62.4–74.2) and 38.0% (95% CrI: 27.7–51.9), respectively, after 40 years. Across all efficacies and coverages, HIV and HSV-2 incidence would be reduced more (by 16.6–26.4%) among women than men (Supplementary Table S13). Fig. 4 shows that for 40% coverage, the NNV to avert one HSV-2 infection, one person-year of GUD, and one HIV infection over 2020–2060 is between 0.8–1.9 (range of medians across different efficacy scenarios), 0.6–1.2, and 2.6–5.5, respectively. These NNV to avert one HSV-2 infection, GUD person-year and HIV infection are 2.3–3.7, 4.9–16.1 and 3.4–5.3 times lower for therapeutic vaccines than for prophylactic vaccines.

#### Sensitivity analyses

Fig. 6 and Supplementary Fig. S11 show that the impact of therapeutic vaccines is highly sensitive to the duration of protection and proportion that would regularly receive boosters. For example, without boosters, the 40-year impact on HSV-2 incidence, HIV incidence, and the time with GUD could be up to 84–86% lower if the vaccine provided 2 years of protection compared to a vaccine with lifelong protection. Conversely, the impact would still be at least a quarter less even if vaccines provided 10 years of protection and half of the vaccinated population regularly received boosters. In contrast to the prophylactic vaccine, results were insensitive to implementing efficacy as take type rather than degree type.

If therapeutic vaccines only reduced symptomatic shedding rather than all shedding, the 40-year impact on HSV-2 and HIV incidence would be lower (up to 46% and 36%, respectively) as would the impact on annual GUD days but to a lesser extent (up to 10%). If, however, vaccines reduced all shedding then additional impact could be achieved through also vaccinating 40% of all PLWH, particularly on reducing HSV-2 incidence (additional 17%) and HIV incidence (additional 15%). Finally, vaccination could achieve greater impact on HSV-2 incidence (up to 47%) and HIV incidence (up to 38%) if vaccines also halved HSV-2 infectivity (i.e., halved viral load rather than just reducing days with shedding) – particularly at lower efficacies against shedding.

## Discussion

Our analyses suggest that even low efficacy (50%) prophylactic HSV-2 vaccines with moderate uptake (60% - similar to the achieved uptake for HPV vaccine^29^) could reduce HSV-2 incidence by a third within 20 years in South Africa. Greater impact can be obtained with higher efficacy and uptake, with >80% reductions in HSV-2 incidence possible after 40 years with a vaccine with 100% efficacy and 60% uptake or a vaccine with 80% efficacy and 80% uptake. Vaccination can also have important benefits on HIV, with up to a two-third reduction in incidence possible within 40 years. Our results show that the impact of a therapeutic vaccine is generally smaller but nevertheless still substantial and a much greater impact per vaccination.

### Strengths and limitations

Our study shows that the public health benefit of an HSV-2 vaccine on HSV-2 and HIV could be substantial. There are residual uncertainties around HIV-HSV-2 cofactor effects which may have inflated our vaccine impact predictions. The effect of HSV-2 infection on HIV acquisition has been the subject of systematic reviews,^3^ but the effect on HIV transmission is not as well characterised, with some evidence coming from indirect sources.^5^ In all interactions, there is the possibility of residual confounding.^30^ To address this, we incorporated uncertainty in priors for all cofactors and included a lower bound of 1 (i.e., no effect) in cases of particular uncertainty. Our estimated IRRs of HIV incidence by HSV-2 status were comparable, albeit slightly higher, than pooled empirical estimates.^3^ Interestingly, we still estimated considerable contribution of HSV-2 to HIV transmission (tPAF: 66.3%; 95% CrI: 60.3–70.7) and potential impact of vaccination (Supplementary Materials Section 6) when only using model runs with IRRs falling within the 95% confidence interval of empirical estimates. Although our results show that the predicted impact on HIV is likely to considerably strengthen the public health value of a HSV vaccine, it is unlikely that HIV outcomes will be included in initial HSV vaccine trials and as an initial HSV vaccine indication.^7^ Effects on HSV-1 and neonatal herpes were beyond the scope of this study but would also add further value.

Our model projections are based on hypothetical scenarios, particularly for therapeutic vaccine efficacy which is informed only by early-stage trial results. We modelled lower vaccine efficacies alongside more speculative higher efficacies. It is possible however that even our conservative assumptions are overly optimistic. We assumed in baseline analyses for therapeutic vaccination that a linear reduction in shedding days leads to a linear reduction in HSV-2 transmission. There may be a more complex, non-linear relationship with transmission, e.g., as a consequence of the vaccine reducing both the frequency of shedding and the amount of virus released.^31^ An additional effect for the vaccine on infectivity (i.e., viral load) was explored in sensitivity analyses. Our baseline analyses also assumed lifetime protection, with our sensitivity analyses showing shorter durations of protection result in a large reduction in impact.

There was also uncertainty in model structure and parameters, and differences in how some behaviours were reported over time (e.g., condom use). The use of a Bayesian framework for model calibration, which accounts for these uncertainties, together with cross-validation to HSV-2 and HIV incidence and prevalence data, increases the confidence in our model predictions. Our model was also not stratified by age, which may have led to over-estimation of vaccine impact.

Our modelling study also has several strengths. It makes use of rich data sources to inform model parameterisation and calibration, particularly a recent review of HSV-2 and GUD natural history.^1^ We used these data to conceptualise and parameterise a novel way of capturing HSV-2 dynamics, with three groups corresponding to high, low and no GUD and associated shedding. Through this, we were able to account for heterogeneity in transmission as a function of shedding and symptoms, as well as model more realistically how a therapeutic vaccine may be deployed (i.e., to those with frequent symptoms). Our study adds greatly to the sparse literature on impact of vaccines in low- and middle-income countries, therapeutic vaccines, effect of HIV-HSV-2 cofactors on vaccine impact on HIV, and vaccine impact on GUD.^9^

### Comparisons to existing literature

Our estimates of the contribution (tPAF = 70.2%) of HSV-2 infection to HIV transmission over 10 years are higher than our previous estimates of 52.3% (95% Uncertainty Interval: 42.1–66.4) for the WHO African Region.^5^ They are also higher than other estimates for African settings, which range between 11.9 and 62.7% for individual settings.^32^^,^^33^ All these estimates assumed that HSV-2 infection increased both HIV acquisition risk and transmission risk, but by differing amounts and only measured short-term tPAFs (instantaneously or over 2 years). Our estimates of a 2-year tPAF (i.e., over 2020–2021: 54.6%; 95% CrI 48.0–60.2) are comparable to previous estimates for Cotonou (47.9–62.7%), Kisumu (37.5–54.1%), Ndola (36.1–62.7%), Yaoundé (37.5–58.1%) and Rakai (23.4–53%).^33^^,^^34^

The most comparable modelling paper for the impact of prophylactic vaccination is that of Freeman et al.,^35^ which looked at the impact on HSV-2 and HIV incidence, but not on GUD, in an age-, sex- and risk-structured population for settings in Kenya and Benin. Predicted impacts were similar and all patterns consistent with our model. Although our predicted impact on HIV after 20 years was somewhat lower, this likely reflects our earlier modelled age at vaccination (9-year-olds vs 14-year-olds) which results in a 5-year delay before achieving impact in our model. For similar reasons, our projections were also more sensitive to changes in the duration of protection.

The only models that have considered the effect of therapeutic vaccination in a specified setting have been for the USA.^36^^,^^37^ Our findings confirm those of Schwartz et al.^36^ and Ayoub et al.^37^ in predicting that the number of HSV-2 infections averted per vaccination would be greatest for a therapeutic vaccine. Our model adds to these studies by considering the impact of therapeutic vaccination on HIV and days with GUD.

In pivotal clinical trials, suppressive therapy against HSV-2 did not reduce HIV acquisition or transmission.^38^^,^^39^ One explanation for the lack of impact was that subclinical HSV-2 shedding, and associated infiltration of inflammatory cells that could be targets for HIV entry, were not sufficiently suppressed.^6^ Our analyses demonstrated much lower impact on HSV-2 and HIV incidence if a therapeutic vaccine does not suppress asymptomatic shedding. The immune cell responses generated in the genital mucosa and HSV-2 shedding following therapeutic vaccination should be carefully explored to better understand the potential impact on HIV incidence.^6^

### Impact and future directions

Prophylactic and therapeutic vaccines offer two complementary approaches for reducing the large burden of HSV-2 infection and disease both in South Africa and globally. Our results suggest that in high HIV prevalence populations such as South Africa, both vaccines could be important additional tools for controlling HIV. Modelling in settings with different HSV-2/HIV epidemics, and economic evaluations to determine the likely cost-effectiveness of any vaccination programme, are now needed to build upon our analyses of the public health impact of an HSV-2 vaccine. Our model projections suggest that HSV-2 vaccines could have important impacts as well as providing crucial insights into the efficacies and durations of protection needed to have large population-level impact. Research is also required to determine vaccine acceptability, which may differ between vaccine types and their target populations, to ensure developed vaccines will be able to be delivered at the required scale to achieve population impact.

## Contributors

K.J.L. and M.-C.B. conceived and with the co-authors developed the idea of the study which was commissioned by S.G. J.S. and R.S. co-developed and parameterised the HSV-2 component of the model, with input from K.J.L., P.V., K.M.E.T., M.-C.B., S.G. and P.M. K.J.L. performed the additional data review, synthesising data on the natural history of HSV-2 and the epidemiology of HSV-2 in South Africa. J.C., S.S. and S.B. contributed data used in model parameterisation and calibration. J.S. and K.J.L. co-wrote the manuscript and accessed and verified the data reported in the study. J.S., K.J.L., R.S., K.M.E.T., R.H., J.C., S.B., S.S., P.M., S.G., M.-C.B., and P.V. contributed to the direction of the study, advised on the model structure, gave input on the results, and reviewed manuscript drafts. All authors read and approved the final version of the manuscript.

## Data sharing statement

Model code will be made available following publication. The code will be shared with researchers who provide a methodologically sound proposal approved by J.S. and P.V. Proposals should be directed to jack.stone@bristol.ac.uk and peter.vickerman@bristol.ac.uk; requesters will need to sign a data access agreement.

## Declaration of interests

K.J.L. has received funding from GSK to model the impact and cost-effectiveness of an adolescent gonorrhoea vaccine. KMET reports that since the completion of this work she has changed position and has received payment to her current employer (Aquarius Population Health) for expert consultation in related projects including participation in an expert advisory board for GSK on gonorrhoea.
