# Supplementary material for: The population impact of herpes simplex virus type 2 (HSV-2) vaccination on the incidence of HSV-2, HIV and genital ulcer disease in South Africa: a mathematical modelling study
Source: eBioMedicine. 2023 Mar 16;90:104530. doi: 10.1016/j.ebiom.2023.104530 (PMC10034427; doi:10.1016/j.ebiom.2023.104530)
Supplement: Appendix [file mmc1.docx]

**Supplementary Materials to: The population impact of herpes simplex virus type 2 (HSV-2) vaccination on the incidence of HSV-2, HIV and genital ulcer disease in South Africa: a mathematical modelling study**

[1. Model Equations 2](#_Toc123914792)

[2. Model Schematics 11](#_Toc123914793)

[3. Further details on HSV-2 Stratification 14](#_Toc123914794)

[Supplementary Table 1. Prior values for parameters related to asymptomatic and symptomatic shedding among HSV-2-infected individuals. 15](#_Toc123914795)

[4. Model parameterisation 17](#_Toc123914796)

[Supplementary Table 2. Details of surveys used to parameterise and calibrate the HIV transmission model. 17](#_Toc123914797)

[Supplementary Table 3. Demographic, sexual and behavioural parameters of female sex workers and clients. 22](#_Toc123914798)

[Supplementary Table 4. Demographic, sexual and behavioural parameters of low risk males and females. 32](#_Toc123914799)

[Supplementary Table 5. Demographic, sexual and behavioural parameters of men who have sex with men. 36](#_Toc123914800)

[Supplementary Table 6. HIV epidemiological parameters 42](#_Toc123914801)

[Condom use assumptions 44](#_Toc123914802)

[Male circumcision coverage assumptions 47](#_Toc123914803)

[5. Model Calibration 48](#_Toc123914804)

[6. Supplementary Results 49](#_Toc123914805)

[7. References 65](#_Toc123914806)

# Model Equations

The modelled population defined as $X_{i,s,b,v}^{r}$, with

r denoting risk groups: low risk females (r=0), low-risk males (r=1), FSW (r=2), clients of FSW (r=3), young MSM (r=4), old MSM (r=5).

i denoting HIV status: susceptible (i=0), acute infection (i=1), chronic infection (i=2), Pre-AIDS (i=3), AIDS (i=4)

s denoting HSV-2 status: susceptible (s=0), high % days with GUD (s=1), low % days with GUD (s=2), never GUD (s=3).

b denoting HIV treatment status: no-ART (b=0), on ART (b=1).

v denoting HSV-2 vaccination status: unvaccinated (v=0), vaccinated and protected with no waning (v=1), vaccinated and protected but with waning (v=2), ever vaccinated but without protection (v=3)

The model can be expressed as a set of ordinary differential equation

$\frac{dX_{i,s,b,v}^{r}}{dt}=E_{i,s,b,v}^{r}+Y_{i,s,b,v}^{r}+G_{i,s,b,v}^{r}+J_{i,s,b,v}^{r}+D_{i,s,b,v}^{r}+Q_{i,s,b,v}^{r}+T_{i,s,b,v}^{r}+R_{i,s,b,v}^{r}+V_{i,s,b,v}^{r}$

Where

- $E_{i,s,b,v}^{r}$represents the recruitment into the model
- $Y_{i,s,b,v}^{r}$ represents natural mortality
- $G_{i,s,b,v}^{r}$ represents ageing
- $J_{i,s,b,v}^{r}$ represents initiation and cessation of being paid / paying for sex
- $D_{i,s,b,v}^{r}$ represents HIV infection
- $Q_{i,s,b,v}^{r}$ represents HIV disease progression and mortality
- $T_{i,s,b,v}^{r}$ represents HIV treatment (ART)
- $R_{i,s,b,v}^{r}$ represents HSV-2 acquisition
- $V_{i,s,b,v}^{r}$ represents HSV-2 Therapeutic vaccination

Each of the components of the equation are described below

***Population recruitment*** $\boldsymbol{E}_{\boldsymbol{i,s,b,v}}^{\boldsymbol{r}}$

$$E_{0,0,0,0}^{0}(t)={(1-v_{p})\delta}_{f}\delta_{0,0}^{f}\theta(t)\sum_{r} \sum_{i} \sum_{s} \sum_{b} \sum_{v} {\mu_{r}X}_{i,s,b,v}^{r}$$

$$E_{2,0,0,0}^{0}(t)={(1-v_{p})\delta}_{f}\delta_{1,0}^{f}\theta(t)\sum_{r} \sum_{i} \sum_{s} \sum_{b} \sum_{v} {\mu_{r}X}_{i,s,b,v}^{r}$$

$E_{0,s,0,0}^{0}(t)=\left( 1-v_{p} \right)\delta_{f}\delta_{0,1}^{f}H_{s}\theta(t)\sum_{r} \sum_{i} \sum_{s} \sum_{b} \sum_{v} {\mu_{r}X}_{i,s,b,v}^{r}$ for s>0

$E_{2,s,0,0}^{0}(t)={\left( 1-v_{p} \right)\delta}_{f}\delta_{1,1}^{f}H_{s}\theta(t)\sum_{r} \sum_{i} \sum_{s} \sum_{b} \sum_{v} {\mu_{r}X}_{i,s,b,v}^{r}$ for s>0

$$E_{0,0,0,2}^{0}(t)=\delta_{f}(v_{p}\delta_{0,0}^{f}+v_{p}p_{v}\delta_{0,1}^{f})\theta(t)\sum_{r} \sum_{i} \sum_{s} \sum_{b} \sum_{v} {\mu_{r}X}_{i,s,b,v}^{r}$$

$$E_{2,0,0,2}^{0}(t)=\delta_{f}(v_{p}\delta_{1,0}^{f}+v_{p}p_{v}\delta_{1,1}^{f})\theta(t)\sum_{r} \sum_{i} \sum_{s} \sum_{b} \sum_{v} {\mu_{r}X}_{i,s,b,v}^{r}$$

$E_{0,s,0,2}^{0}(t)=\delta_{f}{v_{p}(1-p_{v})\delta}_{0,1}^{f}H_{s}\theta(t)\sum_{r} \sum_{i} \sum_{s} \sum_{b} \sum_{v} {\mu_{r}X}_{i,s,b,v}^{r}$ for s>0

$E_{2,s,0,2}^{0}(t)=\delta_{f}v_{p}(1-p_{v})\delta_{1,1}^{f}H_{s}\theta(t)\sum_{r} \sum_{i} \sum_{s} \sum_{b} \sum_{v} {\mu_{r}X}_{i,s,b,v}^{r}$ for s>0

$$E_{0,0,0,0}^{1}(t)={(1-v_{p})(1-\delta_{MSM})(1-\delta}_{f})\delta_{0,0}^{m}\theta(t)\sum_{r} \sum_{i} \sum_{s} \sum_{b} \sum_{v} {\mu_{r}X}_{i,s,b,v}^{r}$$

$$E_{2,0,0,0}^{1}(t)={(1-v_{p})(1-\delta_{MSM})(1-\delta}_{f})\delta_{1,0}^{m}\theta(t)\sum_{r} \sum_{i} \sum_{s} \sum_{b} \sum_{v} {\mu_{r}X}_{i,s,b,v}^{r}$$

$E_{0,s,0,0}^{1}(t)={(1-v_{p})(1-\delta_{MSM})(1-\delta}_{f})\delta_{0,1}^{m}H_{s}\theta(t)\sum_{r} \sum_{i} \sum_{s} \sum_{b} \sum_{v} {\mu_{r}X}_{i,s,b,v}^{r}$ for s>0

$E_{2,s,0,0}^{1}(t)={(1-v_{p})(1-\delta_{MSM})(1-\delta}_{f})\delta_{1,1}^{m}H_{s}\theta(t)\sum_{r} \sum_{i} \sum_{s} \sum_{b} \sum_{v} {\mu_{r}X}_{i,s,b,v}^{r}$ for s>0

$$E_{0,0,0,2}^{1}(t)={(1-\delta_{MSM})(1-\delta}_{f})(v_{p}\delta_{0,0}^{m}+v_{p}p_{v}\delta_{0,1}^{m})\theta(t)\sum_{r} \sum_{i} \sum_{s} \sum_{b} \sum_{v} {\mu_{r}X}_{i,s,b,v}^{r}$$

$$E_{2,0,0,2}^{1}(t)={(1-\delta_{MSM})(1-\delta}_{f})(v_{p}\delta_{1,0}^{m}+v_{p}p_{v}\delta_{1,1}^{m})\theta(t)\sum_{r} \sum_{i} \sum_{s} \sum_{b} \sum_{v} {\mu_{r}X}_{i,s,b,v}^{r}$$

$E_{0,s,0,2}^{1}(t)={(1-\delta_{MSM})(1-\delta}_{f})v_{p}(1-p_{v})\delta_{0,1}^{m}H_{s}\theta(t)\sum_{r} \sum_{i} \sum_{s} \sum_{b} \sum_{v} {\mu_{r}X}_{i,s,b,v}^{r}$ for s>0

$E_{2,s,0,2}^{1}(t)={(1-\delta_{MSM})(1-\delta}_{f})v_{p}(1-p_{v})\delta_{1,1}^{m}H_{s}\theta(t)\sum_{r} \sum_{i} \sum_{s} \sum_{b} \sum_{v} {\mu_{r}X}_{i,s,b,v}^{r}$ for s>0

$$E_{0,0,0,0}^{4}(t)=(1-v_{p}){\delta_{MSM}(1-\delta}_{f})\delta_{0,0}^{m}\theta(t)\sum_{r} \sum_{i} \sum_{s} \sum_{b} \sum_{v} {\mu_{r}X}_{i,s,b,v}^{r}$$

$$E_{2,0,0,0}^{4}(t)=(1-v_{p}){\delta_{MSM}(1-\delta}_{f})\delta_{1,0}^{m}\theta(t)\sum_{r} \sum_{i} \sum_{s} \sum_{b} \sum_{v} {\mu_{r}X}_{i,s,b,v}^{r}$$

$E_{0,s,0,0}^{4}(t)={{(1-v_{p})\delta}_{MSM}(1-\delta}_{f})\delta_{0,1}^{m}H_{s}\theta(t)\sum_{r} \sum_{i} \sum_{s} \sum_{b} \sum_{v} {\mu_{r}X}_{i,s,b,v}^{r}$ for s>0

$E_{2,s,0,0}^{4}(t)={{(1-v_{p})\delta}_{MSM}(1-\delta}_{f})\delta_{1,1}^{m}H_{s}\theta(t)\sum_{r} \sum_{i} \sum_{s} \sum_{b} \sum_{v} {\mu_{r}X}_{i,s,b,v}^{r}$ for s>0

$$E_{0,0,0,2}^{4}(t)={\delta_{MSM}(1-\delta}_{f})(v_{p}\delta_{0,0}^{m}+v_{p}p_{v}\delta_{0,1}^{m})\theta(t)\sum_{r} \sum_{i} \sum_{s} \sum_{b} \sum_{v} {\mu_{r}X}_{i,s,b,v}^{r}$$

$$E_{2,0,0,2}^{4}(t)={\delta_{MSM}(1-\delta}_{f})(v_{p}\delta_{1,0}^{m}+v_{p}p_{v}\delta_{1,1}^{m})(t)\sum_{r} \sum_{i} \sum_{s} \sum_{b} \sum_{v} {\mu_{r}X}_{i,s,b,v}^{r}$$

$E_{0,s,0,2}^{4}(t)={\delta_{MSM}(1-\delta}_{f}){v_{p}(1-p_{v})\delta}_{0,1}^{m}H_{s}\theta(t)\sum_{r} \sum_{i} \sum_{s} \sum_{b} \sum_{v} {\mu_{r}X}_{i,s,b,v}^{r}$ for s>0

$E_{2,s,0,2}^{4}(t)={\delta_{MSM}(1-\delta}_{f})v_{p}(1-p_{v})\delta_{1,1}^{m}H_{s}\theta(t)\sum_{r} \sum_{i} \sum_{s} \sum_{b} \sum_{v} {\mu_{r}X}_{i,s,b,v}^{r}$ for s>0

$E_{i,s,b,v}^{r}=0$ otherwise

Where

- $\mu_{r}$ is the non-HIV rate of mortality
- $\theta(t)$ is the population growth rate.
- $H_{S}$ is the proportion of new HSV-2 infections that are allocated to each of the HSV-2 infected sub groups (frequent symptomatic recurrences, infrequent symptomatic recurrences, or no symptomatic recurrences)
- $\delta_{1,1}^{f}$ is the proportion of females entering the model who are HIV positive and HSV-2 positive
- $\delta_{1,0}^{f}$ is the proportion of females entering the model who are HIV positive and HSV-2 negative
- $\delta_{0,1}^{f}$ is the proportion of females entering the model who are HIV negative and HSV-2 positive
- $\delta_{0,0}^{f}$ is the proportion of females entering the model who are HIV negative and HSV-2 negative
- $\delta_{1,1}^{m}$ is the proportion of males entering the model who are HIV positive and HSV-2 positive
- $\delta_{1,0}^{m}$ is the proportion of males entering the model who are HIV positive and HSV-2 negative
- $\delta_{0,1}^{m}$ is the proportion of males entering the model who are HIV negative and HSV-2 positive
- $\delta_{0,0}^{m}$ is the proportion of males entering the model who are HIV negative and HSV-2 negative
- $\delta_{MSM}$ is the proportion of males entering the model who are MSM
- $\delta_{f}$ is the proportion of individuals entering the model who are female
- $p_{v}$ is the uptake of prophylactic vaccination.
- $p_{v}$ is a factor reduction in the proportion of individuals entering the model as HSV-2 infected if vaccinated.

***Non-HIV mortality*** $\boldsymbol{Y}_{\boldsymbol{i,s,b,v}}^{\boldsymbol{r}}$

$Y_{i,s,b,v}^{r}=-\mu_{r}X_{i,s,b,v}^{r}$ ,

where $\mu_{r}$ are non-HIV mortality rates, defined for females (r=0,2) and males (r=1,3,4,5) separately.

***Population ageing*** $\boldsymbol{G}_{\boldsymbol{i,s,b,v}}^{\boldsymbol{r}}$

$$G_{i,s,b,v}^{4}=-{\frac{1}{15}X}_{i,s,b,v}^{4}$$

$$G_{i,s,b,v}^{5}={\frac{1}{15}X}_{i,s,b,v}^{4}-{\frac{1}{20}X}_{i,s,b,v}^{5}$$

$$G_{i,s,b,v}^{r}=-\frac{1}{35}G_{i,s,b,v}^{r} if r<4$$

***Initiation and cessation of paying / being paid for sex*** $\boldsymbol{J}_{\boldsymbol{i,s,b,v}}^{\boldsymbol{r}}$

$J_{i,s,b,v}^{0}={-\gamma}_{0}X_{i,s,b,v}^{0}+\gamma_{2}X_{i,s,b,v}^{2}$

$J_{i,s,b,v}^{2}=\gamma_{0}X_{i,s,b,v}^{0}-\gamma_{2}X_{i,s,b,v}^{2}$

$J_{i,s,b,v}^{1}={-\gamma}_{1}X_{i,s,b,v}^{1}+\gamma_{3}X_{i,s,b,v}^{3}$

$J_{i,s,b,v}^{3}=\gamma_{1}X_{i,s,b,v}^{1}-\gamma_{3}X_{i,s,b,v}^{3}$

$J_{i,s,b,v}^{r}=0 if r=4,5$

Where

- $\gamma_{0}$ denotes the rate at which low-risk females initiate selling sex
- $\gamma_{1}$ denotes the rate at which low-risk males initiate buying sex
- $\gamma_{2}$ denotes the rate at which low-risk females cease selling sex
- $\gamma_{3}$ denotes the rate at which low-risk males cease buying sex

***HIV infection*** $\boldsymbol{D}_{\boldsymbol{i,s,b,v}}^{\boldsymbol{r}}$

HIV infection $D_{i,s,b,v}^{r}$ is derived from the rate (force) of HIV infection $\Lambda_{r,s,v}^{h}$ calculated among HIV-uninfected individuals.

$D_{i,s,b,v}^{r}=0$ if $i>1$

$D_{0,s,b,v}^{r}={-\Lambda}_{r,s,v}^{h}X_{0,s,b,v}^{r}$

$$D_{1,s,b,v}^{r}=\Lambda_{r,s,v}^{h}X_{0,s,b,v}^{r}$$

*Assumptions on the effect of HSV-2 infection and vaccination on HIV acquisition/transmission*

The per-act risk of HIV acquisition and transmission are increased among individuals with HSV infection, by an overall factor $RR_{s,b,v}^{aHIV|HSV}$ and $RR_{i,s,b,v}^{tHIV|HSV}$, respectively. These increases are given by:

$$R{R_{\beta}}_{0,s,0,v}^{aHIV|HSV}={RR_{\beta}}_{s}^{aHIV|HSV}\left( 1-\hat{G}_{0s,b,v} \right)+{RR_{\beta}}_{s}^{aHIV|GUD}\hat{G}_{0,s,b,v}$$

$$R{R_{\beta}}_{i,s,b,v}^{tHIV|HSV}={RR_{\beta}}_{s}^{tHIV|HSV}\left( 1-\hat{G}_{i,s,b,v} \right)+{RR_{\beta}}_{s}^{tHIV|GUD}\hat{G}_{i,s,b,v}$$

and are a combination of:

- An increase in the per-act risk of HIV acquisition and transmission due to HSV-2 infection, by ${RR_{\beta}}_{s}^{aHIV|HSV}$ and ${RR_{\beta}}_{s}^{tHIV|HSV}$
- A specific increase in the per-act risk of HIV acquisition and transmission during GUD, by ${RR_{\beta}}_{s}^{aHIV|GUD}$ and ${RR_{\beta}}_{s}^{tHIV|GUD}$
- The proportion of days an HSV-2 individual has GUD ($\hat{G}_{i,s,b,v})$– which depends on their HIV ($i$), HSV-2 ($s$), ART ($b$) and vaccination ($v$) status.

We model indirect effects of HSV-2 vaccination on the per-act risk of HIV acquisition and transmission by reducing the % days with GUD.

*Force of HIV infection*

Let $N_{r}$ be the total population for each group of risk $r.$ The probability of mixing with different population groups, given by risk group $r$ (low risk females (r=0), low risk males (r=1),FSW (r=2), clients of FSW (r=3), young MSM (r=4), old MSM (r=5)), for partnership type h, is $\rho_{r}^{h}$ given by:

| Probability of mixing with **low risk females** for heterosexual main and casual partnerships (h=0,1) | $\rho_{0}^{h}=\frac{n_{0}^{h}N_{0}}{n_{0}^{h}N_{0}+n_{2}^{h}N_{2}}$ |
| --- | --- |
| Probability of mixing with **FSWs** for heterosexual main and casual partnerships (h=0,1) | $\rho_{2}^{h}=\frac{n_{2}^{h}N_{2}}{n_{0}^{h}N_{0}+n_{2}^{h}N_{2}}$ |
| Probability of mixing with **FSWs** for commercial partnerships (h=2) | $\rho_{2}^{2}=1$ |
| Probability of mixing with **lower risk males** for heterosexual main and casual partnerships (h=0,1) | $\rho_{1}^{h}=\frac{n_{1}^{h}N_{1}}{n_{1}^{h}N_{1}+n_{3}^{h}N_{3}+n_{4}^{h}N_{4}+n_{5}^{h}N_{5}}$ |
| Probability of mixing with **clients of FSW of** for heterosexual main and casual partnerships (h=0,1) | $\rho_{3}^{h}=\frac{n_{3}^{h}N_{3}}{n_{1}^{h}N_{1}+n_{3}^{h}N_{3}+n_{4}^{h}N_{4}+n_{5}^{h}N_{5}}$ |
| Probability of mixing with **clients of FSW** **of** for commercial partnerships (h=2) | $\rho_{5}^{2}=1$ |
| Probability of mixing with **young MSM** for heterosexual main and casual partnerships (h=0,1) | $\rho_{4}^{h}=\frac{n_{4}^{h}N_{4}}{n_{1}^{h}N_{1}+n_{3}^{h}N_{3}+n_{4}^{h}N_{4}+n_{5}^{h}N_{5}}$ |
| Probability of mixing with **old MSM** for heterosexual main and casual partnerships (h=0,1) | $\rho_{5}^{h}=\frac{n_{5}^{h}N_{5}}{n_{1}^{h}N_{1}+n_{3}^{h}N_{3}+n_{4}^{h}N_{4}+n_{5}^{h}N_{5}}$ |
| Probability of mixing with **young** **MSM** for homosexual main and casual partnerships (h=3,4) | $\rho_{4}^{h}=\frac{n_{4}^{h}N_{4}}{n_{4}^{h}N_{4}+n_{5}^{h}N_{5}}$ |
| Probability of mixing with **old MSM** for homosexual main and casual partnerships (h=3,4) | $\rho_{5}^{h}=\frac{n_{5}^{h}N_{5}}{n_{4}^{h}N_{4}+n_{5}^{h}N_{5}}$ |

Where $\eta_{r}^{h}$ is the average number of sexual partners of type h individuals of risk group r have per year.

For females, we allow the sexual behaviour of males with females to determine who the females have sex with and how many partners they have. (A more detailed derivation can be found in (Mukandavire, Walker et al. 2018)). The HIV FOI $\Lambda_{r,s,v}^{h}$ for the low-risk female population, HSV-2 status $s$, and vaccination status $v$ due to their main ($h=0$) and casual ($h=1$) partners is as follows:

$$\Lambda_{0,s,v}^{h}=R{R_{\beta}}_{0,s,0,v}^{aHIV|HSV} \frac{p_{0}^{h}}{N_{0}}\sum_{r'=1,3,4,5} ({[\beta}_{xv}^{HIV}\left( 1-\varepsilon_{HIV} \pi_{0,r'}^{h,v} \right)\Psi_{0,r'}^{h,v}+\beta_{xa}^{HIV}\left( 1-\varepsilon_{HIV}\pi_{0,r'}^{h} \right)\Psi_{0,r'}^{h}]n_{r',}^{h}N_{r'}B_{r'})$$

Where

- $B_{r'}=\sum_{i'>0} \sum_{s'} \sum_{b'} \sum_{v'} \frac{{RR_{\beta}}_{i', s',b',v'}^{tHIV}X_{i',s',b',v'}^{r'}}{N_{r'}}$
- $R{R_{\beta}}_{i,s,b,v}^{tHIV}= R{R_{\beta}}_{i,s,b,v}^{tHIV|HSV}R{R_{\beta}}_{i}^{tHIV}R{R_{\beta}}_{b}^{tHIV}$
- $R{R_{\beta}}_{i}^{tHIV}$ is the increase in HIV transmissibility for HIV infection status $i$ compared to latent infection
- $R{R_{\beta}}_{b}^{tHIV}$ is the reduction in HIV transmissibility for ART status $b$ compared to not on ART.
- $\beta_{xv}^{HIV}$ is the per-act HIV transmission probability for receptive vaginal sex
- $\beta_{xa}^{HIV}$ is the per-act HIV transmission probability for receptive vaginal sex
- $\varepsilon_{HIV}$ is the reduction in per-act HIV transmission probability if a condom is used
- $\pi_{r,r'}^{h,v}$ and $\pi_{r,r'}^{h,a}$ are the proportions of vaginal and anal sex acts, respectively, in which a condom is used for sex acts between risk groups r and r’ for partnership type h
- $\Psi_{r,r'}^{h,v}$ and $\Psi_{r,r'}^{h,a}$ are the average number of vaginal and anal sex acts, respectively, for partnerships between risk groups r and r’.

The FOI for FSWs with HSV-2 status $s$, and vaccination status $v$ due to their main ($h=0$) and casual ($h=1$) partners is:

$$\Lambda_{2,s,v}^{h}=R{R_{\beta}}_{0,s,0,v}^{aHIV|HSV} \frac{p_{2}^{h}}{N_{2}}\sum_{r'=1,3,4,5} ({[\beta}_{xv}^{HIV}\left( 1-\varepsilon_{HIV} \pi_{2,r'}^{h,v} \right)\Psi_{2,r'}^{h,v}+\beta_{xa}^{HIV}\left( 1-\varepsilon_{HIV}\pi_{2,r'}^{h} \right)\Psi_{2,r'}^{h}]n_{r',}^{h}N_{r'}B_{r'})$$

The FOI for FSWs of HSV-2 status $s$, and vaccination status $v$ due to their commercial partners is (h=2):

$$\Lambda_{2,s,v}^{2}=R{R_{\beta}}_{0,s,0,v}^{aHIV|HSV}n_{2}^{2}({[\beta}_{xv}^{HIV}\left( 1-\varepsilon_{HIV}\pi_{2,3}^{2,v} \right)(1-p^{a})+\beta_{xa}\left( 1-\varepsilon_{HIV}\pi_{2,3}^{2,a} \right)p^{a}]B_{3})$$

Where $p^{a}$ denotes the proportion of commercial sex acts that are anal.

The FOI for the low-risk males, HSV-2 status $s$, and vaccination status $v$ due to their main $(h=0$) and casual ($h=1$) partners is

$$\Lambda_{1,s,v}^{h}={R{R_{\beta}}_{0,s,0,v}^{aHIV|HSV}\left( 1-\vartheta_{HIV}\xi\right)n}_{1}^{h}\sum_{r'=0,1} {[\beta}_{yv}^{HIV}\left( 1-\varepsilon_{HIV}\pi_{1,r'}^{h,v} \right)\Psi_{1,r',}^{h,v}+\beta_{ya}^{HIV}\left( 1-\varepsilon_{HIV}\pi_{1,r'}^{h,a} \right)\Psi_{1,r'}^{h,a}]\rho_{r'}^{h}B_{r}$$

The FOI for clients of HSV-2 status $s$, and vaccination status $v$ due to their main $(h=0$) and casual ($h=1$) partners is

$$\Lambda_{3,s,v}^{h}=R{R_{\beta}}_{0,s,0,v}^{aHIV|HSV}{\left( 1-\vartheta_{HIV}\xi\right)n}_{3}^{h}\sum_{r'=0,1} {[\beta}_{yv}^{HIV}\left( 1-\varepsilon_{HIV}\pi_{3,r'}^{h,v} \right)\Psi_{3,r'}^{h,v}+\beta_{ya}^{HIV}\left( 1-\varepsilon_{HIV}\pi_{3,r'}^{h,a} \right)\Psi_{3,r'}^{h,a}]\rho_{r',}^{h}B_{r'}$$

Where

- $\vartheta_{HIV}$ is the reduction in the risk of HIV acquisition if a male is circumcised.
- $\xi(t)$ is the proportion of men that have been circumcised.
- $\beta_{yv}^{HIV}$ is the per-act HIV transmission probability for insertive vaginal sex
- $\beta_{ya}^{HIV}$ is the per-act HIV transmission probability for insertive vaginal sex

The FOI for clients of HSV-2 status $s$, and vaccination status $v$ due to their commercial partners is (h=2):

$$\Lambda_{3,s,v}^{2}=R{R_{\beta}}_{0,s,0,v}^{aHIV|HSV}n_{3}^{2}({[\beta}_{xv}^{HIV}\left( 1-\varepsilon_{HIV}\pi_{3,2}^{2,v} \right)(1-p^{a})+\beta_{xa}^{HIV}\left( 1-\varepsilon_{HIV}\pi_{3,2}^{2,a} \right)p^{a}] B_{2}$$

The FOI for young (r=4) or old (r=5) MSM of HSV-2 status $s$, and vaccination status $v$ due to their heterosexual main $(h=0$) and casual ($h=1$) partners is

$$\Lambda_{r,s,v}^{h}=R{R_{\beta}}_{0,s,0,v}^{aHIV|HSV}{\left( 1-\vartheta_{HIV}\xi\right)n}_{r}^{h}\sum_{r'=0,1} {[\beta}_{yv}^{HIV}\left( 1-\varepsilon_{HIV}\pi_{r,r'}^{h,v} \right)\Psi_{r,r'}^{h,v}+\beta_{ya}^{HIV}\left( 1-\varepsilon_{HIV}\pi_{r,r'}^{h,a} \right)\Psi_{r,r'}^{h,a}]\rho_{r'}^{h}B_{r'}$$

The FOI for young (r=4) or old (r=5) MSM of HSV-2 status $s$, and vaccination status $v$ due to their homosexual main $(h=3$) and casual ($h=4$) partners is

$$\Lambda_{r,s,v}^{h}=R{R_{\beta}}_{0,s,0,v}^{aHIV|HSV}\left( 1-\frac{\vartheta_{HIV}\xi}{2} \right)\frac{\left( \beta_{xa}^{HIV}+\beta_{ya}^{HIV} \right)}{2}\sum_{r^{'}=5,6} \left( 1-\varepsilon_{HIV}\pi_{r,r'}^{h,a} \right)\Psi_{r,r'}^{h,v}\rho_{r'}^{k}B_{r'}$$

***HIV disease progression and mortality*** $\boldsymbol{Q}_{\boldsymbol{i,s,b,v}}^{\boldsymbol{r,a}}$

$$Q_{0,s,b,v}^{r}=0$$

$Q_{1,s,0,v}^{r}=-\alpha_{a}X_{1,s,0,v}^{r}$

$Q_{2,s,0,v}^{r}=\alpha_{a}X_{1,s,0,v}^{r}-\alpha_{l}X_{2,s,0,v}^{r}$

$Q_{3,s,0,v}^{r}=\alpha_{l}X_{2,s,0,v}^{r}-\alpha_{p}X_{3,s,0,v}^{r}$

$Q_{4,s,0,v}^{r}=\alpha_{p}X_{3,s,0,v}^{r}-\hat{\mu}_{b}X_{4,s,0,v}^{r}$

$Q_{2,s,1,v}^{r}={Z\alpha}_{a}X_{1,s,1,v}^{r}-{Z\alpha}_{l}X_{2,s,1,v}^{r}$

$Q_{3,s,1,v}^{r}={Z\alpha}_{l}X_{2,s,1,v}^{r}-{Z\alpha}_{p}X_{3,s,1,v}^{r}$

$Q_{4,s,1,v}^{r}={Z\alpha}_{p}X_{3,s,1,v}^{r}-Z\hat{\mu}_{b}X_{4,s,1,v}^{r}$

Where

- $\alpha_{a}$ is the rate of progression from acute HIV infection to latent HIV infection.
- $\alpha_{l}$ is the rate of progression from latent HIV infection to pre-AIDS.
- $\alpha_{p}$ is the rate of progression from pre-AIDS to AIDS
- $\hat{\mu}_{b}$ is the mortality rate due to HIV when not on ART
- $Z$ is the reduction in HIV progression/mortality if on ART

***HIV treatment*** $\boldsymbol{T}_{\boldsymbol{i,s,b,v}}^{\boldsymbol{r}}$

$$T_{i,s,b,v}^{r}=0 if i=0,1$$

$$T_{i,s,0,v}^{r}=-ART_{1}^{r}X_{i,s,0,v}^{r}+ART_{0}^{r}X_{i,s,1,v}^{r} if i\geq2$$

$$T_{i,s,1,v}^{r}=ART_{1}^{r}X_{i,s,0,v}^{r}-ART_{0}^{r}X_{i,s,1,v}^{r} if i\geq2$$

Where

- $ART_{1}^{r}$denotes the ART initiation rate for each risk group
- $ART_{0}^{r}$ denotes the ART loss to care rate for each risk group

***HSV-2 infection*** $\boldsymbol{R}_{\boldsymbol{i,s,b,v}}^{\boldsymbol{r}}$

HSV-2 infection $R_{i,s,b,v}^{r}$ derives from the rate (force) of HSV-2 infection $\lambda_{r,v}^{h}$ among HSV-2 uninfected individuals.

$$R_{i,s,b,v}^{r}=0 if a=0$$

$R_{i,0,b,v}^{r}=-\lambda_{r,v}^{h}X_{i,0,b,v}^{r}$

$$R_{i,s,b,v}^{r}=\lambda_{r,v}^{h}X_{i,0,b,v}^{r}H_{s} if s>0$$

Where $H_{s}$ are the fractions of newly HSV-2 infected individuals that are allocated in the 3 different groups of HSV-2-positive individuals, which reflect different frequencies of symptomatic shedding.

**Model Assumptions**

The model assumes that:

- HSV-2 transmits during periods of asymptomatic and symptomatic (GUD) shedding
- Not every HSV-2 infected individual can have GUD, but they all have days with shedding during the year
- The per-act risk of HSV-2 transmission is higher during days with GUD than during days with asymptomatic shedding (by $R{R_{\beta}}^{HSV|GUD})$
- HIV (and ART) does not affect the per-act risk of HSV-2 transmission but there is an indirect effect of HIV (and ART) on HSV-2 transmission through affecting the proportion of days with asymptomatic shedding and GUD.
- There is no effect of an individual’s HIV (or ART) status on their risk of HSV-2 acquisition

**Force of HSV-2 infection**

The FOI $\lambda_{r,v}^{h}$ for the low-risk female population of age vaccination status $v$ due to their main ($h=0$) and casual ($h=1$) partners is as follows:

$$\lambda_{1,v}^{h}=R{R_{\beta}}_{v}^{aHSV}\frac{p_{0}^{h}}{N_{0}}\sum_{r'=1,3,4,5} ({[\beta}_{xv}^{HSV}\left( 1-\varepsilon_{HSV}\pi_{0,r'}^{h,v} \right)\Psi_{0,r'}^{h,v}+\beta_{xa}^{HSV}\left( 1-\varepsilon_{HSV}\pi_{0,r'}^{h,a} \right)\Psi_{0,r'}^{h,a}]n_{r'}^{h}N_{r'}C_{r'})$$

Where,

- $C_{r'}=\sum_{i'} \sum_{b'} \sum_{s'>0} \sum_{v'} \frac{(A_{i^{'},s^{'},b',v'}+\hat{G}_{i^{'},s^{'},b',v'}R{R_{\beta}}^{HSV|GUD})X_{i',s',b',v'}^{r'}}{N_{r'}}$
- $\beta_{xv}^{HSV}$ is the per-act HSV-2 transmission probability for receptive vaginal sex
- $\beta_{xa}^{HSV}$ is the per-act HSV-2 transmission probability for receptive anal sex
- $\varepsilon_{HSV}$ is the reduction in per-act HSV-2 transmission probability if a condom is used
- $R{R_{\beta}}_{v}^{aHSV}$ is the reduction in per-act HSV-2 acquisition probability based on vaccination status v. $R{R_{\beta}}_{v}^{aHSV}=(1-vaccine efficacy)$, if v=1,2 and $R{R_{\beta}}_{v}^{aHSV}=1$ if v=0,3.

The FOI for FSWs of vaccination status $v$ due to their main ($h=0$) and casual ($h=1$) partners is:

$$\lambda_{2,v}^{h}=R{R_{\beta}}_{v}^{aHSV}\frac{p_{2}^{h}}{N_{2}}\sum_{r'=1,3,4,5} ({[\beta}_{xv}^{HSV}\left( 1-\varepsilon_{HSV}\pi_{2,r'}^{h,v} \right)\Psi_{2,r'}^{h,v}+\beta_{xa}\left( 1-\varepsilon_{HSV}\pi_{2,r'}^{h,a} \right)\Psi_{2,r'}^{h,a}]n_{r'}^{h}N_{r'}C_{r'})$$

The FOI for FSWs of age group $a$ and vaccination status $v$ due to their commercial partners is (h=2):

$$\lambda_{2,v}^{2}=R{R_{\beta}}_{v}^{aHSV}n_{2}^{2}({[\beta}_{xv}^{HSV}\left( 1-\varepsilon_{HSV}\pi_{2,3}^{2,v} \right)(1-p^{a})+\beta_{xa}^{HSV}\left( 1-\varepsilon_{HSV}\pi_{2,3}^{2,a} \right)p^{a}]C_{3})$$

The FOI for the lower-risk males of vaccination status $v$ due to their main $(h=0$) and casual ($h=1$) partners is

$$\lambda_{1,v}^{h}={R{R_{\beta}}_{v}^{aHSV}\left( 1-\vartheta_{HSV}\xi\right)n}_{1}^{h}\sum_{r'=0,2} {[\beta}_{yv}^{HSV}\left( 1-\varepsilon_{HSV}\pi_{1,r'}^{h,v} \right)\Psi_{1,r'}^{h,v}+\beta_{ya}^{HSV}\left( 1-\varepsilon_{HSV}\pi_{1,r'}^{h,a} \right)\Psi_{1,r'}^{h,a}]\rho_{1,r'}^{h}C_{r'}$$

Where

- $\vartheta_{HSV}$ is the reduction in the risk of HSV-2 acquisition for circumcised men.
- $\beta_{yv}^{HSV}$ is the per-act HSV-2 transmission probability for insertive vaginal sex
- $\beta_{ya}^{HSV}$ is the per-act HSV-2 transmission probability for insertive anal sex

The FOI for clients of vaccination status $v$ due to their main $(h=0$) and casual ($h=1$) partners is

$$\lambda_{3,v}^{h}=R{R_{\beta}}_{v}^{aHSV}{\left( 1-\vartheta_{HSV}\xi\right)n}_{3}^{h}\sum_{r^{'}=1,2} {[\beta}_{yv}^{HSV}\left( 1-\varepsilon_{HSV}\pi_{3,r'}^{h,v} \right)\Psi_{3,r'}^{h,v}+\beta_{ya}^{HSV}\left( 1-\varepsilon_{HSV}\pi_{3,r'}^{h,a} \right)\Psi_{3,r'}^{h,a}]\rho_{r'}^{h}C_{r'}$$

The FOI for clients of age vaccination status $v$ due to their commercial partners is (h=2):

$$\lambda_{3,v}^{2}=R{R_{\beta}}_{v}^{aHSV}n_{3}^{2}({[\beta}_{xv}^{HSV}\left( 1-\varepsilon_{HSV}\pi_{3,2}^{2,v} \right)(1-p^{a})+\beta_{xa}^{HSV}\left( 1-\varepsilon_{HSV}\pi_{3,2}^{2,a} \right)p^{a}] C_{2})$$

The FOI for young (r=4) and old (r=5) MSM of vaccination status $v$ due to their heterosexual main $(h=0$) and casual ($h=1$) partners is

$$\lambda_{r,v}^{h}=R{R_{\beta}}_{v}^{aHSV}{\left( 1-\vartheta_{HIV}\xi\right)n}_{r}^{h}\sum_{r'=0,2} {[\beta}_{yv}^{HSV}\left( 1-\varepsilon_{HSV}\pi_{r,r'}^{h,v} \right)\Psi_{r,r'}^{h,v}+\beta_{ya}^{HSV}\left( 1-\varepsilon_{HSV}\pi_{r,r'}^{h,a} \right)\Psi_{r,r'}^{h,a}]\rho_{r'}^{h}C_{r'}$$

The FOI for young (r=4) and old (r=5) MSM of vaccination status $v$ due to their homosexual main $(h=3$) and casual ($h=4$) partners is

$$\lambda_{r,v}^{h}=R{R_{\beta}}_{v}^{aHSV}\left( 1-\frac{\vartheta_{HSV}\xi}{2} \right)\frac{\left( \beta_{xa}^{HSV}+\beta_{ya}^{HSV} \right)}{2}\sum_{r'} \left( 1-\varepsilon_{HSV}\pi_{r,r'}^{h,a} \right)\Psi_{r,r'}^{h,v}\rho_{r,r'}^{k}C_{r'}$$

***HSV-2 Therapeutic vaccination*** $\boldsymbol{V}_{\boldsymbol{i,s,b,v}}^{\boldsymbol{r}}$ ***and vaccine waning***

$$V_{i,s,b,0}^{r}=-\phi_{i,s}X_{i,s,b,0}^{r}$$

$$V_{i,s,b,1}^{r}={p_{b}\phi}_{i,s}X_{i,s,b,0}^{r}$$

$$V_{i,s,b,2}^{r}=(1-p_{b}){\phi_{i,s}X}_{i,s,b,0}^{r}-\omega X_{i,s,b,2}^{r}$$

$$V_{i,s,b,3}^{r}=\omega X_{i,s,b,2}^{r}$$

Where

- $\phi_{i,s}$ is the rate at which individuals are vaccinated with a therapeutic vaccine.
- $p_{b}$ is the proportion of individuals that will always receive therapeutic booster vaccines.
- $\omega$ is the rate of waning of vaccine effects. In scenarios, in which boosters are given this is also equivalent to the rate at which boosters are given to those that always receive boosters.

# Model Schematics

**
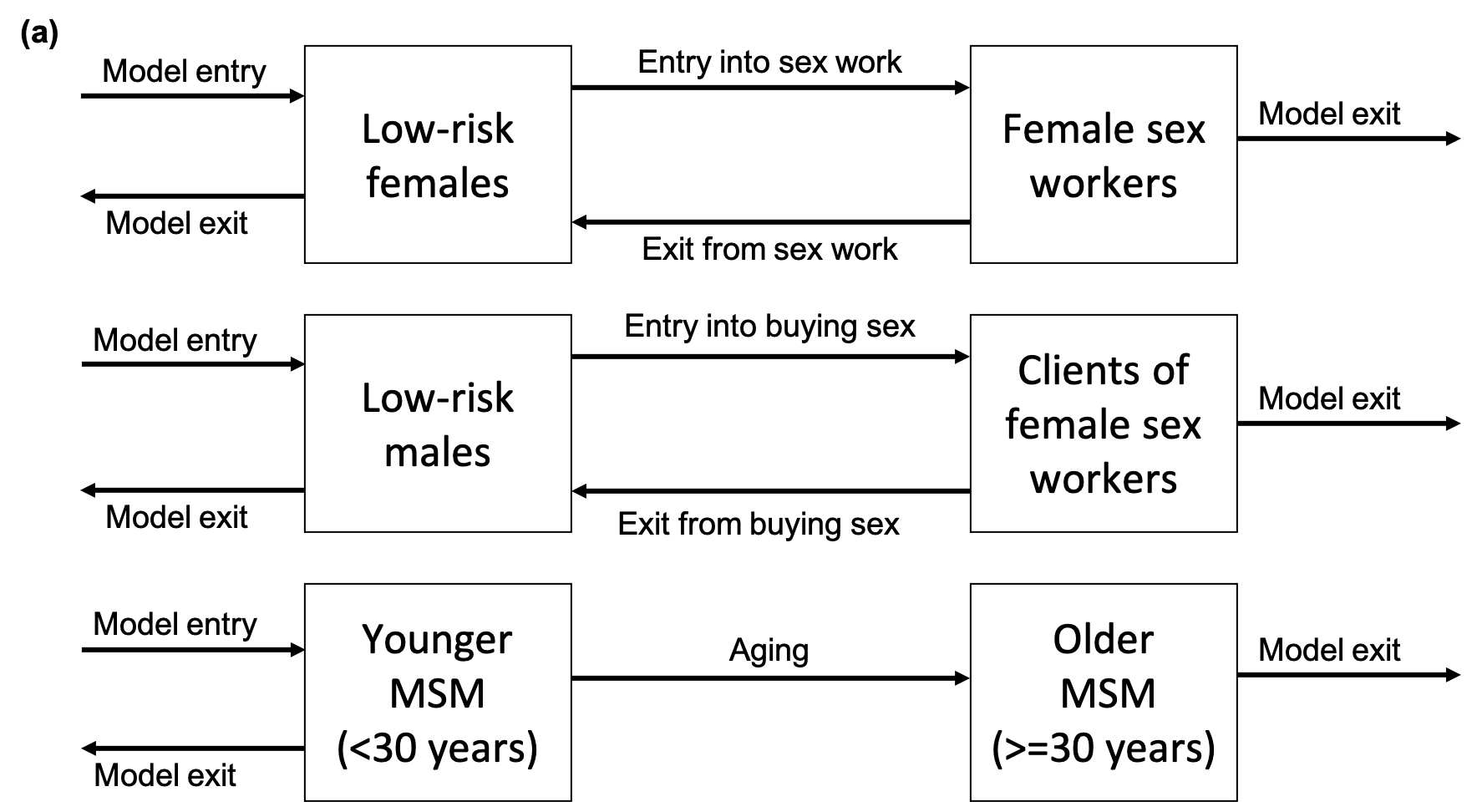
**

**Supplementary Figure 1a:** Model schematics illustrating the movement of individuals in and out of different sub-populations.

**Supplementary Figure 1b:** Model schematics illustrating the sexual interactions which can result in HIV or HSV-2 transmission among low-risk females, low-risk males, female sex workers, their clients and men who have sex with men.

**
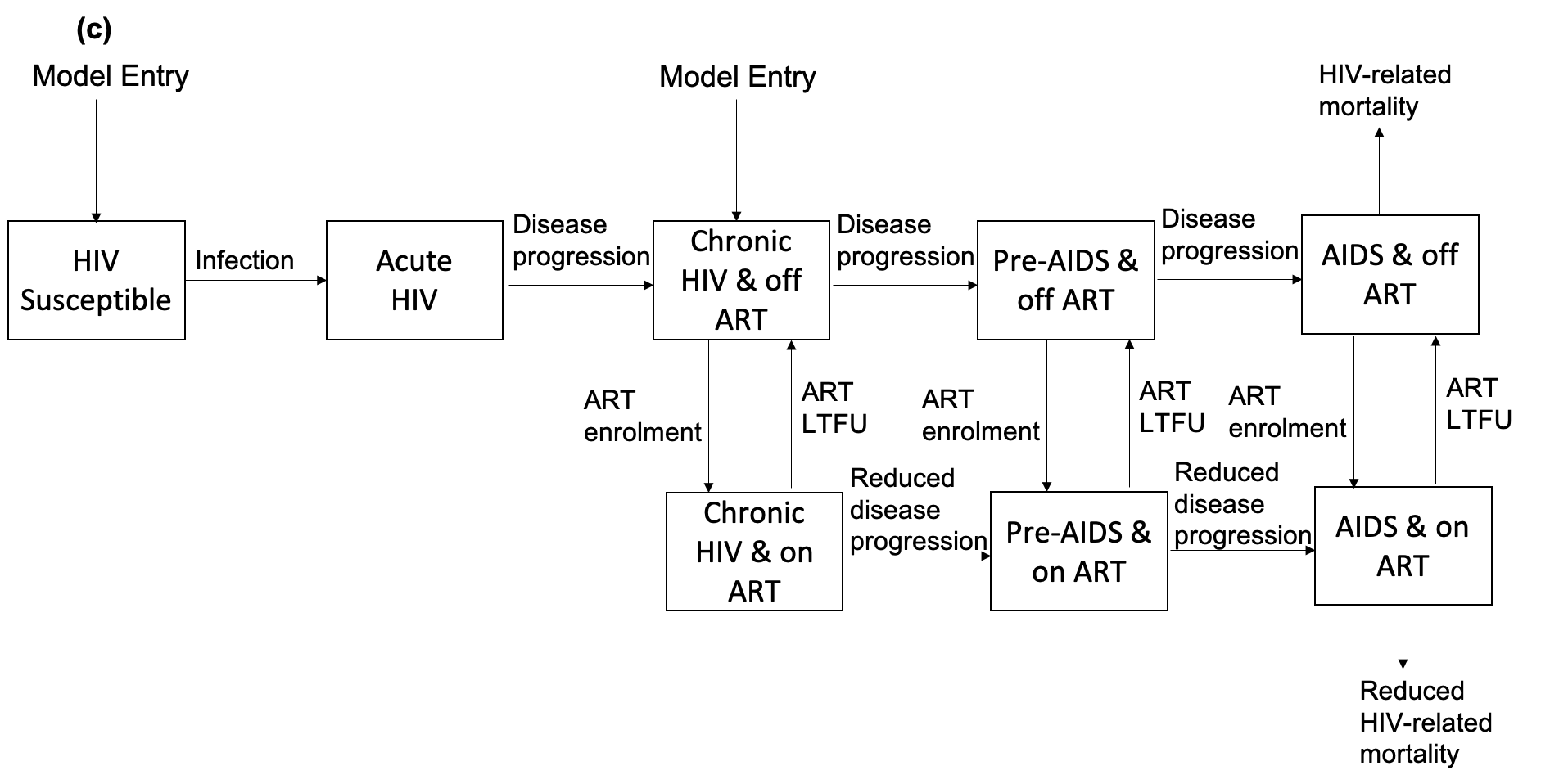
**

**Supplementary Figure 1c:** Model schematics illustrating the stratification of the population with respect to HIV infection. LTFU denotes loss to follow-up.

Waning of protection

Model entry

Model entry

Model entry

HSV-2 unvaccinated

HSV-2 vaccinated and
protected

Ever vaccinated but without protection

**Supplementary Figure 2a:** Model states relevant to prophylactic vaccination. In baseline analyses, no waning is modelled (vaccines provide lifelong protection) and there is no model entry into the ever vaccinated but without protection compartment (vaccine take is 100%).

Waning of protection

HSV-2 unvaccinated

Vaccinated and
protected, never receive boosters

Ever vaccinated but without protection

Vaccinated and
protected, always receive boosters

Vaccination

Vaccination

Vaccination

**Supplementary Figure 2b:** Model states relevant to therapeutic vaccination. In baseline analyses, all individuals enter the ‘Vaccinated and protected, always receive boosters’ compartment when vaccinated (vaccines provide lifelong protection with 100% take).

# Further details on HSV-2 Stratification

The choice of compartmentalizing the HSV-2 infected population based on the percentage of days with symptoms was motivated by the fact that a therapeutic vaccine would be more likely to be administrated to individuals with more frequent symptoms, who would also be more likely to present to care. Therapeutic vaccination could be an alternative to antiviral therapy. Currently, symptomatic individuals are more likely to be diagnosed, particularly those with a high recurrence rate: asymptomatic individuals are usually unaware they are infected(Langenberg, Benedetti et al. 1989, Brown, Benedetti et al. 1995, Langenberg, Corey et al. 1999, Wald, Zeh et al. 2000, Fife, Williams et al. 2010) (Fanfair, Zaidi et al. 2013).

The choice of compartmentalizing the model was also part-motivated by the available data, which tends to come either from clinic populations (assumed to be similar to group 1) or those with unrecognised infection (assumed to be similar to groups 2 and 3). The model assumed that the average annual number of symptomatic episodes is the same in groups 1 and 2 but the duration of each episode is longer in group 1, based on data (see **Supplementary Table 1**) from clinic populations (=group 1) and those with unrecognised infection (=groups 2 and 3). We assumed that HIV does not vary the proportions of new infections that enter each of the HSV-2 infection groups, instead varying the (asymptomatic or symptomatic) shedding rates in each, which we assumed was by the same relative amount. This is a reasonable assumption as HSV-2 infection usually precedes HIV infection among HSV-2-HIV coinfected individuals. We did not consider the effect of HIV infection on increasing HSV-2 genital viral load during shedding as it is unclear how this translates to an increase in HSV-2 transmission.

For simplicity, the somewhat longer duration and high infectivity associated with a first symptomatic episode of infection was ignored; previous modelling(Foss, Vickerman et al. 2009) suggests this has little impact if the model is fit to HSV-2 prevalences near their equilibrium values as is the case for our model. The frequency and duration of asymptomatic shedding in each infection group were parameterised based on a literature review of HSV-2 shedding studies undertaken for this study. Based on data that shows a similar percentage of days with asymptomatic shedding in HSV-2 infected individuals with and without a history of GUD if they don’t have HIV infection (see **Supplementary Table 1**), we assumed that in the absence of HIV, asymptomatic shedding occurs at the same rate in all three groups.

## Supplementary Table 1. Prior values for parameters related to asymptomatic and symptomatic shedding among HSV-2-infected individuals.

| **Parameter** | **Estimate (Prior range (min-max))** | | | **Notes/references** |
| --- | --- | --- | --- | --- |
|  | **HSV-2-infected individuals with frequent symptomatic shedding**  **(s=1)** | **HSV-2-infected individuals with infrequent symptomatic shedding (s=2)** | **HSV-2-infected individuals with no symptomatic shedding (s=3)** |  |
| Percentage of new infections in each infection group ($H_{s})$ | 12.6%  (9.6-16.3) | 24.6%  (12.3-41.3) | 62.8%  (42.4-78.1) | $H_{1}$ is based on US data (NHANES 2007-2010) for the percentage of those HSV-2 seropositive who are diagnosed: 12.6% (95%CI: 9.6-16.3)(Fanfair, Zaidi et al. 2013), which we assume are symptomatic. This refers to the percentage to which clinic data (i.e., frequent symptomatic shedding) can be applied(Looker, Johnston et al. 2020). *H*_2_ was estimated from the estimated percentage of individuals with unrecognised infection (*H*_2_ + *H*_3_) who have recurrences from a meta-analysis(Looker, Johnston et al. 2020) of 3 studies(Wald, Zeh et al. 2002, Leone, Warren et al. 2007, Tronstein, Johnston et al. 2011)_._ , and H3 is the remainder. |
| Percentage of days with asymptomatic shedding among HIV-uninfected individuals | 11.2%  (2-33) | 11.2%  (2-33) | 11.2%  (2-33) | (Wald, Zeh et al. 2000, Phipps, Saracino et al. 2011, Tronstein, Johnston et al. 2011, Phipps, Nakku-Joloba et al. 2016) Weighted mean (by sample size) of the % of days with asumptomatic shedding across available estimates is 11.2%. Range over studies is 2-33%(Looker, Johnston et al. 2020). In empirical studies, the % of days with asymptomatic shedding is similar among those who have vs those who do not have a history of symptomatic episodes(Wald, Zeh et al. 2000, Tronstein, Johnston et al. 2011). We therefore assumed the same rate of asymptomatic shedding across the three groups. |
| Average number of symptomatic episodes per year | 5.0  (4.3-5.9) | 5.0  (4.3-5.9) | 0.0 | The pooled(Looker, Johnston et al. 2020) (N=18 studies) annual number of symptomatic episodes among clinic populations is 5.0 (95%CI: 4.3-5.9)(Bryson, Dillon et al. 1985, Peacock, Kaplowitz et al. 1988, Koelle, Benedetti et al. 1992, Wald, Zeh et al. 1995, Wald, Zeh et al. 1996, Boggess, Watts et al. 1997, Benedetti, Zeh et al. 1999, Cohen, Kemeny et al. 1999, Wald, Zeh et al. 2002, Gupta, Wald et al. 2004, Handsfield, Warren et al. 2007, Fife, Warren et al. 2008, Phipps, Saracino et al. 2011, Tronstein, Johnston et al. 2011, Wald, Corey et al. 2014, Bender Ignacio, Perti et al. 2015, Bernstein, Wald et al. 2017, Franzen-Rohl, Schepis et al. 2017). The pooled(Looker, Johnston et al. 2020) (N=5 studies) annual number of symptomatic episodes among individuals with unrecognised infection is 3.4 (95%CI: 2.7-4.4)(Bryson, Dillon et al. 1993, Boggess, Watts et al. 1997, Wald, Zeh et al. 2000, Leone, Warren et al. 2007, Tronstein, Johnston et al. 2011). However, only a percentage of individuals with unrecognised infection (reflecting *H*_2_ and *H*_3_ groups) will have symptoms (group *H*_2_)(Wald, Zeh et al. 2002, Leone, Warren et al. 2007, Tronstein, Johnston et al. 2011). Fitting to this percentage and the average duration of a symptomatic episode, we found a similar average number of episodes between the groups *H*_1_ and *H*_2_. |
| Average duration of a symptomatic episode (days) | 8.5  (7.5-9.5) | 3.0  (1.6-5.7) | 0.0 | Pooled(Looker, Johnston et al. 2020) (N=13 studies) average duration of symptomatic recurrences among clinic populations is 8.5 days (95%CI: 7.9-9.5)(Corey, Nahmias et al. 1982, Douglas, Critchlow et al. 1984, Benedetti, Zeh et al. 1995, Straus, Wald et al. 1997, Wald, Zeh et al. 2000, Wald, Carrell et al. 2002, Gupta, Wald et al. 2004, Aoki, Tyring et al. 2006, de Bruyn, Vargas-Cortez et al. 2006, Mark, Corey et al. 2007, Leone, Abudalu et al. 2010, Phiri, Hoffman et al. 2010, Phipps, Saracino et al. 2011). Among a US study of individuals with unrecognised infection, the average duration of recurrent symptomatic episodes was 3.0 days (95%CI: 1.6-5.7)(Wald, Zeh et al. 2000, Looker, Johnston et al. 2020). |
| Relative risk in days of asymptomatic shedding if untreated HIV-infected vs HIV-uninfected | 2.16 (1.09-4.28) | | | No estimate was directly available, so we used a pooled estimate of the relative risk of all shedding if untreated HIV-infected vs HIV-uninfected from (Kelly and Mayaud 2019): (2.16 (95%CI: 1.09-4.28), N=3 studies). |
| Relative risk in days of symptomatic shedding if untreated HIV-infected vs HIV-uninfected | 2.78 (1.51-5.14) | | | No estimate was directly available, so we used a pooled estimate of the relative risk of symptomatic shedding if untreated HIV-infected vs HIV-uninfected from (Kelly and Mayaud 2019): (2.78 (95%CI: 1.51-5.14), N=3 studies). |
| Relative risk in days of shedding (asymptomatic or symptomatic) if treated vs untreated PLHIV | 0.56 (0.41-0.77) | | | Comparing treated PLHIV to untreated PHLIV, OR for any shedding is 0.56 (95%CI: 0.41-0.77). This reduction was applied to the relative risk described above in order to ensure that treated PLHIV always shed HSV-2 more frequently than HIV-uninfected individuals. |

# Model parameterisation

## Supplementary Table 2. Details of surveys used to parameterise and calibrate the HIV transmission model.

| Year | How sampled/recruited | Sample size | Setting | HIV prevalence % (95%CI) | Reference |
| --- | --- | --- | --- | --- | --- |
| FSW | | | | | |
| 1997/98 | Venue/street based | 295 | Hillbrow | 46.4% (40.6-52.3) | (Dunkle, Beksinska et al. 2005) |
| 1996/97 | Recruited from five truck stops | 145 | KwaZulu-Natal | 50.3% (41.2-58.7) | (Ramjee, Karim et al. 1998) |
| 2000 | Outreach workers | 247 | Hillbrow, Johannesburg | 44.9% (38.6-51.4) | (Rees, Beksinska et al. 2000) |
| 2013/14 | RDS | 764  650  766 | Johannesburg  Cape Town  Durban  Overall | 71.8% (56.5-81.2)  39.5% (30.1-49.8)  53.5% (37.5-65.6)  58.5% (56.4-60.6) | (SAHMS-FSW 2014) |
| 2014/15 | Peer educators recruited FSW who can then access health HIV and sexual reproductive health services. Retrospective analysis | 1422  408 | Johannesburg (1364 tested)  Pretoria (399 tested) | 42.2% (39.5-44.8)  52.9% (47.9-57.9) | (Slabbert, Venter et al. 2017) |
| 2014/15 | RDS | 410 | Port Elizabeth and surrounding areas | 61.5% (54.1-68.0) | (PEFSW 2015, Rao, Baral et al. 2016) |
| 2015/16 | Venue/street based | 692 | Pretoria and Inner Johannesburg | 49.2% (45.5-53.1) | (Gomez, Eakle et al. 2016, Eakle, Gomez et al. 2017) |
| 2016 | RDS | 508 | Soweto | 53.6% (47.5-59.9) | (Coetzee, Jewkes et al. 2017) |
| 2016/17 | Opportunity based sampling whilst accessing community-based HIV services for KPs | 1531 | Cape Town, Durban, Mthatha, Pietermaritzburg, Port Elizabeth | 46.6% (44.0-49.1) |  |
| Clients | | | | | |
| 2017/18 | Venue/street based (time location sampling) | 600 | Port Elizabeth | 14.0% (11.4-17.0) | Unpublish-ed data |
| 2018 | Recruited by FSW | 115 | Klerksdorp | 29.8% (21.6-39.1) |  |
| MSM | | | | | |
| Unknown | Internet-based | 824 | National | Not available | (Stephenson, de Voux et al. 2011) |
| 1988-1990 | Attendees of STD and family planning clinics | 86 | Johannesburg | 18.6% (11.0-28.4) | (Schoub, Smith et al. 1990) |
| 2003/2005 | Event/network based and internet-based | 1,045 | Gauteng, KwaZulu-Natal and Western Cape | Not available | (Sandfort, Nel et al. 2008) |
| 2016/17 | Opportunity based sampling whilst accessing community-based HIV services for KPs | 747 | Cape Town, Johannesburg, Pretoria | 42.8% (39.3-46.5) |  |
| 2015/16 | RDS | 545  525  476  359 | Johannesburg  Bloemfontein  Mafikeng  Polokwane | 43.4%  17.3%  14.6%  22.4% | (Kufa, Lane et al. 2017, Johnson, Mulongeni et al. 2018) |
| 2014/15 | Internet-based survey | 408 | National | Not available | (Hugo, Stall et al. 2016) |
| 2012/13 | RDS | 307  298 | Gert Sibande  Ehlanzeni | 28.3% (21.1–35.3)  13.7% (9.1–19.6) | (Lane, Osmand et al. 2014) |
| 2004/5 | Chain-referral and venue-based recruitment | 199 | Gauteng province | Not available | (Lane, Shade et al. 2008) |
| 2011-13 | RDS of black MSM | 480 | Pretoria | 30.1% | (Sandfort, Lane et al. 2015, Knox, Reddy et al. 2017) |
| 2014-16 | Clients of primary health care facilities | 5,796; 1,107 MSM | Johannesburg | 58.4% (55.4-61.3) | (Rees, Radebe et al. 2017) |
| 2005 | Street intercepts and snowball sampling | 37 | Cape Town, Durban, Pretoria | 35.1% (20.2-52.5) | (Parry, Petersen et al. 2008) |
| 2008 | RDS | 378 | Soweto | 13.2% (12.4-13.9) | (Lane, Raymond et al. 2011, Arnold, Struthers et al. 2013) |
| 2008 | Event/venue based. | 300 | Pretoria | Not available | (Knox, Yi et al. 2010, Knox, Reddy et al. 2013) |
| 2008 | Household survey | 73 MSM | Eastern Cape and KwaZulu-Natal provinces | 27.4% (17.6-40.0) | (Dunkle, Jewkes et al. 2013) |
| 2008 | RDS | 204  81 | Johannesburg  Durban | 49.5% (42.5-56.5)  27.5% (17.0-38.1) | (Rispel, Metcalf et al. 2011) |
| 2009 | Venue-based with peer-referral at each venue | 200 | Cape Town | 25.5% (19.6-32.1) | (Baral, Burrell et al. 2011) |
| 2010 | Outreach workers and through peer-referral. | 316 | Cape Town | Not available | (Tucker, Liht et al. 2013, Tucker, Liht et al. 2014) |
| 2012  2012/13  2012/13 | RDS | 286  290  349 | Cape Town  Durban  Johannesburg | 22.3% (14.7–30.1)  48.2% (37.9–55.4)  26.8% (20.4–35.6) | (Cloete, Jooste et al. 2014) |
| 2012 | MSM attending a men’s Health clinic | 200 | Cape Town | 44.0% (37.0-51.2) | (Rebe, Lewis et al. 2015, Muller, Rebe et al. 2016) |
| 2012 | Previously-developed lists of MSM and snowball sampling. | 34 | Cape Town and Port Elizabeth | Not available | (Siegler, Voux et al. 2014) |
| 2015 | Event- and venue-based, online, participant referral, and walk-ins at study clinics. | 115  177 | Cape Town  Port Elizabeth | 30.4% (22.7-39.5)  50.8% (43.5-58.2) | (McNaghten, Kearns et al. 2014) |
| Overall female | | | | | |
| 2002 | Multi-stage stratified cluster sampling | 4656 | National | 17.7% (15.2-20.4) | (Human Sciences Research Council 2002) |
| 2005 | Multi-stage stratified cluster sampling | 6547 | National | 20.2% (18.3-22.2) | (Human Sciences Research Council 2005) |
| 2008 | Multi-stage stratified cluster sampling | 8327 | National | Not available | (Human Sciences Research Council 2008) |
| 2012 | Multi-stage stratified cluster sampling | 9982 | National | 23.2% (21.3-25.1) | (Human Sciences Research Council 2012) |
| 2017 | Multi-stage stratified cluster sampling | 12,900 aged 15-64 | National | 26.3% (24.5-28.2) |  |
| Overall male | | | | | |
| 2002 | Multi-stage stratified cluster sampling | 3772 | National | 12.80% | (Human Sciences Research Council 2002) |
| 2005 | Multi-stage stratified cluster sampling | 4078 | National | 11.7% (10.0-13.6) | (Human Sciences Research Council 2005) |
| 2008 | Multi-stage stratified cluster sampling | 5501 | National | Not available | (Human Sciences Research Council 2008) |
| 2012 | Multi-stage stratified cluster sampling | 7561 | National | 14.5% (12.8-16.3) | (Human Sciences Research Council 2012) |
| 2017 | Multi-stage stratified cluster sampling | 9667 aged 15-64 | National | 14.8% (13.3-16.5) |  |
| RDS: Respondent-driven sampling | | | | | |

## Supplementary Table 3. Demographic, sexual and behavioural parameters of female sex workers and clients.

| Parameter definition | Parameter Symbol | Clients | FSW | References |
| --- | --- | --- | --- | --- |
| Proportion of adults that are clients or FSW |  | To balance commercial sex acts | 0.69-0.96% | (SWPSSES 2013): A sex worker size estimation study estimated 0.69-0.96% of adult females are female sex workers. We do not have client population size estimates except from 2005/08 general population HSRC surveys(Human Sciences Research Council 2005, Human Sciences Research Council 2008) which showed that <2% of adult men reported having commercial partners, which will be an under estimate. In their modelling of SA, Bekker and Johnson assumed a high estimate of 35%(Bekker, Johnson et al. 2015). In a review of size estimate studies of males who report having paid sex, Carael found the median estimate of the proportion of men in Southern Africa who paid to have sex in the last 12 months to be 7.0% (IQR: 1.7-10.6%) (Carael, Slaymaker et al. 2006). Another survey found that that 6.4% (95%CI: 3.5-10.8%) of men reported having ever paid for sex(Quaife, Eakle et al. 2016). However, in light of the limited data, and its possible uncertainty and biases we calibrate the client population size to balance the number of commercial sex acts that FSW and clients report. We impose an upper bound of 35% to the proportion of men that are clients based on the assumption by Bekker and a high estimate of ~30% in Benin(Behanzin, Diabate et al. 2013). |
| Frequency of FSW per client and client per FSW per year | $n_{3}^{co}$, $n_{4}^{co}$ | 2.3-72.8 per year | 100-1000 per year | (SAHMS-FSW 2014) reported a min and max range of 50-200 paying clients in last 6 months across 3 cities. (Quaife, Eakle et al. 2016) reported a mean of 35.9 (27.3-44.6) and median of 20 (IQR 10-40 across 3 age groups) clients in previous week. A survey in Port Elisabeth among FSW (PEFSW 2015) had a mean of 5.4 (95%CI: 4.8-6.2) and median of 4 (IQR 0-7) new clients in the last 30 days and mean of 6.9 (95%CI 6.3-7.6) and median 5 (IQR: 3-9) regular clients in last 30 days. The same study reported a mean of 3.8 (2.7-4.9) clients per day with a median of 15 (12-20) days of sex work per month. We assume a lower bound from the SAHMS study as it was a combination of FSW in 3 different cities. Upper bound is a compromise between studies that have reported very high frequency and those with low frequency of clients. We also consider that regular FSW also take time off sex work and therefore have frequency of clients per FSW of (100-1000).  In terms of FSWs seen by clients, the HSRC surveys (Human Sciences Research Council 2005) and (Human Sciences Research Council 2008) report 2.3 and 3.4 commercial partners in the last year among males who report having them in last 12 months which seems low. Conversely, in the Port Elisabeth client survey, clients report a median of 2 (IQR: 1-4) and mean of 3.5 (95%CI: 3.3-4.3) commercial sex acts in the past 3 months. In the Klerksdorp client survey, clients reported a median of 3 (IQR: 2-4) and an average of 3.4 (95%CI: 2.8-3.9) commercial sex acts in the past month and a median of 1 (IQR: 1-1) and average of 1.3 (95%CI: 1.1-1.4) commercial sex acts in the past week. We assume lower bound from the HSRC data and upper bound from the Klerksdorp survey. |
| Percentage of commercial sex acts that are anal | $p_{3}^{co}$, $p_{4}^{co}$ | 0.6-9.3% | 0.6-9.3% | On average, in the Port Elisabeth surveys, FSW report 2.2% (95%CI 1.1-3.3%) of commercial sex acts are anal (PEFSW 2015, Rao, Baral et al. 2016). On average, clients report 3.9% (95%CI: 3.0-4.9) of commercial sex acts are anal. We assumed the amount of AI by Clients and FSW was the same and took the lower bound from what FSWs report and upper bound from what clients report to produce a range. We incorporate additional uncertainty by halving and doubling the odds associated with the lower and upper values in the range. |
| Condom use VI with commercial partners | $\pi_{3v}^{co}$, $\pi_{4v}^{co}$ | Time varying | Time varying | See separate section on condom use trends |
| Condom use for AI with commercial partners | $\pi_{3v}^{co}$, $\pi_{4v}^{co}$ | Time varying | Time varying | See separate section on condom use trends |
|  |  |  |  |  |
| Proportion of clients and FSW that have a main partner in last 12 months | $p_{3}^{m}$, $p_{4}^{m}$ | 0.81-0.98 | 0.25-0.90 | In the Ekurhuleni survey, 0.85 (0.80-0.90) of FSW reported having any main partners (Quaife, Eakle et al. 2016). In contrast in the (SAHMS-FSW 2014), 0.25-0.52 of FSW had main partners in the last 6 months. Lastly, in the PE survey, 0.66 (0.61-0.70) of FSW reported having a long-term partner in the last 12 months (PEFSW 2015, Rao, Baral et al. 2016). For FSWs, we assumed the minimum and maximum values from these FSW surveys.  In the PE client survey, 84.5% (95%CI: 81.2-87.3) of clients report having a main female partner in the last 12 months. In the Klerksdorp client survey, 94.3% (95%CI: 88.1-97.9%) of clients had a main partner in the last 12 months. For clients, we assumed the minimum and maximum from these surveys. |
|  |  |  |  |  |
| Frequency of main partners for clients and FSW among those with main partners (per year) | $n_{3}^{m}$, $n_{4}^{m}$ | 1.0-2.9 per year | 1.0-3.07 per year | (SAHMS-FSW 2014) report a median of 1 and mean of 1.02-3.07 (95%CI range across settings) main partners for FSW in the previous 6 months. Among those with commercial partners, females had an average of 1.2 main partners and males 1.3 in the 2005 HSRC survey (Human Sciences Research Council 2005), while in 2008 females report 0.68 and males had reported 2.7 in the last 12 months (Human Sciences Research Council 2008). In the PE survey, the frequency of main partners among FSW who report sex with non-paying partners in the last 30 days was 0.91 (0.24-1.58) and among those who report long term partners in the last 12 months was 1.12 (0.68-1.7) we took the upper and lower bounds from these estimates for FSWs  In Port Elisabeth, Clients with at least one main partner report a median of 1 (IQR: 1-2) and average of 2.45 (95%CI: 2.05-2.86) main partners in the past 12 months. In Klerksdorp, clients with at least one main partner report a median of 1 (IQR: 1-1) and average of 1.4 (95%CI: 1.1-1.6) main partners in the past 12 months. We took the upper and lower bounds from these estimates for clients. |
| Frequency of vaginal sex with main partners among those reporting main partners for clients and FSW per year | $\psi_{3v}^{m},\psi_{4v}^{m}$ | 6-144 per year | 24-144 per year | In the 3 city FSW survey, 15-60 (95%CI across cities) or 8-90 (IQR across cities) vaginal sex acts in the last 6 months were reported by FSW with their main partners (SAHMS-FSW 2014). In the Port Elisabeth survey, the number of vaginal sex acts with long term partners among those who have them was reported as 8 (2.0-12.0) in the last 30 days (PEFSW 2015, Rao, Baral et al. 2016), while number of sex acts was 4.0 (2.9-5.0) in the previous month in the Ekurhuleni FSW survey (Quaife, Eakle et al. 2016). Assume minimum and maximum from these FSW surveys including the 95%CI from (SAHMS-FSW 2014).  In Port Elisabeth, clients report a median of 3 (IQR: 1-6) and an average of 5.2 (95%CI: 4.6-5.9) vaginal sex acts with main partners in the past 30 days. For clients, we use the IQR from PE and we incorporate additional uncertainty by halving and doubling the lower and upper bounds. |
| Frequency for AI with main partners for clients and FSW per year | $\psi_{3a}^{m},\psi_{4a}^{m}$ | 0-7.2 per year | 1.6-60.0 per year | In the 3 city FSW survey, FSW reported 1.0-36.0 (IQR across cities) or 2.0-30.0 (95%CI across cities) anal sex with main partners in last 6 months (SAHMS-FSW 2014). In the Port Elisabeth FSW survey, FSWs reported 1.3 (0.13-2.45) anal sex acts with their long term partners in last 30 days (PEFSW 2015, Rao, Baral et al. 2016) whereas FSW in the Ekurhuleni survey reported a mean of 0.35 AI acts with regular partners in the last month (Quaife, Eakle et al. 2016). Consider range of frequency of anal sex as minimum and maximum from these studies.  In Port Elisabeth, clients report a median of 0 (IQR: 0-0) and an average of 0.17 (95%CI: 0.02-0.3) anal sex acts with main partners in the past 30 days. For clients, we consider lower bound for clients from the lower IQR and upper bound from the 95% CI and we incorporate additional uncertainty by halving and doubling the lower and upper bounds. |
| Condom use VI with main partners for clients and FSW | $\pi_{3v}^{m}$, $\pi_{4v}^{m}$ | Time varying | Time varying | See section on condom use trends |
| Condom use AI with main partners for clients and FSW | $\pi_{3a}^{m}$, $\pi_{4a}^{m}$ | Time varying | Time varying | See section on condom use trends |
| Proportion of clients and FSW with casual partners | ${p_{3}^{c},p}_{4}^{c}$ | 0.534-0.979 | 0.056-0.29 | In the 3 city FSW survey (SAHMS-FSW 2014), 5.6-29.2% of FSW reported casual partners in the last 6 months. In the Port Elisabeth FSW survey, 12-19% of FSW had casual partners in the last year (PEFSW 2015, Rao, Baral et al. 2016), while in the Ekurhuleni FSW survey, 67.0% (95%CI: 60.5-73.5) of FSW reported having casual partners in the previous year (Quaife, Eakle et al. 2016). We did not use data from the Ekurhuleni survey because it was not consistent with other surveys, and so use the minimum and maximum from the other surveys.  In the Port Elisabeth client survey, 57.6% (95%CI: 53.4-61.7) of clients reported having a casual female partner in the last 3 months. In the Klerksdorp client survey, 94.4% (95%CI: 88.3-97.9) of clients reported having a casual partner in the past year. We took the upper and lower bounds from these estimates for clients. |
| Frequency of casual partners for clients and FSW per year among those with casual partners | ${n_{3}^{c},n}_{4}^{c}$ | 1.1-15.1 per year | 1.0-18.0 per year | In the 3 city FSW survey (SAHMS-FSW 2014), FSW had 1-5 (range of 95% CI across cities) or 1-9 (for range of IQR across cities) casual partners in the previous 6 months. In the Port Elisabeth FSW survey, FSW (PEFSW 2015, Rao, Baral et al. 2016) had a mean of 1.38 (0.34-2.41) casual partners in the past year. Lastly, In the HSRC 2005 survey, females report 0.85 non-regular partners among those with commercial partners in last 12 months (Human Sciences Research Council 2005). We assume the range from these estimates.  In the HSRC 2005 survey, men report an average of 1.1 non-regular partners among those with commercial partners in last 12 months (Human Sciences Research Council 2005). Conversely, in the Port Elisabeth client survey, Clients report a median of 2 (IQR: 1-3) and average of 3.0 (95%CI: 2.6-3.4) casual partners in the past 3 months. In the Klerksdorp survey, Clients report a median of 8 (IQR: 5-11) and average of 12.0 (95%CI: 8.8-15.1) casual partners in the past year. We assume the range from these estimates. |
| Frequency of VI acts for clients and FSWs with casual partners among those with casual partners per month | $\psi_{3v}^{c},\psi_{4v}^{c}$ | 0.5-10.2 per month | 0.2-8.3 per month | In the 3 city FSW survey (SAHMS-FSW 2014), FSW reported 1-33 (range of 95%CI across 3 cities, 1-50 if range of IQR) VI with casual partners in the last 6 months, whereas the port Elisabeth survey reported a median of 3 (1.5-4.5) in the last 30 days (PEFSW 2015, Rao, Baral et al. 2016). We used the range in the monthly estimates to give a sampling range.  In the Port Elisabeth client survey, clients report a median of 2 (IQR: 1-4) and a mean of 4.2 (95%CI: 3.5-5.1) vaginal sex acts with casual partners in the past 30 days. Because the data is skewed, we consider lower bound for clients from the lower IQR and upper bound from the 95% CI in the PE clients survey and we incorporate additional uncertainty by halving and doubling the lower and upper bounds. |
| Frequency of AI acts for clients and FSWs with casual partners among those with casual partners per month | $\psi_{3a}^{c},\psi_{4a}^{c}$ | 0.0-0.52 per month | 0.0-3.3 per month | In the 3 city FSW survey (SAHMS-FSW 2014), FSW reported 1.0-17.0 (IQR across cities) or 1.75-20.0 (95%CI across cities) AI sex acts with casual partners in the last 6 months, while another FSW survey from Ekurhuleni reported a mean of 0-5.6 acts in the last year (Quaife, Eakle et al. 2016). FSW in the port Elisabeth survey reported no AI with casual partners. We used the range of the monthly estimates to give a sampling range.  In Port Elisabeth, clients report a median of 0 (IQR: 0-0) and an average of 0.1 (95%CI: 0.04-0.2) anal sex acts with casual partners in the past 30 days. We consider the lower bound for clients from the lower IQR (0) and upper bound from the range assumed for low risk males (0.26). We incorporate additional uncertainty by halving and doubling the lower and upper bounds. |
| Condom use VI with casual partners for clients and FSWs | $\pi_{3v}^{c}$, $\pi_{4v}^{c}$ | Time varying | Time varying | See separate section |
| Condom use AI with casual partners for clients and FSWs | $\pi_{3a}^{c}$, $\pi_{4a}^{c}$ | Time varying | Time varying | See separate section |
|  |  |  |  |  |
| Duration of buying sex and sex work for clients and FSWs in years | $1/\gamma_{0},1/\gamma_{1}$ | 1.5-36 years | 3.2-8.1 years | In the 3 city FSW survey (SAHMS-FSW 2014), FSW reported a duration of sex work IQR of 3-10 years with a mean of 7.3 years, while the port Elisabeth FSW survey (PEFSW 2015, Rao, Baral et al. 2016) reported a mean duration of 5.4 (2.4-8.4) years and median of 4 years with IQR of (2.0-7.0). In the Ekurhuleni FSW survey, the min and max from the IQR for the duration of sex work of the 3 age groups is 0-6 years (Quaife, Eakle et al. 2016).  We used a simple model of FSW initiation, aging and cessation to estimate cessation rates using data from SAHMS. This data gives a range of possible cessation rates of 3.2-8.1 years, agreeing with data from Port Elizabeth and Ekurhuleni.  In the Port Elisabeth client survey, clients report a median of 11.5 (IQR: 6-18) years and mean of 12.9 (95%CI: 12.0-13.7) years duration of buying sex. In the Klerksdorp client survey, clients report a median of 5 (IQR: 3-8.5) years and mean of 6.4 years (95%CI: 5.4-7.4) of buying sex. We multiply the lower bound by 0.5 and upper bound by 2 because of uncertainty in how overall duration of sex work relates to current duration of sex work. |
| Relative difference in ART coverage between clients and all males and FSWs and all females. |  | 0.7-1.0 | 0.7-1.0 | In the port Elisabeth FSW survey, 102 FSW reported that they were currently on ARVs (PEFSW 2015), out of 261 FSW that tested HIV+ (39.1% (33.1-45.3%) coverage). Among self-disclosed HIV positive FSW from the 3 city FSW survey, 373 reported current ART uptake, giving an ART coverage of 29.6% (27.1-32.2%) among HIV+ FSWs (SAHMS-FSW 2014). In the Ekurhuleni FSW survey, out of 81 FSW who reported that they were HIV+, 48 reported currently being on ART (Quaife, Eakle et al. 2016), giving ART coverage of 59.3% (47.8-70.0%). In the Soweto FSW survey, 118 out of 269 (43.9%; 95%CI: 37.8-50.0) FSW testing HIV positive self-reported current treatment, with 99 (70%) of those with viral loads less than 400 copies/ml (Coetzee, Hunt et al. 2017). We compare this with UNAIDs estimates for females [2013: 42 (31-51); 2014: 48 (36-56); 2015: 53 (40-65); 2016: 59 (45-73)] and assume ART coverage among FSW is 0.7-1.0 times that among all females.  For clients, the Port Elisabeth survey (2017-18) found 31/83 (37.3%, 95%CI: 27.5-48.4) of HIV positive clients reported being on ART and 29.2% (95%CI: 19.7-40.4%) of HIV positive clients were virally supressed (< 1000 copies/ml). In the Klerksdorp survey, 32.3% (95%CI: 16.8-51.4%) of HIV positive clients reported being on ART and 8/30 (26.7%, 95%CI: 9.9-42.3) of clients were virally supressed (<1000 copies/ml).  UNAIDs coverage among all males is 53% (38-66%) in 2017 so assume ART coverage among males is 0.7-1 times that among all males. |
| Proportion of male adults that are circumcised. | $\zeta$ | Time varying | Not applicable. | See section on circumcision trends. |

## Supplementary Table 4. Demographic, sexual and behavioural parameters of low risk males and females.

| Parameter definition | Parameter Symbol | Low risk male | Low risk female | References |
| --- | --- | --- | --- | --- |
| Proportion of LRF and LRM with main partner | $p_{1}^{m},p_{2}^{m}$ | 0.75-0.83 | 0.71-0.77 | Proportion of males and females with regular partners are (0.75-0.83) and (0.71-0.77) from HSRC general population surveys in 2005 and 2008 (Human Sciences Research Council 2005), (Human Sciences Research Council 2008). Conversely, in the survey from Ekurhuleni, 0.92 (0.88-0.96) of males and 0.88 (0.84-0.93) of females reported having any regular partners (Quaife, Eakle et al. 2016). Just use HSRC data because more representative than the Ekurhuleni survey. |
| Frequency of main partners among those with main partners for LRF and LRM per year | $n_{1}^{m},n_{2}^{m}$ | 1.10-2.8 per year | 1.0-1.3 per year | Male and female report 1.12 (1.06-1.22) and 1.01 (1.00-1.02) regular partners in the last 12 months, respectively, in the HSRC surveys (Human Sciences Research Council 2005), (Human Sciences Research Council 2008). Conversely, in the Ekurhuleni survey, the number of regular partners reported by male and female in the previous year was 2.1 (1.4-2.8) and 1.2 (1.1-1.3), respectively (Quaife, Eakle et al. 2016). Used range including quaife and HSRC data. |
| Frequency of VI sex with main partners for LRM and LRF partners per year | $\psi_{1v}^{m},\psi_{2v}^{m}$ | 42.0-70.8 per year | 42.0-70.8 per year | Frequency of sex in last 30 days among those with regular partners was (4.6-5.9) for male and (3.7-4.6) for female in the 2005 HSRC survey (Human Sciences Research Council 2005). Conversely, in the Ekurhuleni study (Quaife, Eakle et al. 2016), males and females reported 3.8-5.7 and 3.5-5.1 sex acts in the previous month, respectively. Assume (3.5-5.9) per month for both |
| Frequency of AI sex with main partners for LRM and LRF per year | $\psi_{1a}^{m},\psi_{2a}^{m}$ | 1.2-13.2 per year | 1.2-13.2 per year | In the Ekurhuleni survey, males and females report a mean of 0.93-0.99 and 0.96-1.0 anal acts in last month with regular partners (Quaife, Eakle et al. 2016). There was no data on this from the HSRC study.  A systematic review of heterosexual anal sex in South Africa found that across 7 studies frequency of anal sex varied between 0.1-1.1 per month (Owen, Elmes et al. 2017). Only one study reported frequency of anal sex by partnership type - a 2003 study in Soweto of HIV negative males and females. Study participants reported an average of 0.4 and 0.1 anal sex acts with steady partners and casual partners in the past 6 months, respectively. (Andersson, Van Niekerk et al. 2009). We use the range found in the systematic review for both males and females, as this range includes estimates from the Ekurhuleni study, which suggests similar frequency of anal sex with main partners by gender. |
| Condom use for VI with main partner for LRM and LRF | $\pi_{3v}^{m}$, $\pi_{4v}^{m}$ | Time varying | Time varying | See separate section |
| Condom use AI with main partner | $\pi_{3a}^{m}$, $\pi_{4a}^{m}$ | Time varying | Time varying | See separate section |
| Proportion of LRM and LRF with casual partners | $p_{1}^{c},p_{2}^{c}$ | 0.08-0.49 | 0.02-0.27 | In the HSRC surveys, a proportion of 1.6-3.0% for female and 8.0-9.0% for males had non-regular partners in last 12 months (Human Sciences Research Council 2005), (Human Sciences Research Council 2008). Conversely, the Ekurhuleni survey found that 0.43 (0.36-0.49) of males and 0.21 (0.16-0.27) of females reported having any casual partners in the previous year (Quaife, Eakle et al. 2016) . Assume size of group with casual partners is larger than that reported in 2005/08 HSRC. Assume lower bound reported in (Human Sciences Research Council 2005), (Human Sciences Research Council 2008) and upper bound as reported from (Quaife, Eakle et al. 2016). |
| Frequency of casual partners for LRM and LRF per year | $n_{1}^{c},n_{2}^{c}$ | 1.7-2.9 per year | 1.7-2.9 per year | Data direct from HSRC surveys for those that report these non-regular partnerships (Human Sciences Research Council 2005), (Human Sciences Research Council 2008) |
| Frequency of VI sex with casual partners for LRF and LRM per month | $\psi_{1v}^{c},\psi_{2v}^{c}$ | 1.1-4.3 per month | 1.1-4.3 per month | In the Ekurhuleni survey, males and females report 2.1-4.3 and 1.1-4.1 sex acts in previous month with casual partners among those reporting casual partners (Quaife, Eakle et al. 2016). Data from old RHRU survey in 2000 shows 1.5-3.0 sex acts with casual partners. No data on this from the HSRC surveys. Use lower and upper bounds. |
| Frequency of AI sex with casual partners for LRF and LRM per month | $\psi_{1a}^{c},\psi_{2a}^{c}$ | 0.02-0.26 per month | 0-0.22 per month | In the Ekurhuleni survey, males and females report a mean of 0.75-3.1 and 0-0.74 anal sex acts with casual partners in the last year, respectively (Quaife, Eakle et al. 2016).  A systematic review of heterosexual anal sex in South Africa found that across 7 studies frequency of anal sex varied between 0.1-1.1 per month (Owen, Elmes et al. 2017). Only one study reported frequency of anal sex by partnership type - a 2003 study in Soweto of HIV negative males and females. Study participants reported an average of 0.4 and 0.1 anal sex acts with steady partners and casual partners in the past 6 months, respectively. (Andersson, Van Niekerk et al. 2009). Based on the Soweto and Ekurhuleni studies, we assume frequency of anal sex with casual partners is at most ¼ of that amongst main partners. Applying this to the range found in the systematic review gives 0.02-0.22. We assume this range applies to both males and females as a Cape Town study found similar levels of frequency of anal sex among young men and women (Simbayi, Kalichman et al. 2005). We use the minimum and maximum from the Ekurhuleni study and the adjusted range in the systematic review. |
| Condom use VI with casual partners for LRM and LRF | $\pi_{3v}^{c}$, $\pi_{4v}^{c}$ | Time varying | Time varying | See separate section |
| Condom use with casual partner for AI | $\pi_{3a}^{c}$, $\pi_{4a}^{c}$ | Time varying | Time varying | See separate section |
| ART coverage among adult male and female who are HIV+ |  | Time varying | Time varying | ART coverage trends from UNAIDS (UNAIDS-AIDSinfo) |
| Inflow of HIV positive into the adult population | $\phi_{1},\phi_{2}$ | Time varying | Time varying | Data from the HSRC studies suggest the proportion of 15-year old HIV positive females who enter the adult population is 4.9% (0.98-21.6%) in 2002, 7.5% (3.7-14.7%) in 2005 and 3.7% (1.6-8.4%) in 2008. The corresponding proportion of HIV positive 15-year-old males is 6.7% (1.6-24.4%), 1.6% (0.56-4.8%) and 1.5% (0.38-6.8%), respectively (Human Sciences Research Council 2002, Human Sciences Research Council 2005, Human Sciences Research Council 2008). We assume the inflow of HIV positive male and female increase linearly from 0 in 1995 to the values in 2002, 2005, 2008 and stay constant thereafter. |
| Proportion of male adults that are circumcised. | $\zeta$ | Time varying | Not applicable. | See section on circumcision trends. |

For all the SAHMS estimates where both 95%CI and IQR across the three sites are given, we used the 95%CI range.

## Supplementary Table 5. Demographic, sexual and behavioural parameters of men who have sex with men.

| Parameter definition | Parameter Symbol | Male partners | Female partners | References |
| --- | --- | --- | --- | --- |
| Proportion of male adults that are MSM |  | 0.65-7.3% | | Cross-sectional household survey in 2008 (Dunkle, Jewkes et al. 2013) – in 3 adjoining districts of the Eastern Cape and KwaZulu-Natal provinces of South Africa found 4.2% (95%CI: 3.3-5.3) reported lifetime consensual sex (oral, anal or unspecified). Baseline questionnaire in 2002-3 for RCT amongst sexually experienced Xhosa males (Jewkes, Dunkle et al. 2006) found 3.6% (95%CI: 2.6-4.8) had had sexual contact with a man (~3/4 of whom report having only one such event which was coerced). 3.2% of men who participated in the 2008 HSRC household survey self-reported same-sex behaviour (Human Sciences Research Council 2008). 176/2769 (6.4% 95%CI: 5.5-7.3%) men surveyed in urban townships and a STI clinic in Cape Town reported same sex partners in the last 3 months, of which 51% had engaged in anal intercourse (Kalichman, Simbayi et al. 2009). |
| Age at which MSM first have sex with a man |  | 15-19 | | 2008 study in Johannesburg and Durban (Rispel, Metcalf et al. 2011) reports a median age at first sex with a man of 17.0 years, range: 5-32; mean: 16.7. |
| Proportion of MSM that have a main partner in last 12 months | $p_{5}^{m,m}$, $p_{6}^{m,m}$,  $p_{5}^{m,f}$, $p_{6}^{m,f}$ | 46.0-77.5% | 2.8-40.6% | 8% (16/200) of MSM in Cape Town reported having a regular female partner in 2009 (Baral, Burrell et al. 2011). In the South Africa Marang Men’s project (2012-13), 2.8%, 16.0% and 38.7% of MSM reported having a regular female partner in Durban, Cape Town and Johannesburg, respectively (Cloete, Jooste et al. 2014). In the Mpumalanga Men’s study (2012-2013), 25.7% (95%CI: 18.05-33.2) and 40.6% (95% CI 32.2-48.9) of MSM in Gert Sibande and Ehlanzeni, respectively, reported having a regular female partner (Lane, Osmand et al. 2014). In the Soweto Men’s Project (2008), 51.2% (RDS adjusted: 63.4%, 95%CI: 55.9-70.8) of MSM reported having a regular female partner (Lane, Raymond et al. 2011). In the HIVST study (2015), 69.1% and 37.5% of **HIV negative** MSM reported having a regular female partner in Gert Sibande and Ehlanzeni, respectively (Lippman, Lane et al. 2018). A 2012 cross-sectional survey of MSM attending a MSM sexual health care clinic found that 22.5% of MSM reported having any female sexual partners in the last 12 months (Rebe, Lewis et al. 2015). In the Sibanye Health Project (2015), 14.5% (95%CI: 10.0 – 20.7) and 15.3% (95%CI: 9.7 – 23.3) of MSM in Port Elizabeth and Cape town, respectively, report having a regular female partner. In the Johannesburg/eThekwini Men’s Study (2008), 64.2% (95%CI: 58.3-69.8) of MSM in Johannesburg and Durban reported never having sex with a woman (Rispel, Metcalf et al. 2011). We do not use the HIVST study, as this is a sample of HIV negative MSM only, or the Soweto Men’s project, as this is inconsistent with other data. We use the minimum and maximum from the other surveys.  In the Johannesburg/eThekwini Men’s Study (2008), 62.4% (95%CI: 56.6-68.1) of MSM in Johannesburg and Durban reported having a male main partner (Rispel, Metcalf et al. 2011). In 2008, 55.4% (95%CI: 49.6-61.2) of MSM in Pretoria reported being in a steady relationship with another man (Knox, Yi et al. 2010). In the Soweto Men’s Project (2008), 73.0% (RDS adjusted: 69.6%, 95%CI: 64.7-76.7) of MSM reported having a regular male partner (Lane, Raymond et al. 2011). 46% (95%CI: 41.8-56.1) of MSM in Cape Town reported having a regular male partner in 2009 (Baral, Burrell et al. 2011). A 2012 cross-sectional survey of MSM attending a MSM sexual health care clinic found that 52% (95% CI: 44.8-59.1%) of MSM reported having a male partner who was their primary sexual partner (Rebe, Lewis et al. 2015). In the South Africa Marang Men’s project (2012-13), 64.8%, 62.8% and 62.9% of MSM reported having a regular male partner in Durban, Cape Town and Johannesburg, respectively (Cloete, Jooste et al. 2014). In the Mpumalanga Men’s study (2012-2013), 66.0% (95% CI: 58.2-73.7) and 74.2% (95%CI: 65.9-79.9) of MSM in Gert Sibande and Ehlanzeni, respectively, reported having a regular male partner (Lane, Osmand et al. 2014).In the HIVST study (2015), 80.0% and 86.1% of **HIV negative** MSM reported having a regular male partner in Gert Sibande and Ehlanzeni, respectively (Lippman, Lane et al. 2018). In the Sibanye Health Project (2015), 73.6 (95%CI: 66.2-79.9) and 77.5% (95%CI: 68.2-85%) of MSM in Port Elizabeth and Cape town, respectively, report having a regular male partner.  We do not use the HIVST study, as this is a sample of HIV negative MSM only, and use the minimum and maximum from the other surveys. |
|  |  |  |  |  |
| Frequency of main partners per year for young MSM among those with main partners in the past 12 months | $n_{5}^{m,m}$, $n_{6}^{m,m}$,  $n_{5}^{m,f}$, $n_{6}^{m,f}$ | 1-2.01 per year | 1-2.68 per year | In the Sibanye Health Project (2015), young MSM with main male partners reported a median of 1 (IQR 1:2) in both Cape Town (mean: 1.79, 95%CI 1.57-2.01) and Port Elizabeth (mean: 1.47, 95%CI 1.35-1.59) male main partners in the last 12 months. Use median as lower bound and the maximum of the upper 95% confidence limits as the upper bound.  In the Sibanye Health Project (2015), young MSM with main female partners reported a median of 1 (IQR 1:2) and 1 (IQR 1-1) female main partners in the last 12 months in Cape Town (mean: 2.0, 95%CI 1.32-2.68) and Port Elizabeth (mean: 1.23, 95%CI 1.08-1.39), respectively. Use median as lower bound and the maximum of the upper 95% confidence limits as the upper bound. |
| Relative increase in frequency of main partners if age>30 vs age<30. |  | 1.09-1.54 | 0.44-0.94 | In the Sibanye Health Project (2015), amongst men with at least 1 main male partner in the past 12 months, old MSM have (aIRR adjusted for site) 1.30 (95%CI: 1.09-1.54) times more main male partners than young MSM.  In the Sibanye Health Project (2015), amongst men with at least 1 main female partner in the past 12 months, young MSM had 0.65 (95%CI: 0.44-0.94) times less main female partners than old MSM. |
| Frequency of vaginal sex with main partners per year among those reporting main partners | $\psi_{5v}^{m},\psi_{6v}^{m}$ | Not applicable | 12-120 per year | In the Sibanye Health Project (2015), MSM in Cape Town and Port Elizabeth reported a median of 7.25 (IQR 3-30) vaginal sex acts with female main partners in the last 3 months (mean: 16.4, 95%CI: 10.3-22.4). |
| Frequency of AI per year with main partners among those reporting main partners. | $\psi_{5a}^{m,m},\psi_{6a}^{m,m}$,  $\psi_{5a}^{m,f},\psi_{6a}^{m,f}$ | 12-120 per year | 0-28 per year | In the Sibanye Health Project (2015), MSM in Cape Town reported a median of 6 (IQR 3-10) anal sex acts with male main partners in the last 3 months (mean: 8.9, 95%CI: 6.5-11.2); MSM in Port Elizabeth reported a median of 6 (IQR 3:30) anal sex acts with male main partners in the last 3 months (mean: 13.9, 95%CI 11.1-16.7).  In the Sibanye Health Project (2015), MSM in Cape Town and Port Elizabeth reported a median of 3 (IQR 0-7) anal sex acts with female main partners in the last 3 months (mean: 6.7, 95%CI: 1.9-11.5). |
| Condom use VI with main partners | $\pi_{5v}^{m}$, $\pi_{6v}^{m}$ | Not applicable | Time varying | See section on condom use trends |
| Condom use AI with main partners. | $\pi_{5a}^{m,m}$, $\pi_{6a}^{m,m}$,  $\pi_{5a}^{m,f}$, $\pi_{6a}^{m,f}$ | Time varying | Time varying | See section on condom use trends |
| Proportion of MSM with casual partners | ${p_{5}^{c,m},p}_{6}^{c,m}$  ${p_{5}^{c,f},p}_{6}^{c,f}$ | 60.1-74.4% | 6.5-17.0% | In the Sibanye Health Project (2015), 73.7% (95%CI: 64.1-81.5) and 67.7% (95%CI: 60.1-74.4) of **all** MSM in Cape town and Port Elizabeth, respectively, report having a casual male partner.  In the Sibanye Health Project (2015), 9.6% (95%CI: 6.5 – 14.0) and 13.3% (95%CI: 10.3- 17.0) of **young** MSM in Cape town and Port Elizabeth, respectively, report having a casual female partner. |
| Increase in the odds of having a casual partner if old (>30) vs young (<30) |  | - | 1.02-2.42 | In the Sibanye Health Project (2015), old MSM have 1.57 higher odds (95%CI: 1.02-2.42) of having a casual female partner.  There was no difference between the proportion of young (74.3%; 95%CI: 68.1-79.7) and old (72.0% 95%CI: 60.6-81.2) MSM who report having a casual male partner. |
| Frequency of casual partners per year among Young MSM with casual partners | $n_{5}^{c}$ | 1-4 per year | 1-6.5 per year | In the Sibanye Health Project (2015), young MSM reported a median of 2 (IQR 1-3) and 2 (IQR: 1-4) male casual partners in the last 12 months in Cape Town (mean: 3.2, 95%CI: 2.6-3.8) and Port Elizabeth (mean: 2.74, 95%CI: 2.39-3.08), respectively.  In the Sibanye Health Project (2015), young MSM reported a median of 2 (IQR 1-2) and 2 (IQR: 1-6.5) female casual partners in the last 12 months in Cape Town (mean: 3.9, 95%CI: 1.2-6.5) and Port Elizabeth (mean: 3.8, 95%CI: 1.3-6.2), respectively. |
| Relative increase in frequency of casual partners if age>30 vs age<30. |  | 1.12-1.59 | 1.06-2.21 | In the Sibanye Health Project (2015), amongst men with at least 1 male casual partner in the past 12 months, old MSM have (aIRR, adjusted for site) 1.34 (95%CI: 1.12-1.59) times more casual male partners than young MSM.  In the Sibanye Health Project (2015), amongst men with at least 1 female casual partner in the past 12 months, old MSM have (aIRR, adjusted for site) 1.53 (95%CI: 1.06-2.21) times more casual female partners than young MSM. |
| Frequency of VI acts with casual partners among those with casual partners per partner | $\psi_{3v}^{c},\psi_{4v}^{c}$ | Not applicable | 2-6.5 | In the Sibanye Health Project (2015), MSM in Cape Town and Port Elizabeth reported a median of 3.5 (IQR 2-6.5) vaginal sex acts with female casual partners in the last 3 months (mean: 3.9, 95%CI: 2.4-5.4). |
| Frequency of AI acts with casual partners among those with casual partners per month | $\psi_{3a}^{c},\psi_{4a}^{c}$ | 2-10.7 per month | 0-5 per month | In the Sibanye Health Project (2015), MSM in Cape Town reported a median of 3 (IQR 2:6) anal sex acts per male casual partners in the last 3 months (mean: 4.2, 95%CI: 3.2-5.2); MSM in Port Elizabeth reported a median of 3 (IQR 2:6) anal sex acts per male casual partners in the last 3 months (mean: 7.9, 95%CI 5.1-10.7).  In the Sibanye Health Project (2015), MSM in Cape Town and Port Elizabeth reported a median of 2 (IQR 0-5) anal sex acts per female casual partners in the last 3 months (mean: 2.5, 95%CI: 1.0-4.1).  We assume that all sex acts with casual partners occurred over 1 month. |
| Condom use VI with casual partners. | $\pi_{3v}^{c}$, $\pi_{4v}^{c}$ | Time varying | Time varying | See separate section |
| Condom use AI with casual partners | $\pi_{3a}^{c}$, $\pi_{4a}^{c}$ | Time varying | Time varying | See separate section |
| Relative difference in ART coverage between MSM and all males. |  | 0.3-0.75 | | In the Mpumalanga Men’s study (2012-2013), 13.6% (95%CI: 7.8-21.5) and 9.7% (95% CI: 3.6-19.9) of MSM testing HIV positive in Gert Sibande and Ehlanzeni, respectively, self-reported currently taking ART (Lane, Osmand et al. 2014). In the Sibanye Health Project (2015), 11.4% (95%CI: 6.6-19.1) and 27.8% (95% CI: 22.7-33.5) of MSM testing HIV positive in Cape Town and Port Elisabeth, respectively, self-reported currently taking ART. In the Sibanye Health Project (2015), 22.9% (95%CI: 15.8-31.9) and 22.1% (95% CI: 17.4-27.6) of HIV +ve MSM were virally supressed in Cape Town and Port Elisabeth, respectively. In HPTN 075 (2015/16), ARV drugs were detected in 27.4% (95%CI: 18.2-38.2) and 25.0% (95%CI: 13.2-40.3) HIV positive MSM in Soweto and Cape Town, respectively (Zhang, Fogel et al. 2018).  UNAIDS give an ART coverage among men of 32% (23-40) in 2012 and 43% (32-54) in 2015; so we assume MSM have ART coverage of 0.3-0.75 that of all males. |
| Proportion of MSM that are circumcised. | $\zeta$ | Time varying | Not applicable. | See section on circumcision trends. |

## Supplementary Table 6. HIV epidemiological parameters

| Parameter | Range | Reference |
| --- | --- | --- |
| HIV transmission probability per receptive vaginal sex act | 0.0006-0.006 | (Boily, Baggaley et al. 2009, Hughes, Baeten et al. 2012) |
| Relative risk of acquiring HIV from receptive VI vs. insertive VI | 1-3 | (Boily, Baggaley et al. 2009) |
| Relative risk of acquiring HIV from receptive AI vs. receptive VI | 2.0-18.0 | (Boily, Baggaley et al. 2009, Baggaley, White et al. 2010) |
| Relative risk of acquiring HIV from insertive AI vs. insertive VI | 1.0-2.0 | (Boily, Baggaley et al. 2009, Baggaley, White et al. 2010) |
| Relative infectiousness of those in the acute stage of HIV infection compared with the chronic stage | 4.47-18.8 | (Boily, Baggaley et al. 2009) |
| Relative infectiousness of those in the pre-AIDS and AIDS stages of HIV infection compared with the chronic stage | 4.45-11.88 | (Boily, Baggaley et al. 2009) |
| Efficacy of ART in reducing HIV transmission risk. | 0.70-0.90 | From a summary of studies that reported proportion virally suppressed on ART in South Africa. (Barth, Tempelman et al. 2011, Barnabas, van Rooyen et al. 2014, Fatti, Mothibi et al. 2014, Shearer, Brennan et al. 2014, Abuelezam, McCormick et al. 2016, Jean, Puren et al. 2016, Johnson, Chiu et al. 2016, Kapiamba, Masango et al. 2016, Lecher 2016, Lippman, Shade et al. 2016, Moyo, Chasela et al. 2016, Coetzee, Hunt et al. 2017, Huerga, Shiferie et al. 2017, Tsondai, Wilkinson et al. 2017, Zanoni, Sibaya et al. 2017) |
| Duration of acute stage in years | 0.10-0.38 | (Hollingsworth, Anderson et al. 2008) |
| Duration of pre-AIDS stage in months when not on ART | 4.8-14.0 | (Hollingsworth, Anderson et al. 2008) |
| Duration of AIDS stage in months when not on ART | 6.9-12.7 | (Hollingsworth, Anderson et al. 2008) |
| ART recruitment rate | Varied | Varied to fit ART coverage in the different age groups. |
| Average time to death when not on ART per year | 8.7-14.2 | (Todd, Glynn et al. 2007) |
| Factor difference in HIV related death when on ART compared to off ART | 0.1-0.45 | Systematic search showed that HIV patients with CD4 counts 200-350 can expect to live 4.8 life years without treatment but have an expected net benefit of 14.5 life years on treatment (Johansson, Robberstad et al. 2010)  Crude mortality rate for individuals who started cART and followed up for a median of 48 months and 24 months for different years of enrolled was 31.8 (95%CI: 30.3-33.2) per 1000 pyrs (Mills, Bakanda et al. 2011). |
| Loss to follow up on ART for general population per year | 6.4-18.2% | Of those who initiated ART in < 90 days 8% (15/190) in the standard arm group were lost to follow up at 10-month visit (Rosen, Maskew et al. 2016). Therefore, the loss to follow up from those in the standard arm was 11.3%(6.4-18.2%) per year.  In a clinic in Gauteng Province, South Africa, a retrospective observational cohort showed incidence of loss to follow up of 109 per 1000 person-years (95%CI: 92-128) in general population (Mberi, Kuonza et al. 2015). We take the lower and upper bound from the two studies. |
| Loss to follow up on ART for MSM per year | Assume the same LTFU rate for MSM as the general population | A study in Johannesburg found there were no statistically significant differences in ART retention between MSM and other men. (Rees, Radebe et al. 2017) |
| Loss to follow up on ART for FSW per year | 30.2% (22.7-38.6%) | The TAPs study among FSW showed that loss to follow up on early ART was 30.2% (22.7-38.6%) in the first 12 months out of 139 FSW enrolled onto ART (Eakle, Gomez et al. 2017) |
| Efficacy (%) of circumcision for reducing HIV susceptibility among men | 54% (38-66%) | (Siegfried, Muller et al. 2009) |
| Per-sex-act (%) efficacy of condoms in reducing HIV transmission risk in vaginal and anal sex | 80% (66-94%) | (Weller and Davis 2001) |
| Non-HIV death rate – depending on gender. | Varied -time dependant | (United Nation, Department of Economic and Social Affairs et al. 2015) |

## Condom use assumptions

*Low risk male and female – main partners*

Analyses of 3 national studies show that condoms were used by ~6-10% of individuals at sexual debut during the 1980s(Kincaid, Babalola et al. 2014). A study among women attending antenatal clinic in Free state found that condoms were worn with 15.1% of all partners (RHRU 2000). Another study of women attending a family planning clinic in Gauteng had respondents reporting condom use with 44.8% of all partners in the last 4 weeks (RHRU 2000). Men attending clinics in Johannesburg reported 17.5% and 14.4% used with main and regular partners in the last 4 weeks (RHRU 2000). Another study among a rural adult population in South Africa in 2003 showed that 23.1% used condom in their last sex act with a regular partner (Peltzer, Mashego et al. 2003). Condom use in last sex with regular partner was 38.8-43.7% and 33.3-40.5% in the 2005 (Human Sciences Research Council 2005) and 2008 (Human Sciences Research Council 2008) HSRC surveys for males and females, respectively. Quaife et al., 2016 reports condom use of 61.6-74.7% for males and 36.0-50.0% for females with regular partners in the last sex act although the male data seems too high. In the model, we assumed condom use for VI in the low risk population with main partners increased from 0-10% in 1985 to 7.5-23% in 2000, then 16-50% by 2008, and remaining constant thereafter. We assumed half lower bounds in the trend (as given) to account for possible reporting bias and assumed same condom use for males and females.

Condom use for AI among main partners is assumed to be the same as that for VI for these partnership types. A recent systematic review found that in South Africa, condom use during AI was similar to that for VI; among general‐risk populations, the fraction of AI and VI acts that were unprotected was 27.0–53.6% and 26.9–57.0%, respectively (Owen, Elmes et al. 2017).

*Low risk male and female – casual partners*

Early on, men attending STI clinics in Johannesburg reported 8.9% condom use with casual partners in the last 4 weeks (RHRU 2000), while women attending a family planning clinic in Gauteng reported condom use with 44.8% of all partners in the last 4 weeks (RHRU 2000). More recently, males and females report condom use in last VI with casual partner of 82.1% (69.4-94.6%) and 75.0% (36.3-100%), respectively (Quaife, Eakle et al. 2016). In 2005, condom use with casual partners for male and female was 60.1-72.2% and 30.7-47.9%, respectively (Human Sciences Research Council 2005), whereas it was 20.2-59.4% and 43.7-78.9%, respectively, during the 2008 survey (Human Sciences Research Council 2008). For casual partners, the model assumes that condom use for VI increases to 4.5-45% in 2000, assuming a similar scale-up as for main partnerships over 1985-2000, and to 10.1-78.9% by 2005, remaining constant thereafter. The Quaife data was assumed to not be representative. We incorporated bias by incorporating half the lower bound in these estimates.

As for main partnerships, condom use for AI among casual partners is assumed to be the same as that for VI for these partnership types based on a recent systematic review (Owen, Elmes et al. 2017).

*FSW and clients – commercial sex*

A cross sectional survey carried out among FSW between 1996 and 1997 in Johannesburg, SA (Dunkle, Beksinska et al. 2005) reported that 34.6-65.6% of the FSW used condom with commercial partners all the time and about 50% in 50-75% of the time. A cross sectional survey of FSW in a semi-urban area of South Africa in Tzaneen and Phalaborwa, Limpopo Province carried out between 2001/02 showed that 41.1-80% of FSW used condoms with paying clients every time (Peltzer, Seoka et al. 2004). A national survey in 2008 found that condom use reported by males was 64.7% (38.3-85.8%) and females was 38.5% ( 20.2-59.4%) in their last sex act with commercial partners (of those who reported commercial partners in the last 12 months) (Human Sciences Research Council 2008). Recent FSW surveys have shown that condom use is now high with commercial partners with 93.0-98.6% of FSW reporting always using condoms with clients (Quaife, Eakle et al. 2016), 84.7-87.7% using condoms at last paid sex act (SAHMS-FSW 2014) and 83.9-98.8% using condoms with new and regular clients in last sex act (PEFSW 2015, Rao, Baral et al. 2016). In a recent (2017-2018) survey in Port Elisabeth, 72.9% (95%CI: 69.0-76.5) and 43.0% (95% CI: 33.2-53.4) of clients reported using a condom at last commercial VI and AI act, respectively. In a recent (2018) survey in Klerksdorp, 91.6% (95%CI: 84.6-96.1) of clients reported using a condom at last commercial sex act. In the model, for VI, we assumed an initial condom use of 0-20% between 1985 and 1990, increasing to 30-90% in 1997 (lower bound from Dunkle data - 34.6%+50%*50% which is 60% and then halved because of likely reporting bias), then to 30-98.8% in 2008 and constant thereafter based on FSW and client surveys and data from general population survey in 2008. We also assumed that condom use did not decrease over this period. For anal sex, we assumed 0.5-1 times the level of condom use as VI over time due to client data and data that 43% (30.2-56.8%) of FSW (n=58) (PEFSW 2015, Rao, Baral et al. 2016) reported condom use at last AI with new clients and 82% (66.5-92.5%, n=39) reported condom use at last AI with regular clients – these are lower than the condom use estimates these FSW report for VI in commercial sex.

*FSW and clients – non-commercial sex*

Data suggests condom use of FSW with main partners was very low in 2000 (5-15%) (RHRU 2000/01), with another survey having 66% of FSW reporting not using a condom with the last non-paying partner in 2001/02 (Peltzer, Seoka et al. 2004). A moderate condom use was reported by FSW in 2013 (SAHMS 2013) of 33.7-41.7% in last vaginal sex act and another FSW survey had condom use of 26.3-44.1% for VI with long term partners (PEFSW 2015, Rao, Baral et al. 2016). In the model, we therefore assumed that condom use during VI with main partners was 2.5-33% in 2000, assuming a similar scale-up to condom use among low-risk females over 1985-2000, increasing to 13-44.1% by 2013 and remaining stable thereafter. We halved the lower bound to account for possible reporting bias. Based on levels of condom use for FSW with their main (26-44%) and casual (37-64%) partners from a recent FSW survey (PEFSW 2015), we assume condom use for casual partners is 1.25-1.75 times that of main partners. For anal sex with main partners, all 14 FSW used condom for last anal sex with main partner in one survey (Quaife, Eakle et al. 2016), whereas out of the 10 FSW who responded in another survey, only 1 used condom during last AI with their long term partner (PEFSW 2015). Data is very limited, so in the model we assumed the same level of condom use for anal sex as for vaginal sex for casual and main partnership sex acts.

In a recent (2017-2018) survey in Port Elisabeth, clients report using condoms in 34.1% (95%CI: 29.5-38.7%) and 73.2% (95%CI: 68.0-78.3) of their vaginal sex acts with main and casual partners in the past 30 days, respectively. In a recent (2018) survey in Klerksdorp, clients report using condoms 37.0% (95%CI: 19.4%-57.6%) and 76.6% (95%CI: 64.3-86.2) at their last sex act with main and casual partners, respectively. We take the upper and lower bounds of these estimates and incorporated bias by incorporating half the lower bound in these estimates. For clients’ condom use with main and casual partners, we assume that condom use is scaled up to 9.7-57.6% and 32.15-86.2, respectively, following the same trend as for low risk males. As was the case for FSW, data is very limited, so in the model we assumed the same level of condom use for anal sex as for vaginal sex for casual and main partnership sex acts; only 17 and 18 participants provide data on condom use for anal sex with main and casual partners, respectively.

*MSM – male main and casual partnerships*

In 2004-5, 58.5% (95% CI: 50.1-66.6) of MSM in Gauteng province reported any unprotected anal sex in the past 6 months(Lane, Shade et al. 2008). In 2008, MSM in Soweto, Johannesburg, reported that amongst male partners in the past 6 months, unprotected anal intercourse typically accounted for 24% of same-sex anal intercourse sex acts with a given partner (Arnold, Struthers et al. 2013). Another 2008 study found similarly low levels of condom use among MSM in Pretoria, where 33% (95% CI: 27.5-38.9) of MSM reported using condoms during the last anal sex act (Knox, Reddy et al. 2013). In 2008, 51.5% (95%CI: 45.6-57.5) of MSM participating in the Johannesburg/eThekwini Men’s Study reported any unprotected anal sex in the past year (Rispel, Metcalf et al. 2011). In 2009, 6.5% of MSM in Cape Town reported consistent condom use with all sexual partners, 52.4% reported always wearing condoms with male sexual partners and 39.5% reported always wearing condoms with female sexual partners (Baral, Burrell et al. 2011). In 2010, 37.8% (95%CI: 33.6-42.1%) of internet using MSM (recruited on Facebook) reported using a condom at last sex act (Stephenson, de Voux et al. 2011). In another 2010 study, 55.2% of MSM in Cape Town reported any unprotected anal sex in the last 6 months (Tucker, Liht et al. 2013, Tucker, Liht et al. 2014). In 2012, 62% of a small sample of MSM in Cape Town and Port Elisabeth (n=34) reported always using condoms with their most recent anal sex partner (Siegler, Voux et al. 2014). In 2011-13, 47.1% (95% CI: 42.5-51.7) of MSM reported unprotected anal sex in the past 3 months (Knox, Reddy et al. 2017).

Studies conducted from 2012/13 onwards consistently find high levels of condom use at last sex act. In the Mpumalanga Men’s study (2012-2013), 85.1% (95% CI: 78.2-90.7) and 63.7% (95%CI 55.9-70.1) of MSM in Gert Sibande and Ehlanzeni, respectively, reported consistent condom use with their last 5 male partners (Lane, Osmand et al. 2014). In the South Africa Marang Men’s project (2012-13), 80.7% (95%CI:74.0-88.1), 84.6% (95%CI: 78.8-89.2), and 80.5% (95%CI: 74.2-86.7) of MSM in Durban, Cape Town and Johannesburg, respectively, reported condom use in the last sex act (Cloete, Jooste et al. 2014). In 2014-2015, an internet survey of MSM found that 60.0% (95%CI: 55.1-64.8) of MSM reported unprotected anal sex in the past 6 months (Hugo, Stall et al. 2016). In the Sibanye Health Project (2015), 80.2% (95%CI: 71.8-88.7%) and 69.5% (95%CI: 61.1-77.9%) of MSM in Cape Town and Port Elisabeth, respectively, reported using condoms in the last sex act with a main male partner. In the same survey, similar rates of condom use were reported for casual partners; 76.9% (95%CI: 67.5-86.2) and 80.3% (95%CI: 73.5-87.3) of MSM in Cape Town and Port Elisabeth, respectively, reported using condoms) in the last sex act with a casual male partner. Hence, we assume that condom use for homosexual sex is the same for sex with main and casual partners. We incorporated bias by halving the lower bound in all these estimates. In the model we assume that condom use between MSM is scaled up to 12-54.4% in 2008, following the same trend as low-risk men over 1985-2008, and increases to 28-90.7% in 2014. Condom use is then assumed to remain stable.

*MSM and female partners – main and casual partnerships*

Little data is available on condom use between MSM and their female partners. Estimates from the Sibanye Health Project (2015) suggest that MSM in Port Elisabeth and Cape Town have high condom use with their female partners: 71.4% (n=7; 95%CI: 21.5-95.8) and 59.3% (n=27; 95%CI: 39.0-76.8) of MSM report condom use at their last anal and vaginal sex with a main female partner and 76.2% (n=9; 95%CI: 51.7-90.5) and 77.8% (n=21; 95%CI: 33.0-96.1%) of MSM report condom use at their last anal and vaginal sex with a casual female partner. We therefore assume that condom use for MSM with their female partners is the same as with their male partners.

## Male circumcision coverage assumptions

The model assumes 38.0% (32.4-42.0%) of men are circumcised between 1985-2002 because of tribal groups like the Xhosa who carry out traditional circumcision (Mayatula and Mavundla 1997, Human Sciences Research Council 2002, Meissner and Buso 2007). It was assumed to increase from 2002 to 41% (36.8-43.3%) by 2008 (Human Sciences Research Council 2008), to 46.4% (44.1-48.8%) by 2012 (Human Sciences Research Council 2012) and to 61.6% (59.3-63.9) by 2017 (Human Sciences Research Council). We assume stable thereafter. We assume that all male risk groups (i.e. low risk males, MSM and clients) have the same levels of circumcision.

# Model Calibration

The model was calibrated using an approximate Bayesian computation Sequential Monte Carlo (ABC SMC) method(Toni, Welch et al. 2009), which accounts for uncertainty in the calibration data and parameters and ranks different model runs by their goodness of fit. The ABC method was used to calibrate the model to:

- total population and KP size estimates (FSW in 2013, MSM in 2005)
- ART coverage levels among all adults in 2010 and in different sub-groups in 2018
- HIV prevalence estimates for adult males and females (15-49 years) from the HSRC surveys (2005, 2012 and 2017), FSWs in 2000 (38.6-58.8%) and 2016 (45.5-68.0%); young MSM in 2009 (13.2-56.5%) and all MSM in 2016 (20.0-58.2%).
- HSV-2 prevalence estimates for HIV negative males in 2014 (13.0-32.1%), HIV positive males in 2014 (68.7-88.4%), HIV negative females in 2008.5 (37.4-67.6%) and HIV positive females in 2014 (83.0-94.2%).

Calibration ranges were defined for each estimate to be the 95% confidence interval (CI) of an estimate. For HSV-2 prevalence by gender and HIV status and HIV prevalence among FSW and MSM, where there were multiple estimates at similar timepoints, the calibration range was defined to be the minimum lower 95%CI to the maximum upper 95%CI across these estimates. Goodness of fit for each parameter set was then defined as the sum of the absolute relative differences (defined as 0 if model projections lie within the calibration range) between the calibration data and the corresponding model projections.

The relative risk of HIV acquisition due to HSV-2 during non-GUD periods was assigned a uniform prior and calibrated based on differences in HSV-2 prevalence by HIV status. The relative additional risk of HIV acquisition due to HSV-2 during GUD periods compared to non-GUD periods was assigned a conservative prior of 1-3. The resulting modelled increase in HIV incidence by HSV-2 status was compared to pooled adjusted estimates of the incidence rate ratio (IRR) from a recent systematic review (aIRR: 2.5; 95%CI 1.8-3.4 among females and 3.1, 95%CI 2.2-4.3 among males)(Looker, Elmes et al. 2017). Based on previous analyses, we assumed that the relative HIV transmission risk if co-infected with HSV-2 compared to HIV mono-infection is 1.33 (1.00-1.93)(Silhol, Coupland et al. 2021). Using a recent systematic review(Kelly and Mayaud 2019), we assumed that untreated PLHIV have greater odds of asymptomatic (2.16; 95%CI: 1.09-4.28) and symptomatic (2.78; 95%CI: 1.51-5.14) HSV-2 shedding compared to HIV-uninfected individuals, with ART mitigating these increases (OR: 0.56, 95%CI: 0.41-0.77).

The ABC SMC begins with 5,000 parameter sets sampled from prior distributions using Latin Hypercube sampling, which are then successively perturbed to improve their goodness of fit. At each subsequent iteration, parameter sets are resampled from the previous iteration and perturbed, until 5,000 parameter sets are obtained iteratively so that which fit the data at least as well as the best 50% parameter sets from the previous iteration. The ABC continues each time the parameter sets better fit the data, until successive iterations no longer improve the goodness of the fits. The ABC routine produced a set of 5,000 baseline model fits which were used to give the median and 95% credibility intervals (95%CrI; 2.5^th^ to 97.5^th^ percentile range) for all model projections.

# Supplementary Results


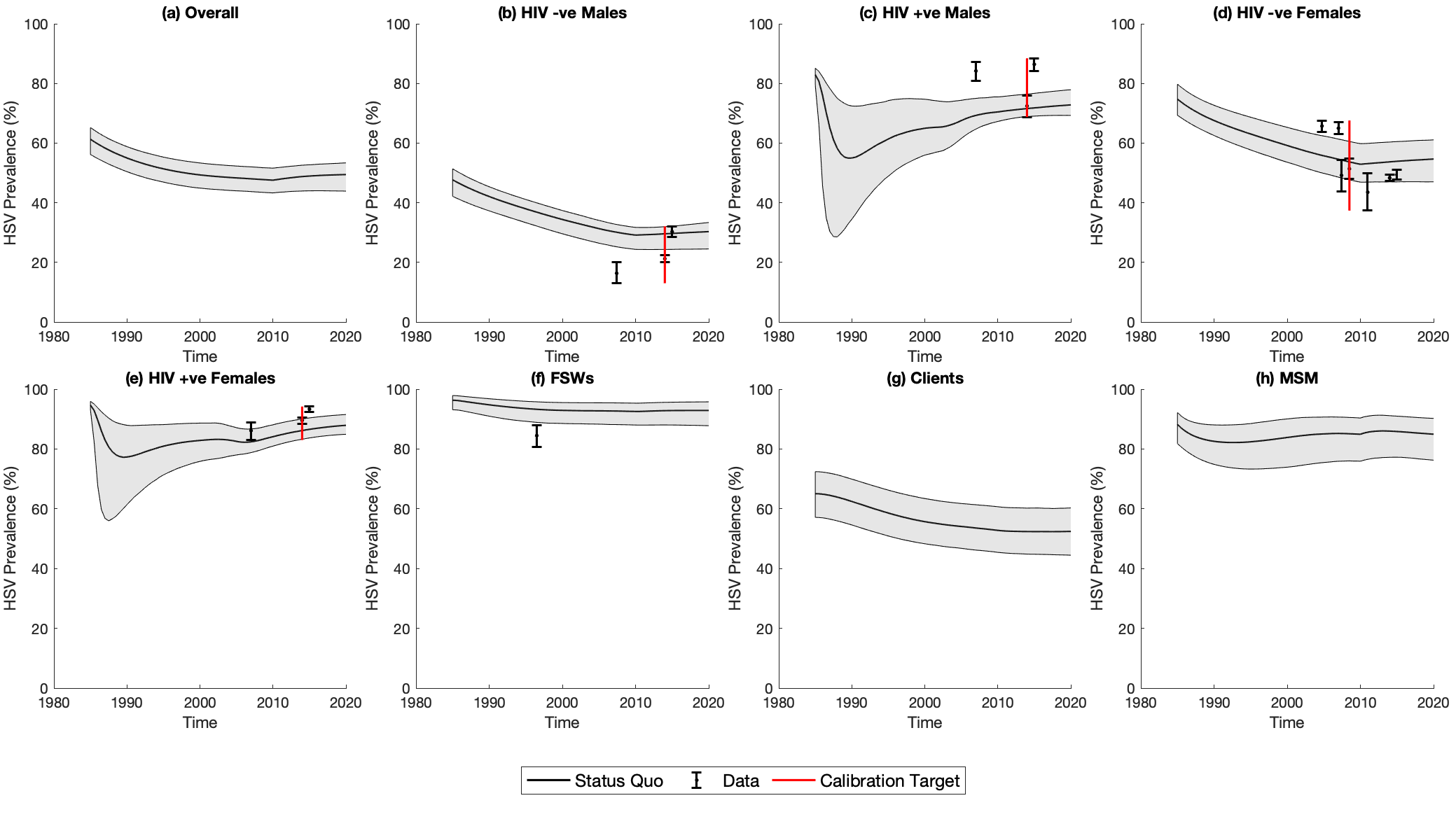


**Supplementary Figure 3:** A comparison of median and 95% credibility intervals from baseline model fits (black line and shaded area) with HSV-2 prevalence estimates for (a) overall adult population, (b) HIV negative adult male population, (c) HIV positive adult male population, (d) HIV negative adult female population, (e) HIV positive adult female population, (f) female sex workers (FSWs), (g) their clients, and (h) men who have sex with men (MSM). Red points and whiskers show data with 95% confidence intervals used for model calibration, and black points show cross validation data not used in model calibration but shown to compare with model projections.


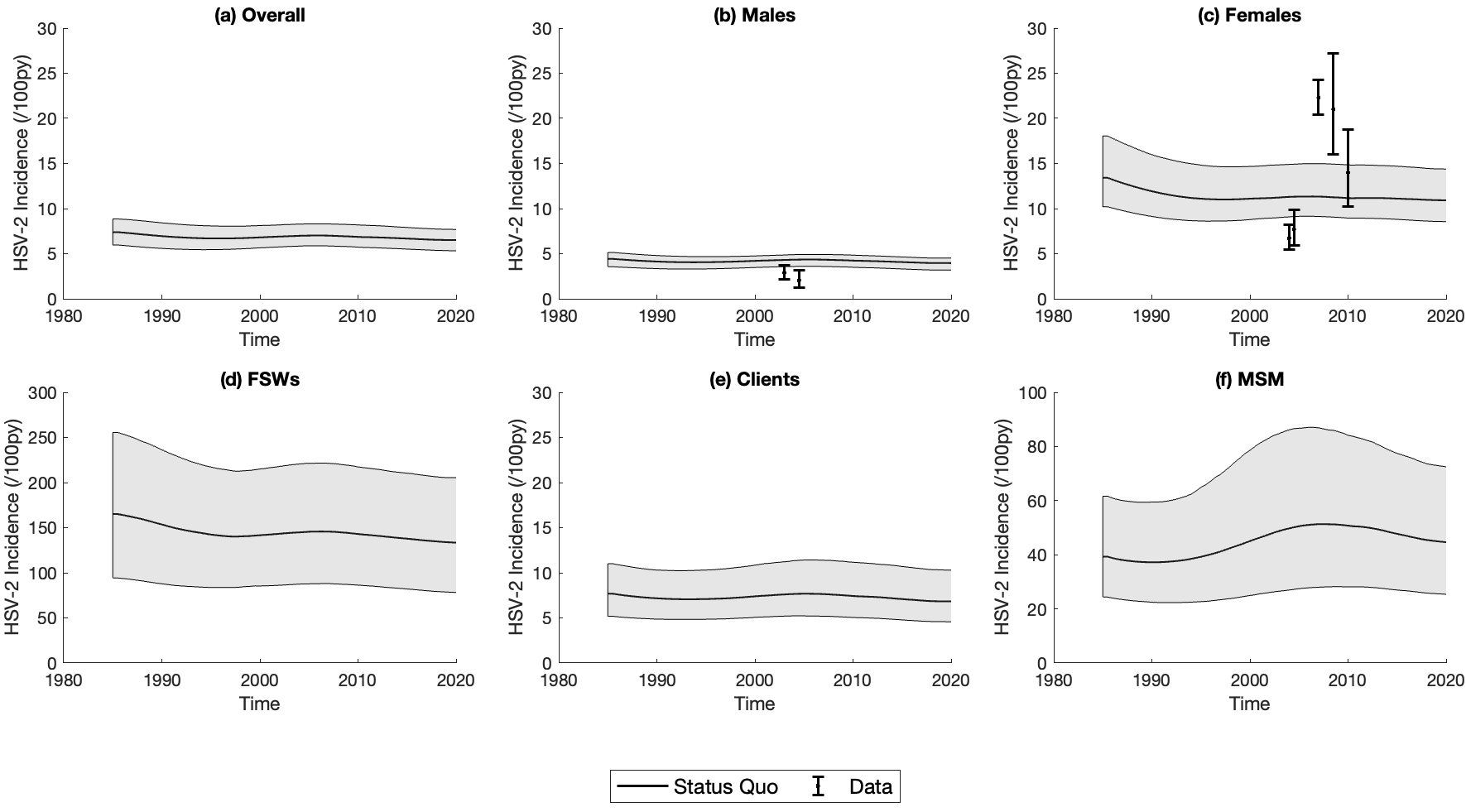
**Supplementary Figure 4:** A comparison of median and 95% credibility intervals from baseline model fits (black line and shaded area) with HSV-2 incidence estimates for (a) overall adult population, (b) adult male and (c) adult female general population, (d) female sex workers (FSWs), (e) their clients, and (f) men who have sex with men (MSM). Black points show cross validation data not used in model calibration but shown to validate the model projections. Note that the scale for the y-axes are different for figures (d) and (f).


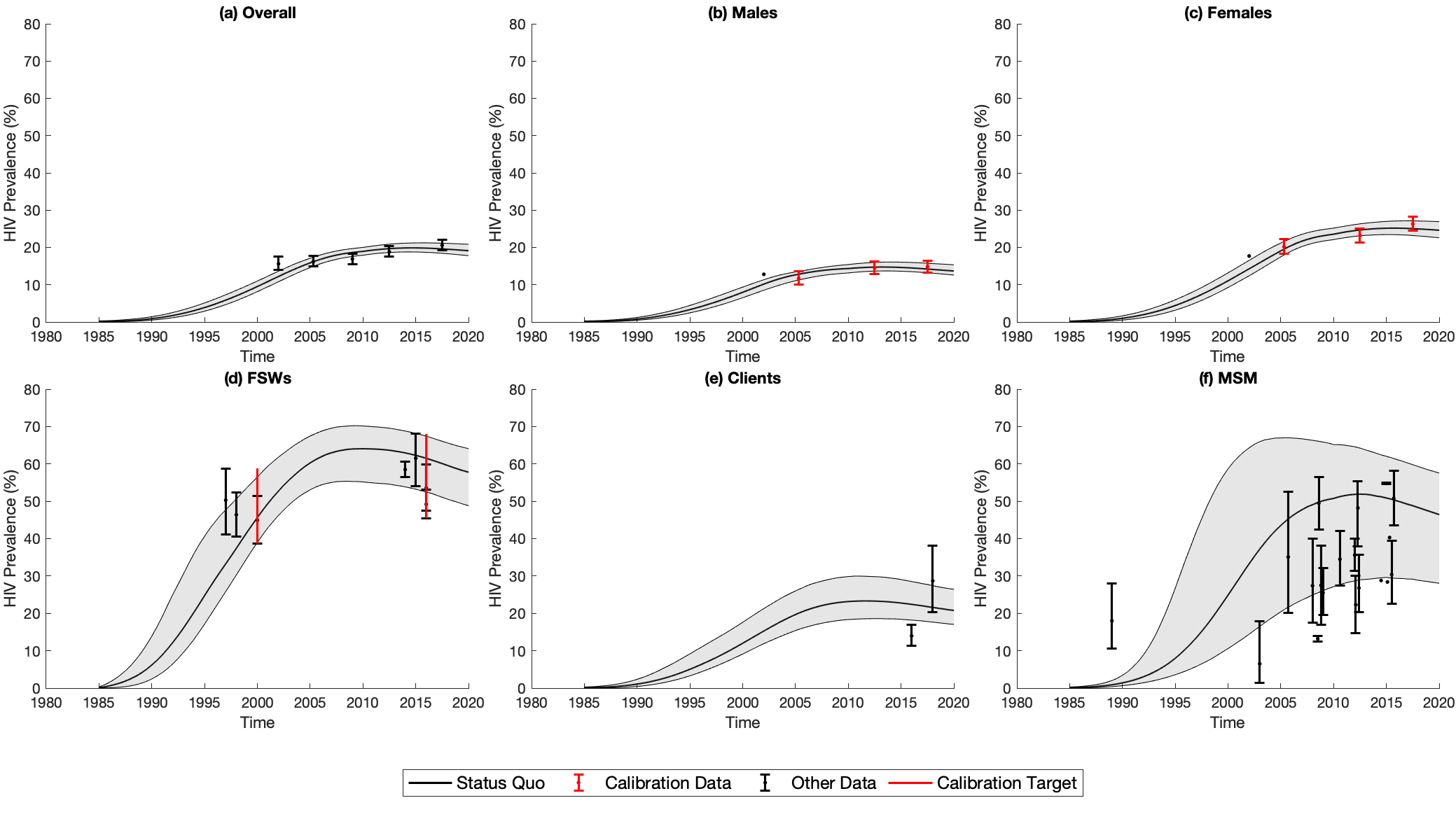
**Supplementary Figure 5:** A comparison of median and 95% credibility intervals from baseline model fits (black line and shaded area) with HIV prevalence estimates for (a) overall adult population, (b) adult male and (c) adult female general population, (d) female sex workers (FSWs), (e) their clients, and (f) men who have sex with men (MSM). Red points and whiskers show data with 95% confidence intervals used for model calibration, and black points show cross validation data not used in model calibration but shown to compare with model projections.


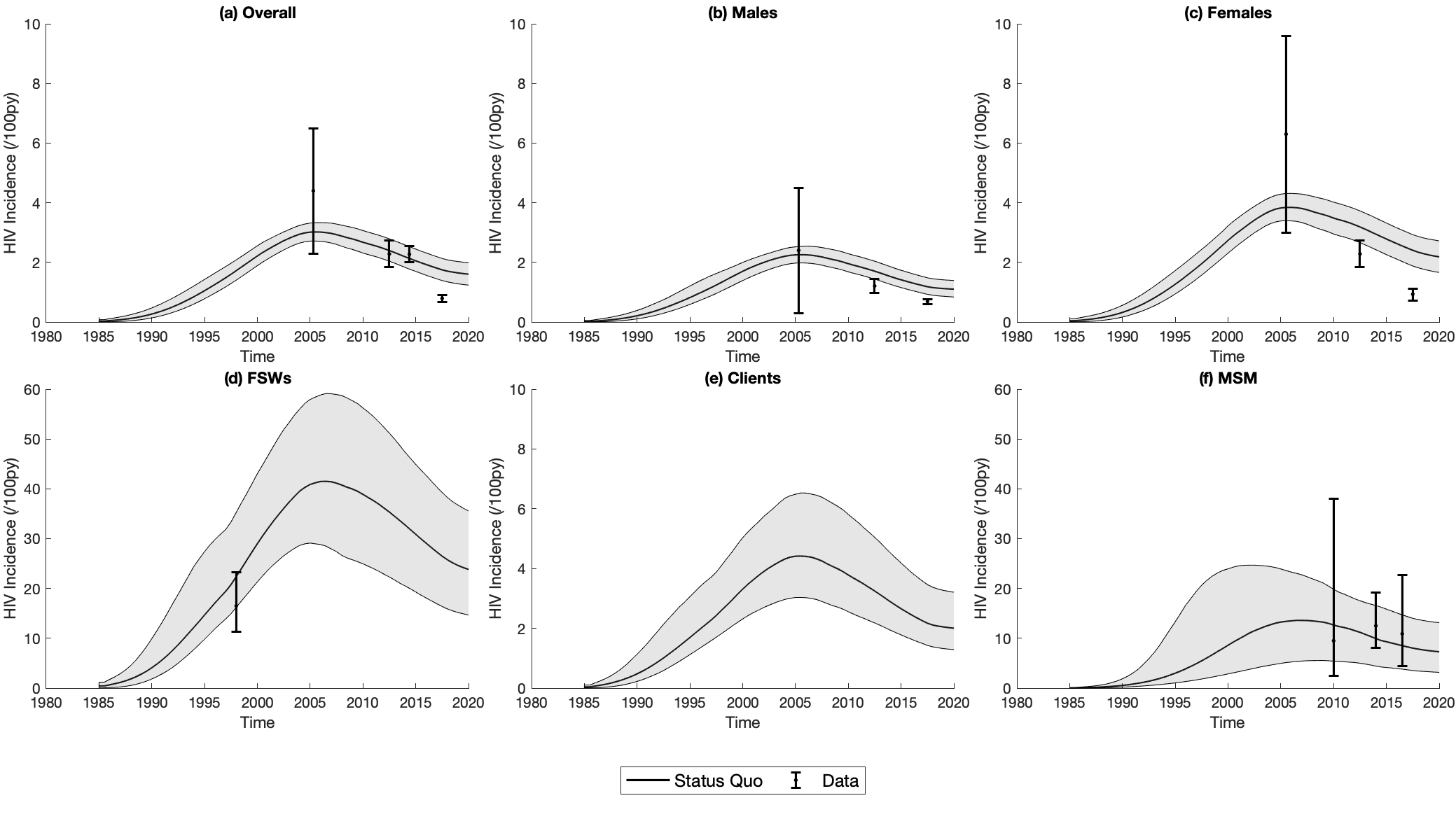
**Supplementary Figure 6:** A comparison of median and 95% credibility intervals from baseline model fits (black line and shaded area) with HIV incidence estimates for (a) overall adult population, (b) adult male and (c) adult female general population, (d) female sex workers (FSWs), (e) their clients, and (f) men who have sex with men (MSM. Black points show cross validation data not used in model calibration but shown to validate the model projections. Note that the scale for the y-axes are different for figures (d) and (f).


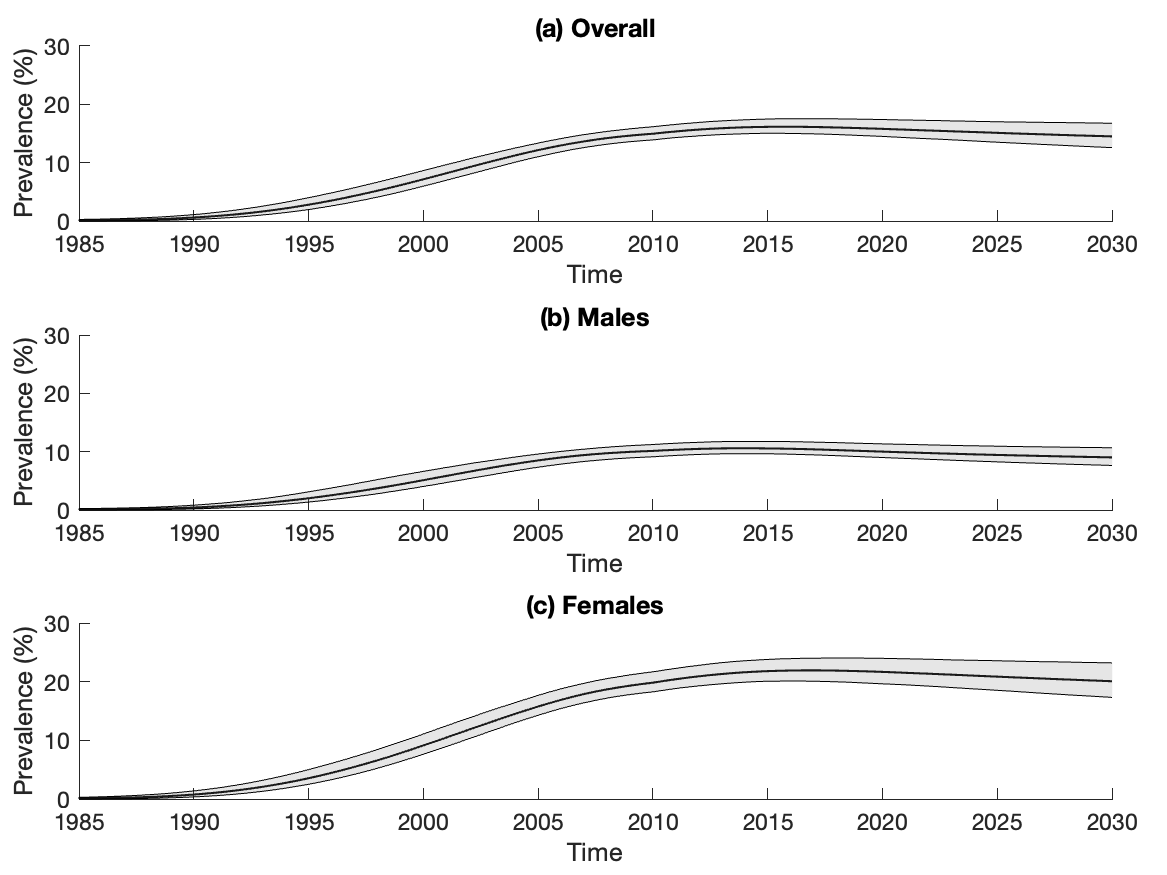


**Supplementary Figure 6:** Baseline model projections of the prevalence of HIV-HSV-2 coinfection among the (a) overall adult population, (b) adult male population and (c) adult female population. The black line shows the median projections and the shaded area give the 95% credibility intervals.


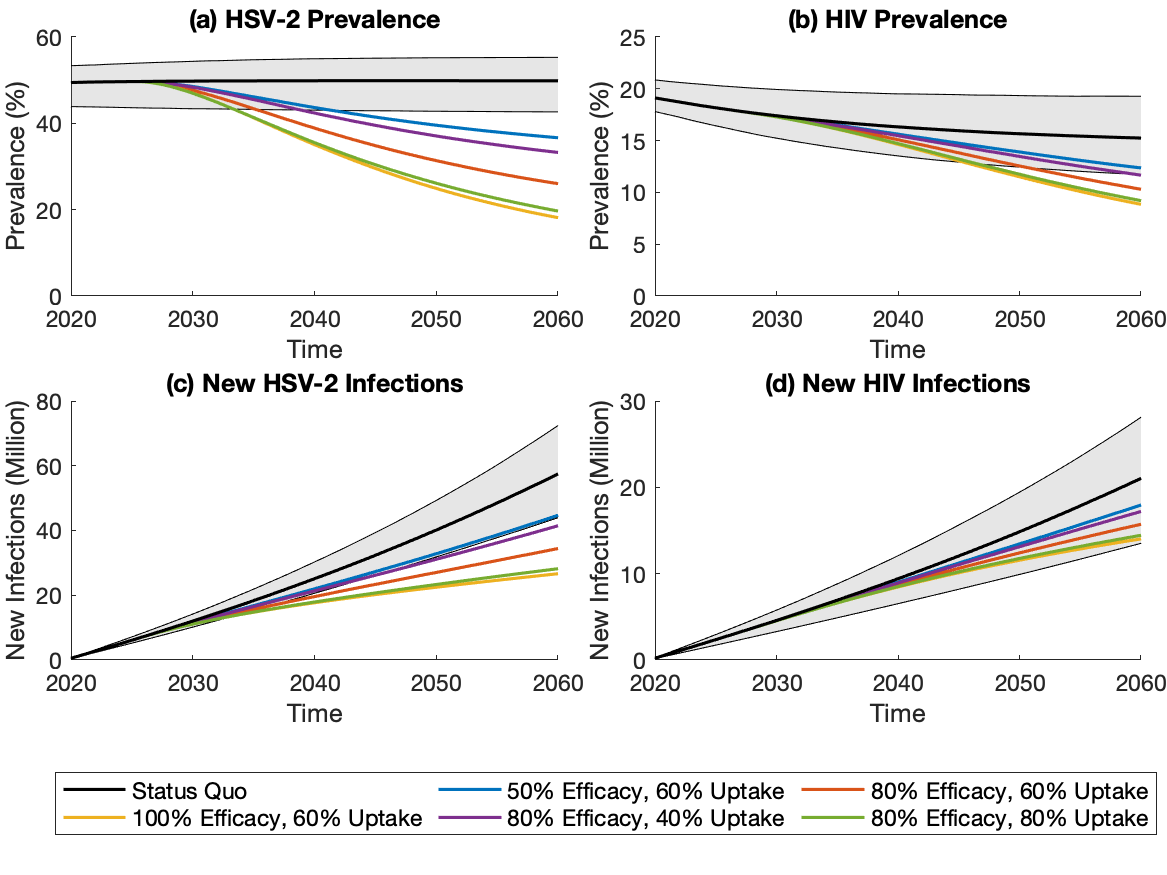


**Supplementary Figure 8:** Model projections of the impact of a prophylactic vaccine on (a) HSV-2 prevalence among 15-49 year olds; (b) HIV prevalence among 15-49 year olds; (c) cumulative number of new HSV-2 infections (d) cumulative number of new HIV infections. Coloured lines show median projections for vaccinating a proportion (the uptake) of 9 year-olds each year with a prophylactic vaccine which has lifelong protection and provides protection against HSV-2 acquisition. Black lines and grey shading area show the median and 95%CrI for the status quo scenario.

**Supplementary Figure 9: Sensitivity analyses for the 20-year impact of prophylactic vaccination**. Figures show how the impact of a prophylactic vaccine would differ in each sensitivity analysis compared to the baseline vaccination scenario (shown in bold): vaccinating 60% of 9 year-olds each year with a prophylactic vaccine which has lifelong protection and provides degree type protection against HSV-2 acquisition with 50% (blue), 80% (green) or 100% (red) efficacy. Impact measured as relative reduction by 2040 in (a) HSV-2 incidence, (b) annual GUD days, (c) HIV incidence. Dashed lines show the median projections for the baseline vaccination scenarios.


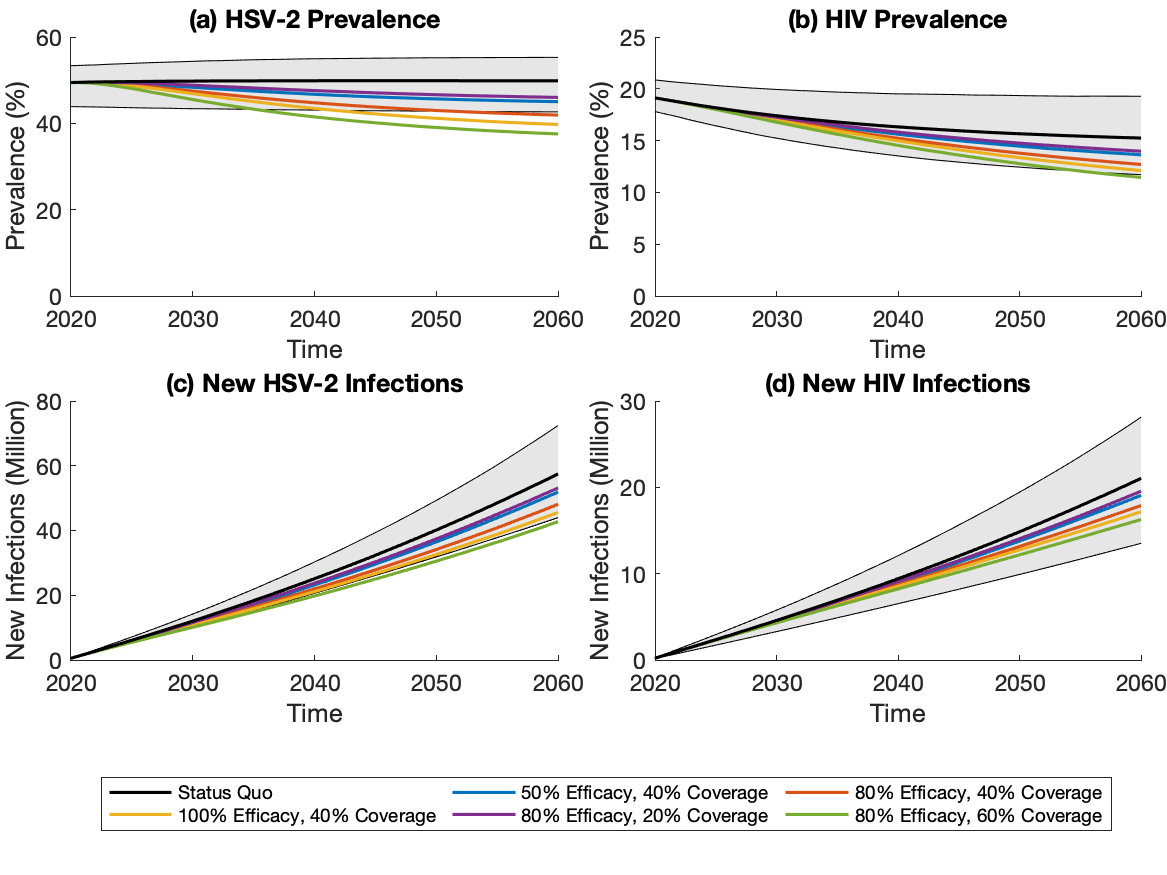


**Supplementary Figure 10:** Model projections of impact of a therapeutic vaccine which has lifelong protection on (a) HSV-2 prevalence among 15-49 year olds; (b) HIV prevalence among 15-49 year olds; (c) cumulative number of new HSV-2 infections (d) cumulative number of new HIV infections. Projections show median projections for vaccinating symptomatic adults each year with a therapeutic vaccine which has lifelong protection and reduces days with asymptomatic or symptomatic shedding. Black lines and grey shading area show the median and 95%CrI for the status quo scenario.

**Supplementary Figure 11: Sensitivity analyses for the 20-year impact of therapeutic vaccination**. Figures show how the impact of a therapeutic vaccine would differ in each sensitivity analysis compared to the baseline vaccination scenarios vaccinating HSV-2 infected symptomatic individuals with a therapeutic vaccine to a coverage of 40% after 40 years which has lifelong protection and reduces asymptomatic and symptomatic shedding with 50% (blue), 80% (green) or 100% (red) degree type efficacy. Impact measured as relative reduction by 2040 in (a) HSV-2 incidence, (b) annual GUD days, (c) HIV incidence.


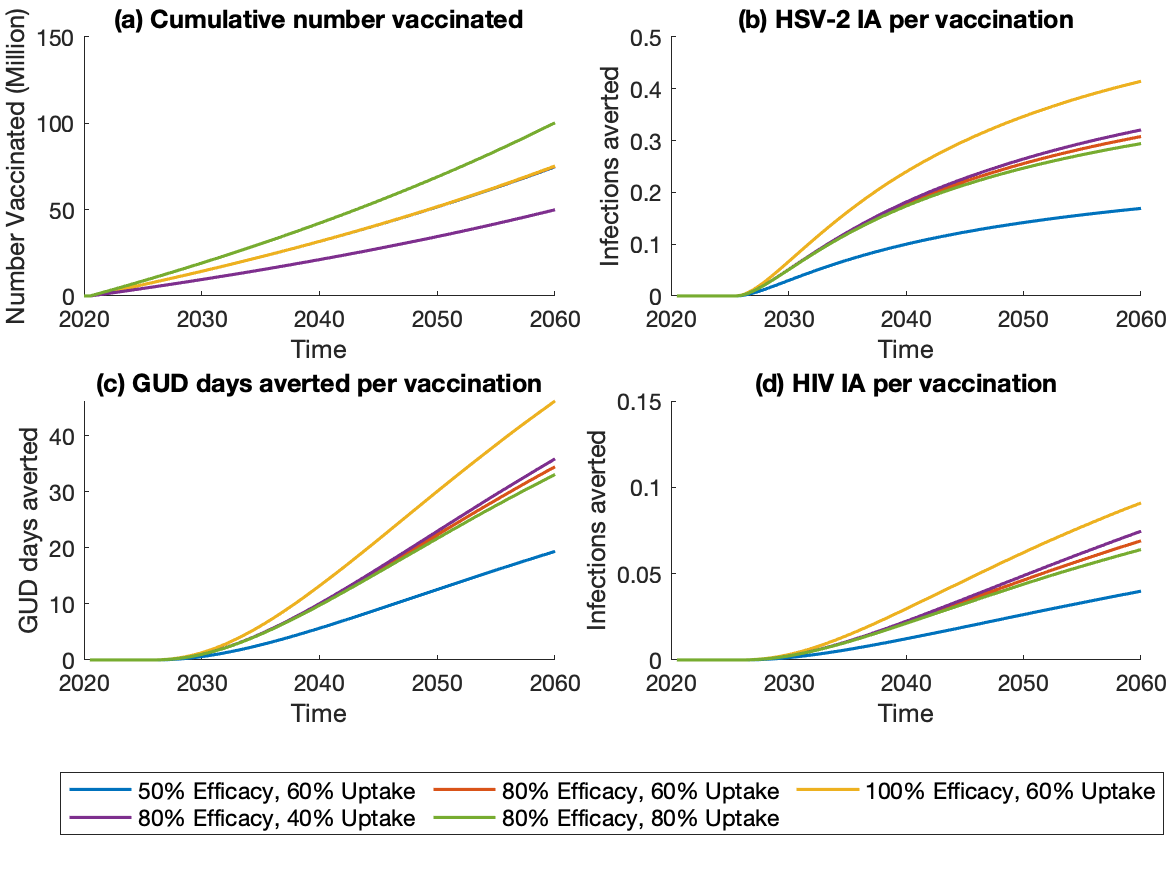


**Supplementary Figure 12:** Additional model projections for prophylactic vaccination: (a) cumulative number of individuals vaccinated; (b) HSV-2 infections averted per vaccination; (c) GUD days averted per vaccination; (d) HIV infections averted per vaccination. Coloured lines show median projections for vaccinating a proportion (the uptake) of 9 year-olds each year with a prophylactic vaccine which has lifelong protection and provides protection against HSV-2 acquisition.


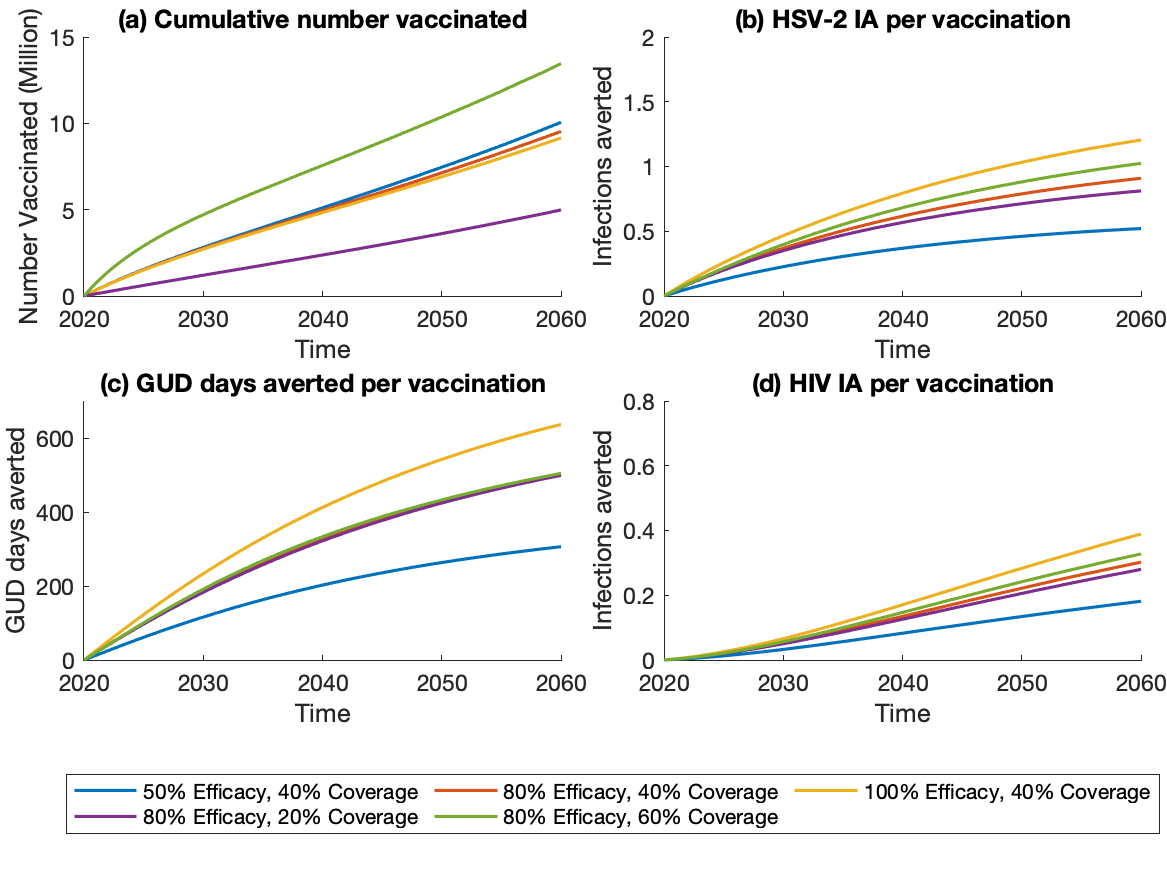


**Supplementary Figure 13:** Additional model projections for therapeutic vaccination: (a) cumulative number of individuals vaccinated; (b) HSV-2 infections averted per vaccination; (c) GUD days averted per vaccination; (d) HIV infections averted per vaccination. Projections show median projections for vaccinating symptomatic adults each year with a therapeutic vaccine which has lifelong protection and reduces days with asymptomatic or symptomatic shedding.

**Supplementary Table 7.** Model projections of the impact of a prophylactic HSV-2 vaccine after 40 years. Results show the impact of vaccinating a proportion (the uptake) of 9 year-olds each year from 2020 with a prophylactic vaccine which has lifelong protection and provides protection against HSV-2 acquisition. Table shows median values with 95% credibility intervals in parentheses.

| **Uptake** | **Efficacy** | **Coverage among 15-49 y.o. (2060)** | **Relative reduction in HSV-2 incidence (2060)** | **Relative reduction in HIV incidence (2060)** | **Relative reduction in number of GUD days (2060)** |
| --- | --- | --- | --- | --- | --- |
| 40% | 50% | 36.7% (35.4 - 37.3) | 33.4% (30.8 - 35.8) | 23.7% (18.7 - 27.8) | 17.7% (14.8 - 20.3) |
| 60% | 50% | 55.0% (53.0 - 55.9) | 46.4% (43.0 - 49.4) | 33.5% (26.8 - 38.8) | 26.1% (21.9 - 29.7) |
| 80% | 50% | 73.2% (70.7 - 74.4) | 57.4% (53.6 - 60.7) | 42.1% (34.1 - 48.1) | 34.1% (29.0 - 38.3) |
| 40% | 80% | 36.7% (35.4 - 37.4) | 56.1% (53.4 - 58.3) | 41.5% (34.2 - 46.9) | 32.9% (28.5 - 36.1) |
| 60% | 80% | 55.0% (53.1 - 55.9) | 72.5% (69.5 - 74.7) | 55.1% (46.6 - 61.2) | 46.7% (41.2 - 50.6) |
| 80% | 80% | 73.3% (70.7 - 74.5) | 84.1% (81.2 - 86.0) | 65.4% (56.5 - 71.6) | 58.8% (52.6 - 62.8) |
| 40% | 100% | 36.8% (35.5 - 37.4) | 71.5% (69.6 - 73.0) | 55.3% (47.7 - 61.4) | 45.7% (41.0 - 48.6) |
| 60% | 100% | 55.1% (53.1 - 56.0) | 86.8% (85.3 - 87.8) | 68.9% (61.0 - 75.1) | 62.0% (56.8 - 65.2) |
| 80% | 100% | 73.3% (70.7 - 74.5) | 95.4% (94.6 - 96.0) | 77.3% (69.8 - 83.0) | 74.5% (69.4 - 77.6) |

**Supplementary Table 8.** Model projections of the impact of a prophylactic HSV-2 vaccine after 40 years, by gender. Results show the impact of vaccinating a proportion (the uptake) of 9 year-olds each year from 2020 with a prophylactic vaccine which has lifelong protection and provides protection against HSV-2 acquisition. Table shows median values with 95% credibility intervals in parentheses.

| **Uptake** | **Efficacy** | **Relative reduction in HSV-2 incidence (2060)** | | **Relative reduction in HIV incidence (2060)** | | **Relative reduction in number of GUD days (2060)** | |
| --- | --- | --- | --- | --- | --- | --- | --- |
|  |  | **Adult Males** | **Adult Females** | **Adult Males** | **Adult Females** | **Adult Males** | **Adult Females** |
| 40% | 50% | 31.0% (28.2 - 33.6) | 38.6% (36.7 - 40.4) | 21.3% (16.1 - 25.9) | 25.5% (20.5 - 30.1) | 18.7% (15.6 - 21.1) | 17.1% (14.2 - 20.1) |
| 60% | 50% | 43.5% (39.8 - 46.9) | 52.2% (49.6 - 54.4) | 30.2% (23.2 - 36.3) | 35.9% (29.3 - 41.6) | 27.3% (23.0 - 30.6) | 25.4% (21.2 - 29.4) |
| 80% | 50% | 54.3% (50.0 - 58.2) | 63.1% (60.2 - 65.5) | 38.2% (29.6 - 45.3) | 44.8% (37.2 - 51.3) | 35.4% (30.1 - 39.4) | 33.4% (28.2 - 38.2) |
| 40% | 80% | 52.3% (48.8 - 55.3) | 62.1% (60.1 - 64.0) | 38.4% (30.6 - 44.4) | 43.7% (36.5 - 49.5) | 33.6% (28.9 - 36.9) | 32.4% (28.2 - 35.9) |
| 60% | 80% | 69.1% (65.0 - 72.3) | 77.4% (75.2 - 79.0) | 51.9% (42.4 - 58.7) | 57.4% (49.1 - 63.9) | 47.5% (41.4 - 51.5) | 46.4% (40.9 - 50.4) |
| 80% | 80% | 81.5% (77.3 - 84.6) | 87.4% (85.4 - 88.6) | 62.6% (52.4 - 69.1) | 67.4% (58.9 - 73.9) | 59.3% (52.5 - 63.7) | 58.6% (52.4 - 62.6) |
| 40% | 100% | 68.2% (65.8 - 70.1) | 76.3% (74.4 - 78.4) | 53.7% (46.0 - 59.5) | 56.8% (49.0 - 63.1) | 46.5% (41.6 - 49.6) | 45.2% (40.7 - 48.2) |
| 60% | 100% | 84.9% (82.9 - 86.3) | 89.4% (88.1 - 90.6) | 67.6% (59.5 - 73.6) | 70.1% (62.1 - 76.4) | 62.6% (57.1 - 66.0) | 61.7% (56.5 - 64.8) |
| 80% | 100% | 94.8% (93.5 - 95.5) | 96.4% (95.7 - 96.9) | 76.2% (68.5 - 81.9) | 78.3% (70.9 - 84.0) | 74.5% (69.1 - 77.8) | 74.5% (69.6 - 77.6) |

**Supplementary Table 9.** Model projections of the impact of a prophylactic HSV-2 vaccine after 40 years for model runs with HIV incidence rate ratios falling within the 95% confidence interval of estimates from (Looker, Johnston et al. 2020). Results show the impact of vaccinating a proportion (the uptake) of 9 year-olds each year from 2020 with a prophylactic vaccine which has lifelong protection and provides protection against HSV-2 acquisition. Table shows median values with 95% credibility intervals in parentheses.

| **Uptake** | **Efficacy** | **Coverage among 15-49 y.o. (2060)** | **Relative reduction in HSV-2 incidence (2060)** | **Relative reduction in HIV incidence (2060)** | **Relative reduction in number of GUD days (2060)** |
| --- | --- | --- | --- | --- | --- |
| 40% | 50% | 36.0% (35.3 - 37.2) | 32.3% (30.6 - 34.5) | 20.8% (17.3 - 24.9) | 16.2% (14.4 - 18.8) |
| 60% | 50% | 54.0% (53.0 - 55.8) | 45.0% (42.8 - 47.9) | 29.6% (25.0 - 35.0) | 24.0% (21.5 - 27.6) |
| 80% | 50% | 71.9% (70.6 - 74.3) | 55.8% (53.3 - 59.0) | 37.5% (32.0 - 43.8) | 31.5% (28.5 - 36.0) |
| 40% | 80% | 36.0% (35.4 - 37.3) | 54.9% (53.3 - 57.3) | 37.2% (32.2 - 42.8) | 30.4% (28.2 - 34.6) |
| 60% | 80% | 54.0% (53.0 - 55.8) | 71.2% (69.3 - 73.9) | 50.1% (44.2 - 56.8) | 43.7% (40.8 - 49.0) |
| 80% | 80% | 71.9% (70.6 - 74.3) | 82.8% (81.0 - 85.3) | 60.2% (53.7 - 67.2) | 55.6% (52.2 - 61.5) |
| 40% | 100% | 36.1% (35.4 - 37.3) | 70.9% (69.8 - 72.7) | 50.8% (45.2 - 57.1) | 42.8% (40.8 - 47.8) |
| 60% | 100% | 54.0% (53.0 - 55.9) | 86.1% (85.3 - 87.6) | 64.2% (58.5 - 70.7) | 59.1% (56.5 - 64.6) |
| 80% | 100% | 71.9% (70.6 - 74.4) | 95.0% (94.6 - 95.9) | 73.0% (67.4 - 78.9) | 72.0% (69.2 - 77.3) |

**Supplementary Table 10.** Model projections of the cumulative impact (proportion infections/GUD days averted) of a prophylactic HSV-2 vaccine over 2020-2060. Results show the impact of vaccinating a proportion (the uptake) of 9 year-olds each year from 2020 with a prophylactic vaccine which has lifelong protection and provides protection against HSV-2 acquisition. Table shows median values with 95% credibility intervals in parentheses.

| **Uptake** | **Efficacy** | **% HSV-2 infections averted**  **(2020-2060)** | **% HIV infections averted**  **(2020-2060)** | **% GUD Days averted**  **(2020-2060)** |
| --- | --- | --- | --- | --- |
| 40% | 50% | 14.7% (12.6 - 16.7) | 10.0% (7.7 - 11.9) | 8.6% (7.0 - 9.8) |
| 60% | 50% | 21.9% (18.8 - 24.6) | 14.5% (11.2 - 17.1) | 12.7% (10.3 - 14.4) |
| 80% | 50% | 28.9% (24.9 - 32.1) | 18.7% (14.5 - 21.9) | 16.6% (13.6 - 18.9) |
| 40% | 80% | 27.7% (24.4 - 30.0) | 18.1% (14.2 - 21.1) | 15.7% (13.0 - 17.6) |
| 60% | 80% | 39.9% (35.5 - 42.9) | 25.2% (20.1 - 29.1) | 22.6% (18.9 - 25.2) |
| 80% | 80% | 50.9% (45.8 - 54.1) | 31.2% (25.2 - 35.8) | 28.8% (24.3 - 32.1) |
| 40% | 100% | 38.7% (35.1 - 41.0) | 24.8% (20.1 - 29.0) | 21.6% (18.3 - 24.1) |
| 60% | 100% | 53.7% (49.4 - 56.5) | 33.0% (27.2 - 38.2) | 30.2% (25.9 - 33.5) |
| 80% | 100% | 65.7% (61.2 - 68.7) | 39.2% (32.7 - 45.0) | 37.4% (32.5 - 41.4) |

**Supplementary Table 11.** Model projections of the cumulative impact (infections/GUD days averted) of a prophylactic HSV-2 vaccine over 2020-2060. Results show the impact of vaccinating a proportion (the uptake) of 9 year-olds each year from 2020 with a prophylactic vaccine which has lifelong protection and provides protection against HSV-2 acquisition. Table shows median values with 95% credibility intervals in parentheses.

| **Uptake** | **Efficacy** | **HSV-2 infections averted**  **(2020-2060; millions)** | **HIV infections averted**  **(2020-2060; millions)** | **GUD Days averted**  **(2020-2060; millions)** |
| --- | --- | --- | --- | --- |
| 40% | 50% | 8.6 (5.8 - 11.3) | 2.1 (1.1 - 3.1) | 2.7 (1.7 - 4.3) |
| 60% | 50% | 12.7 (8.6 - 16.7) | 3.1 (1.6 - 4.5) | 4.0 (2.5 - 6.3) |
| 80% | 50% | 16.7 (11.4 - 22.0) | 3.9 (2.1 - 5.8) | 5.3 (3.3 - 8.2) |
| 40% | 80% | 16.0 (11.0 - 21.1) | 3.8 (2.1 - 5.6) | 5.0 (3.1 - 7.8) |
| 60% | 80% | 23.0 (16.0 - 30.3) | 5.3 (2.9 - 7.7) | 7.1 (4.5 - 11.1) |
| 80% | 80% | 29.3 (20.5 - 38.5) | 6.6 (3.6 - 9.5) | 9.1 (5.8 - 14.2) |
| 40% | 100% | 22.3 (15.6 - 29.5) | 5.3 (2.9 - 7.7) | 6.8 (4.3 - 10.7) |
| 60% | 100% | 30.9 (21.8 - 40.7) | 7.0 (3.9 - 10.2) | 9.5 (6.1 - 14.8) |
| 80% | 100% | 37.7 (27.1 - 49.6) | 8.3 (4.7 - 12.0) | 11.8 (7.7 - 18.3) |

**Supplementary Table 12.** Model projections of the impact of a therapeutic HSV-2 vaccine after 40 years. Results show the impact of vaccinating symptomatic individuals each year with a therapeutic vaccine which has lifelong protection and reduces days with asymptomatic or symptomatic shedding by the stated percentages given as the efficacy. Table shows median values with 95% credibility intervals in parentheses.

| **Coverage among symptomatic HSV-2 population (2060)** | **Efficacy** | **Coverage among 15-49 y.o. (2060)** | **Relative reduction in HSV-2 incidence (2060)** | **Relative reduction in HIV incidence (2060)** | **Relative reduction in number of GUD days (2060)** |
| --- | --- | --- | --- | --- | --- |
| 20% | 50% | 4.0% (2.9 - 5.3) | 9.7% (7.0 - 14.0) | 8.6% (5.8 - 13.4) | 18.3% (15.6 - 22.3) |
| 40% | 50% | 7.1% (5.2 - 9.4) | 18.8% (13.7 - 26.4) | 16.9% (11.7 - 25.3) | 33.3% (29.3 - 39.0) |
| 60% | 50% | 9.7% (7.1 - 12.7) | 27.3% (20.1 - 37.2) | 24.8% (17.6 - 35.4) | 45.3% (41.0 - 51.6) |
| 20% | 80% | 3.8% (2.8 - 5.1) | 15.4% (11.1 - 22.1) | 13.6% (9.2 - 20.9) | 28.6% (24.5 - 34.4) |
| 40% | 80% | 6.5% (4.8 - 8.6) | 29.6% (21.8 - 40.9) | 26.4% (18.5 - 38.0) | 50.8% (45.4 - 58.2) |
| 60% | 80% | 8.4% (6.3 - 11.1) | 42.6% (31.8 - 56.3) | 38.0% (27.7 - 51.9) | 67.6% (62.4 - 74.2) |
| 20% | 100% | 3.7% (2.7 - 5.0) | 19.1% (13.9 - 27.3) | 16.8% (11.5 - 25.6) | 35.2% (30.3 - 42.0) |
| 40% | 100% | 6.1% (4.5 - 8.1) | 36.6% (27.1 - 49.7) | 32.3% (22.9 - 45.6) | 61.5% (55.4 - 69.2) |
| 60% | 100% | 7.6% (5.6 - 10.1) | 52.2% (39.3 - 67.3) | 45.9% (34.0 - 60.9) | 80.0% (75.1 - 85.7) |

**Supplementary Table 13.** Model projections of the impact of a therapeutic HSV-2 vaccine after 40 years, by gender. Results show the impact of vaccinating symptomatic individuals each year with a therapeutic vaccine which has lifelong protection and reduces days with asymptomatic or symptomatic shedding by the stated percentages given as the efficacy. Table shows median values with 95% credibility intervals in parentheses.

| **Coverage among symptomatic HSV-2 population (2060)** | **Efficacy** | **Coverage among 15-49 y.o. (2060)** | | **Relative reduction in HSV-2 incidence (2060)** | | **Relative reduction in HIV incidence (2060)** | | **Relative reduction in number of GUD days (2060)** | |
| --- | --- | --- | --- | --- | --- | --- | --- | --- | --- |
|  |  | **Adult Males** | **Adult Females** | **Adult Males** | **Adult Females** | **Adult Males** | **Adult Females** | **Adult Males** | **Adult Females** |
| 20% | 50% | 2.8% (2.0 - 3.8) | 5.1% (3.7 - 6.9) | 10.1% (7.4 - 14.2) | 12.8% (9.5 - 17.6) | 8.7% (5.8 - 13.4) | 10.4% (7.2 - 15.8) | 20.3% (17.9 - 24.0) | 20.1% (17.6 - 23.9) |
| 40% | 50% | 5.0% (3.6 - 6.7) | 9.2% (6.7 - 12.3) | 18.4% (13.6 - 25.3) | 23.1% (17.4 - 31.1) | 16.1% (11.0 - 24.0) | 19.1% (13.6 - 27.9) | 35.0% (31.5 - 40.2) | 34.4% (30.9 - 39.8) |
| 60% | 50% | 6.9% (4.9 - 9.0) | 12.5% (9.2 - 16.6) | 25.7% (19.2 - 34.6) | 32.0% (24.3 - 42.1) | 22.7% (15.8 - 32.8) | 26.9% (19.5 - 37.9) | 46.3% (42.4 - 52.0) | 45.3% (41.4 - 51.3) |
| 20% | 80% | 2.7% (1.9 - 3.6) | 5.0% (3.6 - 6.6) | 16.1% (11.8 - 22.3) | 20.1% (15.0 - 27.3) | 13.7% (9.3 - 20.8) | 16.3% (11.4 - 24.3) | 31.6% (28.1 - 36.9) | 31.4% (27.7 - 36.8) |
| 40% | 80% | 4.6% (3.3 - 6.1) | 8.5% (6.3 - 11.3) | 29.0% (21.6 - 38.9) | 35.7% (27.1 - 46.8) | 25.0% (17.4 - 36.2) | 29.5% (21.4 - 41.7) | 52.9% (48.4 - 59.3) | 52.5% (47.8 - 59.1) |
| 60% | 80% | 5.9% (4.3 - 7.8) | 10.9% (8.2 - 14.5) | 40.0% (30.2 - 52.4) | 48.3% (37.4 - 61.4) | 34.7% (24.6 - 48.1) | 40.7% (30.3 - 54.9) | 68.0% (63.7 - 73.9) | 67.5% (62.9 - 73.8) |
| 20% | 100% | 2.6% (1.9 - 3.5) | 4.8% (3.5 - 6.5) | 20.0% (14.7 - 27.5) | 24.8% (18.5 - 33.5) | 17.0% (11.5 - 25.4) | 20.1% (14.2 - 29.6) | 38.7% (34.6 - 44.8) | 38.6% (34.2 - 44.8) |
| 40% | 100% | 4.3% (3.1 - 5.7) | 8.0% (5.9 - 10.6) | 35.8% (26.7 - 47.5) | 43.4% (33.2 - 56.1) | 30.5% (21.4 - 43.4) | 35.9% (26.3 - 49.6) | 63.5% (58.7 - 70.1) | 63.4% (58.2 - 70.2) |
| 60% | 100% | 5.3% (3.8 - 7.0) | 9.9% (7.4 - 13.2) | 48.9% (37.2 - 62.7) | 58.0% (45.4 - 71.8) | 41.9% (30.2 - 56.7) | 48.8% (36.9 - 63.8) | 80.0% (76.1 - 85.0) | 79.9% (75.6 - 85.3) |

**Supplementary Table 14.** Model projections of the impact of a therapeutic HSV-2 vaccine after 40 years for model runs with HIV incidence rate ratios falling within the 95% confidence interval of estimates from (Looker, Johnston et al. 2020). Results show the impact of vaccinating symptomatic individuals each year with a therapeutic vaccine which has lifelong protection and reduces days with asymptomatic or symptomatic shedding by the stated percentages given as the efficacy. Table shows median values with 95% credibility intervals in parentheses.

| **Coverage among symptomatic HSV-2 population (2060)** | **Efficacy** | **Relative reduction in HSV-2 incidence (2060)** | **Relative reduction in HIV incidence (2060)** | **Relative reduction in number of GUD days (2060)** |
| --- | --- | --- | --- | --- |
| 20% | 50% | 9.0% (6.9 - 12.1) | 7.5% (5.4 - 11.2) | 17.0% (15.2 - 20.4) |
| 40% | 50% | 17.6% (13.6 - 23.2) | 14.9% (11.0 - 21.4) | 31.5% (28.8 - 36.3) |
| 60% | 50% | 25.6% (20.0 - 33.2) | 22.0% (16.8 - 30.5) | 43.5% (40.6 - 48.6) |
| 20% | 80% | 14.3% (11.0 - 19.2) | 11.8% (8.6 - 17.5) | 26.7% (24.0 - 31.7) |
| 40% | 80% | 27.8% (21.7 - 36.3) | 23.2% (17.5 - 32.7) | 48.5% (44.7 - 54.9) |
| 60% | 80% | 40.3% (31.6 - 51.2) | 34.0% (26.4 - 45.7) | 65.5% (61.9 - 71.3) |
| 20% | 100% | 17.8% (13.8 - 23.8) | 14.7% (10.7 - 21.5) | 33.0% (29.7 - 38.9) |
| 40% | 100% | 34.4% (27.0 - 44.7) | 28.5% (21.7 - 39.6) | 58.8% (54.6 - 65.9) |
| 60% | 100% | 49.5% (39.2 - 62.0) | 41.3% (32.5 - 54.0) | 78.0% (74.5 - 83.4) |

**Supplementary Table 15.** Model projections of the cumulative impact (proportion infections/GUD days averted) of a therapeutic HSV-2 vaccine over 2020-2060. Results show the impact of vaccinating symptomatic individuals each year with a therapeutic vaccine which has lifelong protection and reduces days with asymptomatic or symptomatic shedding by the stated percentages given as the efficacy. Table shows median values with 95% credibility intervals in parentheses.

| **Coverage among symptomatic HSV-2 population (2060)** | **Efficacy** | **% HSV-2 infections averted**  **(2020-2060)** | **% HIV infections averted**  **(2020-2060)** | **% GUD Days averted**  **(2020-2060)** |
| --- | --- | --- | --- | --- |
| 20% | 50% | 4.3% (3.0 – 6.7) | 4.3% (2.8 – 6.7) | 13.9% (11.5 – 16.8) |
| 40% | 50% | 9.2% (6.3 – 13.9) | 8.8% (6.0 – 13.4) | 27.0% (23.3 – 31.4) |
| 60% | 50% | 14.4% (10.0 – 21.3) | 13.7% (9.5 – 19.9) | 39.0% (35.1 – 43.7) |
| 20% | 80% | 7.1% (4.8 – 10.9) | 6.8% (4.5 – 10.6) | 21.8% (18.2 – 26.2) |
| 40% | 80% | 15.1% (10.4 – 22.9) | 13.9% (9.5 – 20.8) | 41.8% (36.4 – 47.9) |
| 60% | 80% | 24.2% (16.7 - 35.3) | 21.4% (15.1 - 30.4) | 59.3% (54.0 - 65.0) |
| 20% | 100% | 8.9% (6.1 - 13.8) | 8.4% (5.6 - 13.1) | 27.0% (22.5 - 32.2) |
| 40% | 100% | 19.3% (13.2 - 29.0) | 17.2% (11.8 - 25.3) | 51.0% (44.7 - 57.8) |
| 60% | 100% | 31.1% (21.5 - 44.6) | 26.2% (18.8 - 36.6) | 71.4% (65.6 - 76.9) |

**Supplementary Table 16.** Model projections of the cumulative impact (infections/GUD days averted) of a therapeutic HSV-2 vaccine over 2020-2060. Results show the impact of vaccinating symptomatic individuals each year with a therapeutic vaccine which has lifelong protection and reduces days with asymptomatic or symptomatic shedding by the stated percentages given as the efficacy. Table shows median values with 95% credibility intervals in parentheses.

| **Coverage among symptomatic HSV-2 population (2060)** | **Efficacy** | **HSV-2 infections averted**  **(2020-2060; millions)** | **HIV infections averted**  **(2020-2060; millions)** | **GUD Days averted**  **(2020-2060; millions)** |
| --- | --- | --- | --- | --- |
| 20% | 50% | 2.5 (1.5 - 4.2) | 0.9 (0.4 - 1.6) | 4.4 (2.6 - 7.6) |
| 40% | 50% | 5.3 (3.3 - 8.7) | 1.9 (1.0 - 3.3) | 8.5 (5.3 - 14.3) |
| 60% | 50% | 8.3 (5.2 - 13.3) | 2.9 (1.5 - 4.9) | 12.3 (7.9 - 20.2) |
| 20% | 80% | 4.1 (2.5 - 6.9) | 1.4 (0.7 - 2.6) | 6.9 (4.1 - 11.8) |
| 40% | 80% | 8.7 (5.4 - 14.4) | 2.9 (1.5 - 5.1) | 13.2 (8.2 - 21.9) |
| 60% | 80% | 13.8 (8.7 - 22.3) | 4.5 (2.4 - 7.5) | 18.7 (12.2 - 30.1) |
| 20% | 100% | 5.1 (3.1 - 8.7) | 1.8 (0.9 - 3.2) | 8.5 (5.1 - 14.6) |
| 40% | 100% | 11.1 (6.8 - 18.3) | 3.6 (1.9 - 6.3) | 16.1 (10.1 - 26.5) |
| 60% | 100% | 17.7 (11.2 - 28.5) | 5.5 (2.9 - 9.1) | 22.5 (14.7 - 35.7) |

# References

Abuelezam, N. N., A. W. McCormick, T. Fussell, A. N. Afriyie, R. Wood, V. DeGruttola, K. A. Freedberg, M. Lipsitch and G. R. Seage, 3rd (2016). "Can the Heterosexual HIV Epidemic be Eliminated in South Africa Using Combination Prevention? A Modeling Analysis." Am J Epidemiol **184**(3): 239-248.

Andersson, K. M., R. M. Van Niekerk, L. M. Niccolai, O. N. Mlungwana, I. M. Holdsworth, M. Bogoshi, J. A. McIntyre, G. E. Gray and E. Vardas (2009). "Sexual risk behaviour of the first cohort undergoing screening for enrolment into Phase I/II HIV vaccine trials in South Africa." International Journal of STD & AIDS **20**(2): 95-101.

Aoki, F. Y., S. Tyring, F. Diaz-Mitoma, G. Gross, J. Gao and K. Hamed (2006). "Single-day, patient-initiated famciclovir therapy for recurrent genital herpes: a randomized, double-blind, placebo-controlled trial." Clin Infect Dis **42**(1): 8-13.

Arnold, M. P., H. Struthers, J. McIntyre and T. Lane (2013). "Contextual correlates of per partner unprotected anal intercourse rates among MSM in Soweto, South Africa." AIDS Behav **17 Suppl 1**: S4-11.

Baggaley, R. F., R. G. White and M. C. Boily (2010). "HIV transmission risk through anal intercourse: systematic review, meta-analysis and implications for HIV prevention." Int J Epidemiol **39**(4): 1048-1063.

Baral, S., E. Burrell, A. Scheibe, B. Brown, C. Beyrer and L. G. Bekker (2011). "HIV risk and associations of HIV infection among men who have sex with men in peri-urban Cape Town, South Africa." BMC Public Health **11**: 766.

Barnabas, R. V., H. van Rooyen, E. Tumwesigye, P. M. Murnane, J. M. Baeten, H. Humphries, B. Turyamureeba, P. Joseph, M. Krows, J. P. Hughes and C. Celum (2014). "Initiation of antiretroviral therapy and viral suppression after home HIV testing and counselling in KwaZulu-Natal, South Africa, and Mbarara district, Uganda: a prospective, observational intervention study." Lancet HIV **1**(2): e68-e76.

Barth, R. E., H. A. Tempelman, R. Moraba and A. I. Hoepelman (2011). "Long-Term Outcome of an HIV-Treatment Programme in Rural Africa: Viral Suppression despite Early Mortality." AIDS Res Treat **2011**: 434375.

Behanzin, L., S. Diabate, I. Minani, C. M. Lowndes, M. C. Boily, A. C. Labbe, S. Anagonou, D. M. Zannou, A. Buve and M. Alary (2013). "Assessment of HIV-related risky behaviour: a comparative study of face-to-face interviews and polling booth surveys in the general population of Cotonou, Benin." Sex Transm Infect **89**(7): 595-601.

Bekker, L. G., L. Johnson, F. Cowan, C. Overs, D. Besada, S. Hillier and W. Cates, Jr. (2015). "Combination HIV prevention for female sex workers: what is the evidence?" Lancet **385**(9962): 72-87.

Bender Ignacio, R. A., T. Perti, A. S. Magaret, S. Rajagopal, C. E. Stevens, M. L. Huang, S. Selke, C. Johnston, J. Marrazzo and A. Wald (2015). "Oral and Vaginal Tenofovir for Genital Herpes Simplex Virus Type 2 Shedding in Immunocompetent Women: A Double-Blind, Randomized, Cross-over Trial." J Infect Dis **212**(12): 1949-1956.

Benedetti, J. K., J. Zeh and L. Corey (1999). "Clinical reactivation of genital herpes simplex virus infection decreases in frequency over time." Ann Intern Med **131**(1): 14-20.

Benedetti, J. K., J. Zeh, S. Selke and L. Corey (1995). "Frequency and reactivation of nongenital lesions among patients with genital herpes simplex virus." Am J Med **98**(3): 237-242.

Bernstein, D. I., A. Wald, T. Warren, K. Fife, S. Tyring, P. Lee, N. Van Wagoner, A. Magaret, J. B. Flechtner, S. Tasker, J. Chan, A. Morris and S. Hetherington (2017). "Therapeutic Vaccine for Genital Herpes Simplex Virus-2 Infection: Findings From a Randomized Trial." J Infect Dis **215**(6): 856-864.

Boggess, K. A., D. H. Watts, A. C. Hobson, R. L. Ashley, Z. A. Brown and L. Corey (1997). "Herpes simplex virus type 2 detection by culture and polymerase chain reaction and relationship to genital symptoms and cervical antibody status during the third trimester of pregnancy." Am J Obstet Gynecol **176**(2): 443-451.

Boily, M. C., R. F. Baggaley, L. Wang, B. Masse, R. G. White, R. J. Hayes and M. Alary (2009). "Heterosexual risk of HIV-1 infection per sexual act: systematic review and meta-analysis of observational studies." Lancet Infect Dis **9**(2): 118-129.

Brown, Z. A., J. K. Benedetti, D. H. Watts, S. Selke, S. Berry, R. L. Ashley and L. Corey (1995). "A comparison of between detailed and simple histories in the diagnosis of genital herpes complicating pregnancy." American Journal of Obstetrics and Gynecology **172**(4): 1299-1303.

Bryson, Y., M. Dillon, D. I. Bernstein, J. Radolf, P. Zakowski and E. Garratty (1993). "Risk of acquisition of genital herpes simplex virus type 2 in sex partners of persons with genital herpes: a prospective couple study." J Infect Dis **167**(4): 942-946.

Bryson, Y., M. Dillon, M. Lovett, D. Bernstein, E. Garratty and J. Sayre (1985). "Treatment of first episode genital HSV with oral acyclovir: long term follow-up of recurrences. A preliminary report." Scand J Infect Dis Suppl **47**: 70-75.

Carael, M., E. Slaymaker, R. Lyerla and S. Sarkar (2006). "Clients of sex workers in different regions of the world: hard to count." Sex Transm Infect **82 Suppl 3**: iii26-33.

Cloete, A., S. Jooste, M. Mabaso, L. Simbayi, T. Rehle and P. Naidoo (2014). "The South African Marang men's project: HIV bio-behavioural surveys conducted among men who have sex with men in Cape Town, Durban and Johannesburg using respondent driven sampling."

Coetzee, J., G. Hunt, M. Jaffer, K. Otwombe, L. Scott, A. Bongwe, J. Ledwaba, S. Molema, R. Jewkes and G. E. Gray (2017). "HIV-1 viraemia and drug resistance amongst female sex workers in Soweto, South Africa: A cross sectional study." PLoS One **12**(12): e0188606.

Coetzee, J., R. Jewkes and G. E. Gray (2017). "Cross-sectional study of female sex workers in Soweto, South Africa: Factors associated with HIV infection." PLoS One **12**(10): e0184775.

Cohen, F., M. E. Kemeny, K. A. Kearney, L. S. Zegans, J. M. Neuhaus and M. A. Conant (1999). "Persistent stress as a predictor of genital herpes recurrence." Arch Intern Med **159**(20): 2430-2436.

Corey, L., A. J. Nahmias, M. E. Guinan, J. K. Benedetti, C. W. Critchlow and K. K. Holmes (1982). "A trial of topical acyclovir in genital herpes simplex virus infections." N Engl J Med **306**(22): 1313-1319.

de Bruyn, G., M. Vargas-Cortez, T. Warren, S. K. Tyring, K. H. Fife, J. Lalezari, R. C. Brady, M. Shahmanesh, G. Kinghorn, K. R. Beutner, R. Patel, M. A. Drehobl, P. Horner, T. O. Kurtz, S. McDermott, A. Wald and L. Corey (2006). "A randomized controlled trial of a replication defective (gH deletion) herpes simplex virus vaccine for the treatment of recurrent genital herpes among immunocompetent subjects." Vaccine **24**(7): 914-920.

Douglas, J. M., C. Critchlow, J. Benedetti, G. J. Mertz, J. D. Connor, M. A. Hintz, A. Fahnlander, M. Remington, C. Winter and L. Corey (1984). "A double-blind study of oral acyclovir for suppression of recurrences of genital herpes simplex virus infection." N Engl J Med **310**(24): 1551-1556.

Dunkle, K. L., M. E. Beksinska, V. H. Rees, R. C. Ballard, Y. Htun and M. L. Wilson (2005). "Risk factors for HIV infection among sex workers in Johannesburg, South Africa." Int J STD AIDS **16**(3): 256-261.

Dunkle, K. L., R. K. Jewkes, D. W. Murdock, Y. Sikweyiya and R. Morrell (2013). "Prevalence of consensual male-male sex and sexual violence, and associations with HIV in South Africa: a population-based cross-sectional study." PLoS Med **10**(6): e1001472.

Eakle, R., G. B. Gomez, N. Naicker, R. Bothma, J. Mbogua, M. A. Cabrera Escobar, E. Saayman, M. Moorhouse, W. D. F. Venter, H. Rees and T. D. P. Team (2017). "HIV pre-exposure prophylaxis and early antiretroviral treatment among female sex workers in South Africa: Results from a prospective observational demonstration project." PLoS Med **14**(11): e1002444.

Fanfair, R. N., A. Zaidi, L. D. Taylor, F. Xu, S. Gottlieb and L. Markowitz (2013). "Trends in seroprevalence of herpes simplex virus type 2 among non-Hispanic blacks and non-Hispanic whites aged 14 to 49 years--United States, 1988 to 2010." Sex Transm Dis **40**(11): 860-864.

Fatti, G., E. Mothibi, G. Meintjes and A. Grimwood (2014). "Antiretroviral treatment outcomes amongst older adults in a large multicentre cohort in South Africa." PLoS One **9**(6): e100273.

Fife, K. H., T. J. Warren, S. E. Justus, C. K. Heitman and T. Hs2100275 Study (2008). "An international, randomized, double-blind, placebo-controlled, study of valacyclovir for the suppression of herpes simplex virus type 2 genital herpes in newly diagnosed patients." Sex Transm Dis **35**(7): 668-673.

Fife, K. H., J. A. Williams, A. L. Thomas, S. Ofner, B. P. Katz and J. D. Fortenberry (2010). "Herpes simplex virus type 2 infection in young adult women: risk factors for infection and frequency of viral shedding." Sex Transm Dis **37**(4): 248-252.

Foss, A. M., P. T. Vickerman, Z. Chalabi, P. Mayaud, M. Alary and C. H. Watts (2009). "Dynamic modeling of herpes simplex virus type-2 (HSV-2) transmission: issues in structural uncertainty." Bull Math Biol **71**(3): 720-749.

Franzen-Rohl, E., D. Schepis, F. Atterfelt, K. Franck, A. Wikstrom, J. A. Liljeqvist, T. Bergstrom, E. Aurelius, K. Karre, L. Berg and H. Gaines (2017). "Herpes simplex virus specific T cell response in a cohort with primary genital infection correlates inversely with frequency of subsequent recurrences." Sex Transm Infect **93**(3): 169-174.

Gomez, G. B., R. Eakle, J. Mbogua, G. Akpomiemie, W. D. Venter and H. Rees (2016). "Treatment And Prevention for female Sex workers in South Africa: protocol for the TAPS Demonstration Project." BMJ Open **6**(9): e011595.

Gupta, R., A. Wald, E. Krantz, S. Selke, T. Warren, M. Vargas-Cortes, G. Miller and L. Corey (2004). "Valacyclovir and acyclovir for suppression of shedding of herpes simplex virus in the genital tract." J Infect Dis **190**(8): 1374-1381.

Handsfield, H. H., T. Warren, M. Werner and J. A. Phillips (2007). "Suppressive therapy with valacyclovir in early genital herpes: a pilot study of clinical efficacy and herpes-related quality of life." Sex Transm Dis **34**(6): 339-343.

Hollingsworth, T. D., R. M. Anderson and C. Fraser (2008). "HIV-1 transmission, by stage of infection." J Infect Dis **198**(5): 687-693.

Huerga, H., F. Shiferie, E. Grebe, R. Giuliani, J. B. Farhat, G. Van-Cutsem and K. Cohen (2017). "A comparison of self-report and antiretroviral detection to inform estimates of antiretroviral therapy coverage, viral load suppression and HIV incidence in Kwazulu-Natal, South Africa." BMC Infect Dis **17**(1): 653.

Hughes, J. P., J. M. Baeten, J. R. Lingappa, A. S. Magaret, A. Wald, G. de Bruyn, J. Kiarie, M. Inambao, W. Kilembe, C. Farquhar, C. Celum and H. S. V. H. I. V. T. S. T. Partners in Prevention (2012). "Determinants of per-coital-act HIV-1 infectivity among African HIV-1-serodiscordant couples." J Infect Dis **205**(3): 358-365.

Hugo, J. M., R. D. Stall, K. Rebe, J. E. Egan, G. Jobson, G. De Swardt, H. Struthers and J. A. McIntyre (2016). "Knowledge, Attitudes and Beliefs regarding Post Exposure Prophylaxis among South African Men who have Sex with Men." AIDS Behav **20**(Suppl 3): 350-356.

Human Sciences Research Council (2002). South African HIV/AIDS, Behavioural Risks, Sero-status, and Mass Media Impact Survey (SABSSM) 2002: Adult and youth data - All provinces. [Data set]. SABSSM 2002 Adult-youth. Version 1.0. Pretoria South Africa: Human Sciences Research Council [producer] 2002, Human Sciences Research Council [distributor] 2011. <http://dx.doi.org/doi:10.14749/1400830395>.

Human Sciences Research Council (2005). South African National HIV Prevalence, HIV Incidence, Behaviour and Communication Survey (SABSSM) 2005: Adult and youth data - All provinces. [Data set]. SABSSM 2005 Adult-youth. Version 1.0. Pretoria South Africa: Human Sciences Research Council [producer] 2005, Human Sciences Research Council [distributor] 2011. <http://dx.doi.org/doi:10.14749/1400830455>.

Human Sciences Research Council (2008). South African National HIV Prevalence, HIV Incidence, Behaviour and Communication Survey (SABSSM) 2008: Adult - All provinces. [Data set]. SABSSM 2008 Adult. Version 1.0. Pretoria South Africa: Human Sciences Research Council [producer] 2009, Human Sciences Research Council [distributor] 2014. <http://dx.doi.org/doi:10.14749/1434098373>.

Human Sciences Research Council (2012). South African National HIV Prevalence, HIV Incidence, Behaviour and Communication Survey (SABSSM) 2012: Adult - All provinces. [Data set]. SABSSM 2012 Adult. Version 1.0. Pretoria South Africa: Human Sciences Research Council [producer] 2012, Human Sciences Research Council [distributor] 2016. <http://dx.doi.org/doi:10.14749/1500530684>.

Human Sciences Research Council (2017). "The Fifth South African National HIV Prevalence, Incidence, Behaviour and Communication Survey, 2017."

Jean, K., A. Puren, E. Cutler, B. Singh, J. Bouscaillou, R. Rain-Taljaard, D. Taljaard, E. Gouws, P. Lissouba, D. A. Lewis, G. Peytavin and B. Auvert (2016). "Level of viral suppression and the cascade of HIV care in a South African semi-urban setting in 2012." AIDS **30**(13): 2107-2116.

Jewkes, R., K. Dunkle, M. Nduna, J. Levin, N. Jama, N. Khuzwayo, M. Koss, A. Puren and N. Duvvury (2006). "Factors associated with HIV sero-positivity in young, rural South African men." Int J Epidemiol **35**(6): 1455-1460.

Johansson, K. A., B. Robberstad and O. F. Norheim (2010). "Further benefits by early start of HIV treatment in low income countries: survival estimates of early versus deferred antiretroviral therapy." AIDS Res Ther **7**(1): 3.

Johnson, L. F., C. Chiu, L. Myer, M. A. Davies, R. E. Dorrington, L. G. Bekker, A. Boulle and G. Meyer-Rath (2016). "Prospects for HIV control in South Africa: a model-based analysis." Glob Health Action **9**: 30314.

Johnson, L. F., P. Mulongeni, A. Marr and T. Lane (2018). "Age bias in survey sampling and implications for estimating HIV prevalence in men who have sex with men: insights from mathematical modelling." Epidemiol Infect **146**(8): 1036-1042.

Kalichman, S. C., L. C. Simbayi, D. Cain and S. Jooste (2009). "Heterosexual anal intercourse among community and clinical settings in Cape Town, South Africa." Sex Transm Infect **85**(6): 411-415.

Kapiamba, G., T. Masango and D. Mphuthi (2016). "Antiretroviral adherence and virological outcomes in HIV-positive patients in Ugu district, KwaZulu-Natal province." Afr J AIDS Res **15**(3): 195-201.

Kelly, H. and P. Mayaud (2019). The effect of antiretroviral therapy on herpes simplex virus type-2 (HSV-2) genital shedding, clinical manifestation, transmission and acquisition: a systematic review and meta-analysis.

Kincaid, D. L., S. Babalola and M. E. Figueroa (2014). "HIV communication programs, condom use at sexual debut, and HIV infections averted in South Africa, 2005." J Acquir Immune Defic Syndr **66 Suppl 3**: S278-284.

Knox, J., V. Reddy, F. Kaighobadi, D. Nel and T. Sandfort (2013). "Communicating HIV status in sexual interactions: assessing social cognitive constructs, situational factors, and individual characteristics among South African MSM." AIDS Behav **17**(1): 350-359.

Knox, J., V. Reddy, T. Lane, D. Hasin and T. Sandfort (2017). "Substance Use and Sexual Risk Behavior Among Black South African Men Who Have Sex With Men: The Moderating Effects of Reasons for Drinking and Safer Sex Intentions." AIDS Behav **21**(7): 2023-2032.

Knox, J., H. Yi, V. Reddy, S. Maimane and T. Sandfort (2010). "The fallacy of intimacy: sexual risk behaviour and beliefs about trust and condom use among men who have sex with men in South Africa." Psychol Health Med **15**(6): 660-671.

Koelle, D. M., J. Benedetti, A. Langenberg and L. Corey (1992). "Asymptomatic reactivation of herpes simplex virus in women after the first episode of genital herpes." Ann Intern Med **116**(6): 433-437.

Kufa, T., T. Lane, A. Manyuchi, B. Singh, Z. Isdahl, T. Osmand, M. Grasso, H. Struthers, J. McIntyre, Z. Chipeta and A. Puren (2017). "The accuracy of HIV rapid testing in integrated bio-behavioral surveys of men who have sex with men across 5 Provinces in South Africa." Medicine (Baltimore) **96**(28): e7391.

Lane, T., T. Osmand, A. Marr, S. B. Shade, K. Dunkle, T. Sandfort, H. Struthers, S. Kegeles and J. A. McIntyre (2014). "The Mpumalanga Men's Study (MPMS): results of a baseline biological and behavioral HIV surveillance survey in two MSM communities in South Africa." PLoS One **9**(11): e111063.

Lane, T., H. F. Raymond, S. Dladla, J. Rasethe, H. Struthers, W. McFarland and J. McIntyre (2011). "High HIV prevalence among men who have sex with men in Soweto, South Africa: results from the Soweto Men's Study." AIDS Behav **15**(3): 626-634.

Lane, T., S. B. Shade, J. McIntyre and S. F. Morin (2008). "Alcohol and sexual risk behavior among men who have sex with men in South african township communities." AIDS Behav **12**(4 Suppl): S78-85.

Langenberg, A., J. Benedetti, J. Jenkins, R. Ashley, C. Winter and L. Corey (1989). "Development of clinically recognizable genital lesions among women previously identified as having "asymptomatic" herpes simplex virus type 2 infection." Ann Intern Med **110**(11): 882-887.

Langenberg, A. G., L. Corey, R. L. Ashley, W. P. Leong and S. E. Straus (1999). "A prospective study of new infections with herpes simplex virus type 1 and type 2. Chiron HSV Vaccine Study Group." N Engl J Med **341**(19): 1432-1438.

Lecher, S. (2016). "Progress with scale-up of HIV viral load monitoring—seven sub-Saharan African countries, January 2015–June 2016." MMWR. Morbidity and mortality weekly report **65**.

Leone, P., M. Abudalu, E. Mitha, M. Gani, W. Zhou and K. Hamed (2010). "One-day famciclovir vs. placebo in patient-initiated episodic treatment of recurrent genital herpes in immunocompetent Black patients." Curr Med Res Opin **26**(3): 653-661.

Leone, P., T. Warren, K. Hamed, K. Fife and A. Wald (2007). "Famciclovir reduces viral mucosal shedding in HSV-seropositive persons." Sex Transm Dis **34**(11): 900-907.

Lippman, S. A., T. Lane, O. Rabede, H. Gilmore, Y. H. Chen, N. Mlotshwa, K. Maleke, A. Marr and J. A. McIntyre (2018). "High Acceptability and Increased HIV-Testing Frequency After Introduction of HIV Self-Testing and Network Distribution Among South African MSM." J Acquir Immune Defic Syndr **77**(3): 279-287.

Lippman, S. A., S. B. Shade, A. M. El Ayadi, J. M. Gilvydis, J. S. Grignon, T. Liegler, J. Morris, E. Naidoo, L. M. Prach, A. Puren and S. Barnhart (2016). "Attrition and Opportunities Along the HIV Care Continuum: Findings From a Population-Based Sample, North West Province, South Africa." J Acquir Immune Defic Syndr **73**(1): 91-99.

Looker, K. J., J. A. R. Elmes, S. L. Gottlieb, J. T. Schiffer, P. Vickerman, K. M. E. Turner and M. C. Boily (2017). "Effect of HSV-2 infection on subsequent HIV acquisition: an updated systematic review and meta-analysis." Lancet Infect Dis **17**(12): 1303-1316.

Looker, K. J., C. Johnston, N. J. Welton, C. James, P. Vickerman, K. M. E. Turner, M. C. Boily and S. L. Gottlieb (2020). "The global and regional burden of genital ulcer disease due to herpes simplex virus: a natural history modelling study." BMJ Glob Health **5**(3): e001875.

Mark, K. E., L. Corey, T. C. Meng, A. S. Magaret, M. L. Huang, S. Selke, H. B. Slade, S. K. Tyring, T. Warren, S. L. Sacks, P. Leone, V. A. Bergland and A. Wald (2007). "Topical resiquimod 0.01% gel decreases herpes simplex virus type 2 genital shedding: a randomized, controlled trial." J Infect Dis **195**(9): 1324-1331.

Mayatula, V. and T. R. Mavundla (1997). "A review on male circumcision procedures among South African blacks." Curationis **20**(3): 16-20.

Mberi, M. N., L. R. Kuonza, N. M. Dube, C. Nattey, S. Manda and R. Summers (2015). "Determinants of loss to follow-up in patients on antiretroviral treatment, South Africa, 2004-2012: a cohort study." BMC Health Serv Res **15**: 259.

McNaghten, A., R. Kearns, A. J. Siegler, N. Phaswana-Mafuya, L. G. Bekker, R. Stephenson, S. D. Baral, R. Brookmeyer, C. S. Yah, A. J. Lambert, B. Brown, E. Rosenberg, M. Blalock Tharp, A. de Voux, C. Beyrer and P. S. Sullivan (2014). "Sibanye Methods for Prevention Packages Program Project Protocol: Pilot Study of HIV Prevention Interventions for Men Who Have Sex With Men in South Africa." JMIR Res Protoc **3**(4): e55.

Meissner, O. and D. L. Buso (2007). "Traditional male circumcision in the Eastern Cape--scourge or blessing?" S Afr Med J **97**(5): 371-373.

Mills, E. J., C. Bakanda, J. Birungi, K. Chan, N. Ford, C. L. Cooper, J. B. Nachega, M. Dybul and R. S. Hogg (2011). "Life expectancy of persons receiving combination antiretroviral therapy in low-income countries: a cohort analysis from Uganda." Ann Intern Med **155**(4): 209-216.

Moyo, F., C. Chasela, A. T. Brennan, O. Ebrahim, I. M. Sanne, L. Long and D. Evans (2016). "Treatment outcomes of HIV-positive patients on first-line antiretroviral therapy in private versus public HIV clinics in Johannesburg, South Africa." Clin Epidemiol **8**: 37-47.

Mukandavire, C., J. Walker, S. Schwartz, M. C. Boily, L. Danon, C. Lyons, D. Diouf, B. Liestman, N. L. Diouf, F. Drame, K. Coly, R. S. M. Muhire, S. Thiam, P. A. N. Diallo, C. T. Kane, C. Ndour, E. Volz, S. Mishra, S. Baral and P. Vickerman (2018). "Estimating the contribution of key populations towards the spread of HIV in Dakar, Senegal." J Int AIDS Soc **21 Suppl 5**: e25126.

Muller, E. E., K. Rebe, T. F. Chirwa, H. Struthers, J. McIntyre and D. A. Lewis (2016). "The prevalence of human papillomavirus infections and associated risk factors in men-who-have-sex-with-men in Cape Town, South Africa." BMC Infect Dis **16**(1): 440.

Owen, B. N., J. Elmes, R. Silhol, Q. Dang, I. McGowan, B. Shacklett, E. M. Swann, A. Van der Straten, R. F. Baggaley and M. C. Boily (2017). "How common and frequent is heterosexual anal intercourse among South Africans? A systematic review and meta‐analysis." Journal of the International AIDS Society **20**(1): 21162.

Parry, C., P. Petersen, S. Dewing, T. Carney, R. Needle, K. Kroeger and L. Treger (2008). "Rapid assessment of drug-related HIV risk among men who have sex with men in three South African cities." Drug Alcohol Depend **95**(1-2): 45-53.

Peacock, J. E., Jr., L. G. Kaplowitz, P. F. Sparling, D. T. Durack, J. W. Gnann, Jr., R. J. Whitley, M. Lovett, Y. J. Bryson, R. J. Klein, A. E. Friedman-Kien and et al. (1988). "Intravenous acyclovir therapy of first episodes of genital herpes: a multicenter double-blind, placebo-controlled trial." Am J Med **85**(3): 301-306.

PEFSW (2015). Female Sex Workers in Port Elizabeth.

Peltzer, K., T. A. Mashego and M. Mabeba (2003). "Attitudes and practices of doctors toward domestic violence victims in South Africa." Health Care Women Int **24**(2): 149-157.

Peltzer, K., P. Seoka and S. Raphala (2004). "Characteristics of female sex workers and their HIV/AIDS/STI knowledge, attitudes and behaviour in semi-urban areas in South Africa." Curationis **27**(1): 4-11.

Phipps, W., E. Nakku-Joloba, E. M. Krantz, S. Selke, M. L. Huang, F. Kambugu, J. Orem, C. Casper, L. Corey and A. Wald (2016). "Genital Herpes Simplex Virus Type 2 Shedding Among Adults With and Without HIV Infection in Uganda." J Infect Dis **213**(3): 439-447.

Phipps, W., M. Saracino, A. Magaret, S. Selke, M. Remington, M. L. Huang, T. Warren, C. Casper, L. Corey and A. Wald (2011). "Persistent genital herpes simplex virus-2 shedding years following the first clinical episode." J Infect Dis **203**(2): 180-187.

Phiri, S., I. F. Hoffman, H. A. Weiss, F. Martinson, N. Nyirenda, D. Kamwendo, S. A. Fiscus, C. Y. Chen, W. C. Miller, L. van der Hoeven, D. Chilongozi, M. S. Cohen and P. Mayaud (2010). "Impact of aciclovir on ulcer healing, lesional, genital and plasma HIV-1 RNA among patients with genital ulcer disease in Malawi." Sex Transm Infect **86**(5): 345-352.

Quaife, M., R. Eakle, M. Cabrera, P. Vickerman, M. Tsepe, F. Cianci, S. Delany-Moretlwe and F. Terris-Prestholt (2016). "Preferences for ARV-based HIV prevention methods among men and women, adolescent girls and female sex workers in Gauteng Province, South Africa: a protocol for a discrete choice experiment." BMJ Open **6**(6): e010682.

Ramjee, G., S. S. Karim and A. W. Sturm (1998). "Sexually transmitted infections among sex workers in KwaZulu-Natal, South Africa." Sex Transm Dis **25**(7): 346-349.

Rao, A., S. Baral, N. Phaswana-Mafuya, A. Lambert, Z. Kose, M. McIngana, C. Holland, S. Ketende and S. Schwartz (2016). "Pregnancy Intentions and Safer Pregnancy Knowledge Among Female Sex Workers in Port Elizabeth, South Africa." Obstet Gynecol **128**(1): 15-21.

Rebe, K., D. Lewis, L. Myer, G. de Swardt, H. Struthers, M. Kamkuemah and J. McIntyre (2015). "A Cross Sectional Analysis of Gonococcal and Chlamydial Infections among Men-Who-Have-Sex-with-Men in Cape Town, South Africa." PLoS One **10**(9): e0138315.

Rees, H., M. E. Beksinska, K. Dickson-Tetteh, R. Ballard and Y. Htun (2000). "Commercial sex workers in Johannesburg: risk behaviour and HIV status." South African Journal of Science **96**(6).

Rees, K., O. Radebe, C. Arendse, C. Modibedi, H. E. Struthers, J. A. McIntyre and R. P. H. Peters (2017). "Utilization of Sexually Transmitted Infection Services at 2 Health Facilities Targeting Men Who Have Sex With Men in South Africa: A Retrospective Analysis of Operational Data." Sex Transm Dis **44**(12): 768-773.

RHRU (2000). "Reproductive Health Research Unit Behavioural Sentinel Survey data report - Antenatal Clinic - Free State; 2000."

RHRU (2000). "Reproductive Health Research Unit Behavioural Sentinel Survey data report - Clients of family planning - Gauteng.".

RHRU (2000). "Reproductive Health Research Unit Behavioural Sentinel Survey data report - Male STD clinic attenders - Gauteng.".

Rispel, L. C., C. A. Metcalf, A. Cloete, V. Reddy and C. Lombard (2011). "HIV prevalence and risk practices among men who have sex with men in two South African cities." J Acquir Immune Defic Syndr **57**(1): 69-76.

Rosen, S., M. Maskew, M. P. Fox, C. Nyoni, C. Mongwenyana, G. Malete, I. Sanne, D. Bokaba, C. Sauls, J. Rohr and L. Long (2016). "Initiating Antiretroviral Therapy for HIV at a Patient's First Clinic Visit: The RapIT Randomized Controlled Trial." PLoS Med **13**(5): e1002015.

SAHMS-FSW (2014). South African Health Monitoring Survey (SAHMS): An Integrated Biological and Behavioural Survey among Female Sex Workers, South Africa 2013-2014.

Sandfort, T. G., T. Lane, C. Dolezal and V. Reddy (2015). "Gender Expression and Risk of HIV Infection Among Black South African Men Who Have Sex with Men." AIDS Behav **19**(12): 2270-2279.

Sandfort, T. G., J. Nel, E. Rich, V. Reddy and H. Yi (2008). "HIV testing and self-reported HIV status in South African men who have sex with men: results from a community-based survey." Sex Transm Infect **84**(6): 425-429.

Schoub, B. D., A. N. Smith, S. Johnson, D. J. Martin, S. F. Lyons, G. N. Padayachee and H. S. Hurwitz (1990). "Considerations on the further expansion of the AIDS epidemic in South Africa--1990." S Afr Med J **77**(12): 613-618.

Shearer, K., A. T. Brennan, M. Maskew, L. Long, R. Berhanu, I. Sanne and M. P. Fox (2014). "The relation between efavirenz versus nevirapine and virologic failure in Johannesburg, South Africa." J Int AIDS Soc **17**(1): 19065.

Siegfried, N., M. Muller, J. J. Deeks and J. Volmink (2009). "Male circumcision for prevention of heterosexual acquisition of HIV in men." Cochrane Database Syst Rev(2): CD003362.

Siegler, A. J., A. Voux, N. Phaswana-Mafuya, L. G. Bekker, P. S. Sullivan, S. D. Baral, K. Winskell, Z. Kose, A. L. Wirtz and R. Stephenson (2014). "Elements of Condom-Use Decision Making among South African Men Who Have Sex with Men." J Int Assoc Provid AIDS Care **13**(5): 414-423.

Silhol, R., H. Coupland, R. F. Baggaley, L. Miller, L. Staadegaard, S. L. Gottlieb, J. Stannah, K. M. Turner, P. Vickerman and R. Hayes (2021). "What Is the Burden of Heterosexually Acquired HIV Due to HSV-2? Global and Regional Model-Based Estimates of the Proportion and Number of HIV Infections Attributable to HSV-2 Infection." JAIDS Journal of Acquired Immune Deficiency Syndromes **88**(1): 19-30.

Simbayi, L. C., S. C. Kalichman, S. Jooste, C. Cherry, S. Mfecane and D. Cain (2005). "Risk factors for HIV-AIDS among youth in Cape Town, South Africa." AIDS Behav **9**(1): 53-61.

Slabbert, M., F. Venter, C. Gay, C. Roelofsen, S. Lalla-Edward and H. Rees (2017). "Sexual and reproductive health outcomes among female sex workers in Johannesburg and Pretoria, South Africa: Recommendations for public health programmes." BMC Public Health **17**(Suppl 3): 442.

Stephenson, R., A. de Voux and P. S. Sullivan (2011). "Intimate Partner Violence and Sexual Risk-taking among Men Who Have Sex with Men in South Africa." West J Emerg Med **12**(3): 343-347.

Straus, S. E., A. Wald, R. G. Kost, R. McKenzie, A. G. Langenberg, P. Hohman, J. Lekstrom, E. Cox, M. Nakamura, R. Sekulovich, A. Izu, C. Dekker and L. Corey (1997). "Immunotherapy of recurrent genital herpes with recombinant herpes simplex virus type 2 glycoproteins D and B: results of a placebo-controlled vaccine trial." J Infect Dis **176**(5): 1129-1134.

SWPSSES (2013). Estimating the size of the sex worker population in South Africa, 2013. Sex worker population size estimate study (SWPSES).

TB HIV Care (2018). "Viral hepatitis C initiative for key populations in South Africa. Findings summary brief. Vol. 79. Cape Town: TB HIV Care.".

Todd, J., J. R. Glynn, M. Marston, T. Lutalo, S. Biraro, W. Mwita, V. Suriyanon, R. Rangsin, K. E. Nelson, P. Sonnenberg, D. Fitzgerald, E. Karita and B. Zaba (2007). "Time from HIV seroconversion to death: a collaborative analysis of eight studies in six low and middle-income countries before highly active antiretroviral therapy." AIDS **21 Suppl 6**: S55-63.

Toni, T., D. Welch, N. Strelkowa, A. Ipsen and M. P. Stumpf (2009). "Approximate Bayesian computation scheme for parameter inference and model selection in dynamical systems." J R Soc Interface **6**(31): 187-202.

Tronstein, E., C. Johnston, M.-L. Huang, S. Selke, A. Magaret, T. Warren, L. Corey and A. Wald (2011). "Genital shedding of herpes simplex virus among symptomatic and asymptomatic persons with HSV-2 infection." Jama **305**(14): 1441-1449.

Tsondai, P. R., L. S. Wilkinson, A. Grimsrud, P. T. Mdlalo, A. Ullauri and A. Boulle (2017). "High rates of retention and viral suppression in the scale-up of antiretroviral therapy adherence clubs in Cape Town, South Africa." J Int AIDS Soc **20**(Suppl 4): 21649.

Tucker, A., J. Liht, G. de Swardt, G. Jobson, K. Rebe, J. McIntyre and H. Struthers (2013). "An exploration into the role of depression and self-efficacy on township men who have sex with men's ability to engage in safer sexual practices." AIDS Care **25**(10): 1227-1235.

Tucker, A., J. Liht, G. de Swardt, G. Jobson, K. Rebe, J. McIntyre and H. Struthers (2014). "Homophobic stigma, depression, self-efficacy and unprotected anal intercourse for peri-urban township men who have sex with men in Cape Town, South Africa: a cross-sectional association model." AIDS Care **26**(7): 882-889.

UNAIDS-AIDSinfo UNAIDS-AIDSinfo. Available at <http://aidsinfo.unaids.org/>.

United Nation, Department of Economic and Social Affairs and P. Division (2015). UN World Population Prospects: The 2015 Revision.

Wald, A., D. Carrell, M. Remington, E. Kexel, J. Zeh and L. Corey (2002). "Two-day regimen of acyclovir for treatment of recurrent genital herpes simplex virus type 2 infection." Clin Infect Dis **34**(7): 944-948.

Wald, A., L. Corey, B. Timmler, A. Magaret, T. Warren, S. Tyring, C. Johnston, J. Kriesel, K. Fife, L. Galitz, S. Stoelben, M. L. Huang, S. Selke, H. P. Stobernack, H. Ruebsamen-Schaeff and A. Birkmann (2014). "Helicase-primase inhibitor pritelivir for HSV-2 infection." N Engl J Med **370**(3): 201-210.

Wald, A., J. Zeh, G. Barnum, L. G. Davis and L. Corey (1996). "Suppression of subclinical shedding of herpes simplex virus type 2 with acyclovir." Ann Intern Med **124**(1 Pt 1): 8-15.

Wald, A., J. Zeh, S. Selke, R. L. Ashley and L. Corey (1995). "Virologic characteristics of subclinical and symptomatic genital herpes infections." N Engl J Med **333**(12): 770-775.

Wald, A., J. Zeh, S. Selke, T. Warren, R. Ashley and L. Corey (2002). "Genital shedding of herpes simplex virus among men." J Infect Dis **186 Suppl 1**: S34-39.

Wald, A., J. Zeh, S. Selke, T. Warren, R. Ashley and L. Corey (2002). "Genital shedding of herpes simplex virus among men." J Infect Dis **186 Suppl 1**(Supplement_1): S34-39.

Wald, A., J. Zeh, S. Selke, T. Warren, A. J. Ryncarz, R. Ashley, J. N. Krieger and L. Corey (2000). "Reactivation of genital herpes simplex virus type 2 infection in asymptomatic seropositive persons." N Engl J Med **342**(12): 844-850.

Weller, S. and K. Davis (2001). "Condom effectiveness in reducing heterosexual HIV transmission." Cochrane Database Syst Rev(3): CD003255.

Zanoni, B. C., T. Sibaya, C. Cairns, S. Lammert and J. E. Haberer (2017). "Higher retention and viral suppression with adolescent-focused HIV clinic in South Africa." PLoS One **12**(12): e0190260.

Zhang, Y., J. M. Fogel, X. Guo, W. Clarke, A. Breaud, V. Cummings, E. L. Hamilton, A. Ogendo, N. Kayange, R. Panchia, K. Dominguez, Y. Q. Chen, T. Sandfort and S. H. Eshleman (2018). "Antiretroviral drug use and HIV drug resistance among MSM and transgender women in sub-Saharan Africa." AIDS **32**(10): 1301-1306.
